# Transmission of Single HIV-1 Genomes and Dynamics of Early Immune Escape Revealed by Ultra-Deep Sequencing

**DOI:** 10.1371/journal.pone.0012303

**Published:** 2010-08-20

**Authors:** Will Fischer, Vitaly V. Ganusov, Elena E. Giorgi, Peter T. Hraber, Brandon F. Keele, Thomas Leitner, Cliff S. Han, Cheryl D. Gleasner, Lance Green, Chien-Chi Lo, Ambarish Nag, Timothy C. Wallstrom, Shuyi Wang, Andrew J. McMichael, Barton F. Haynes, Beatrice H. Hahn, Alan S. Perelson, Persephone Borrow, George M. Shaw, Tanmoy Bhattacharya, Bette T. Korber

**Affiliations:** 1 Theoretical Biology, Los Alamos National Laboratory, Los Alamos, New Mexico, United States of America; 2 Department of Microbiology, University of Tennessee, Knoxville, Tennessee, United States of America; 3 Department of Mathematics and Statistics, University of Massachusetts, Amherst, Massachusetts, United States of America; 4 SAIC-Frederick, National Cancer Institute, Frederick, Maryland, United States of America; 5 Department of Medicine, University of Alabama at Birmingham, Birmingham, Alabama, United States of America; 6 Weatherall Institute of Molecular Medicine, Oxford University, Oxford, United Kingdom; 7 Duke University Medical Center, Durham, North Carolina, United States of America; 8 The Jenner Institute, University of Oxford, Compton, United Kingdom; 9 The Santa Fe Institute, Santa Fe, New Mexico, United States of America; University of California San Francisco, United States of America

## Abstract

We used ultra-deep sequencing to obtain tens of thousands of HIV-1 sequences from regions targeted by CD8+ T lymphocytes from longitudinal samples from three acutely infected subjects, and modeled viral evolution during the critical first weeks of infection. Previous studies suggested that a single virus established productive infection, but these conclusions were tempered because of limited sampling; now, we have greatly increased our confidence in this observation through modeling the observed earliest sample diversity based on vastly more extensive sampling. Conventional sequencing of HIV-1 from acute/early infection has shown different patterns of escape at different epitopes; we investigated the earliest escapes in exquisite detail. Over 3–6 weeks, ultradeep sequencing revealed that the virus explored an extraordinary array of potential escape routes in the process of evading the earliest CD8 T-lymphocyte responses – using 454 sequencing, we identified over 50 variant forms of each targeted epitope during early immune escape, while only 2–7 variants were detected in the same samples via conventional sequencing. In contrast to the diversity seen within epitopes, non-epitope regions, including the Envelope V3 region, which was sequenced as a control in each subject, displayed very low levels of variation. In early infection, in the regions sequenced, the consensus forms did not have a fitness advantage large enough to trigger reversion to consensus amino acids in the absence of immune pressure. In one subject, a genetic bottleneck was observed, with extensive diversity at the second time point narrowing to two dominant escape forms by the third time point, all within two months of infection. Traces of immune escape were observed in the earliest samples, suggesting that immune pressure is present and effective earlier than previously reported; quantifying the loss rate of the founder virus suggests a direct role for CD8 T-lymphocyte responses in viral containment after peak viremia. Dramatic shifts in the frequencies of epitope variants during the first weeks of infection revealed a complex interplay between viral fitness and immune escape.

## Introduction

HIV-1 evolves with extraordinary rapidity: high replicative error rates, large effective population sizes, high levels of recombination and enormous replication rates [1], [2] all combine to generate a fertile field for immune selection [3], [4], [5], [6]. Replicating viral populations typically remain in genital/rectal mucosa and associated lymph nodes for about 10 days post-infection, then begin to spread via the blood and to expand exponentially. Peak viral load occurs an average of 24 days from transmission [7]; the plasma viral load declines thereafter to a moderately stable “set-point” level, which is predictive of rates of progression to AIDS and disease outcome [8]. Understanding the timing and impact of the initial immune response to HIV infection and the viral escape from that response, within the context of the earliest biological events characteristic of acute infection, is critical for the development of reasoned vaccine strategies.

The first HIV-specific cytotoxic-T-lymphocyte (CTL) responses can be detected before peak viremia [9]; they are among the earliest selective forces acting on viral evolution [10], [11], [12], and may contribute to initial containment of the virus [4]. We reasoned that detection of viral evolution at recognized epitopes during acute infection would indirectly reveal the onset of effective immune responses. Accordingly, we investigated the mutational dynamics of CTL immune escape using 454-pyrosequencing [13] in 3 acutely infected subjects. These three subjects (SUMA, WEAU, and CH40) have been well characterized in previous work (Tables S1 and S2 summarize both published and additional data relating to these subjects; S1 summarizes the relevant clinical data, and S2 the immunological and conventional sequencing data). The previous studies allowed us to select specific viral genomic regions for intensive investigation. We targeted regions spanning 4 epitopes with very early recognition and escape [9], [3], [4], [5]. For contrast, and as a control, we sequenced the Envelope V3 region (which is not under immune pressure in early infection) in each subject. By obtaining very large numbers of sequences spanning the earliest-escaping epitopes, we were able to greatly strengthen the evidence for single virus initiation of acute infection following mucosal exposure, to detect low-frequency escape mutants that subsequently became predominant, and to calculate selective pressures and rates of escape in acute infection.

## Results

We obtained thousands of sequences from 3 early time-points for each of the subjects CH40, SUMA, and WEAU, and chronic-infection sequences from WEAU's transmitting partner RIER (Table 1). We used published conventions for numbering days in each subject for ease of comparison to prior relevant publications: In WEAU and SUMA, day 0 (d0) marks the onset of symptoms; in CH40, d0 denotes the screening sample (symptom onset was self-reported to be 2 days earlier; Table S1).

**Table 1:** Number of 454 sequences obtained from each sample.

Patient		*Time-point (days post screening)*				
	*Region*	Sequence count per time-point (total cleaned reads)				
**CH40**		*d0*	*d16*	*d45*		*control*
	***Nef SL10***	4,048	7,754	1,873		11,318
	***V3***	3,354	1,557	2,487		93,793
**SUMA**		*d5*	*d20*	*d41*		*control*
	***Rev QL9***	31,183	8,150	10,350		3,360
	***Tat FY16***	3,276	7,041	378,278		2,653
	***V3***	40,694	27,081	16,277		5,939
**WEAU**		*d10*	*d20*	*d30*	*donor*	*control*
	***gp160 AY9***	23,459	11,835	5,910	18,952	15,140
	***V3***	4,001	3,755	4,062	4,001	8,791

These sequences are the ones that remained for analysis after cleaning steps to correct likely 454 error, and filtering out short fragments and sequences with frame-shifting indels, stop codons and multiple insertions and deletions.

### Evidence for establishment of infection by a single virus and estimated times to the most recent common ancestor

Earlier work (using conventional SGA sequencing [11]) suggested that in each of these cases, productive infection was established by only one virus per patient. We reassessed this hypothesis under the much more intense scrutiny of ultra-deep sequencing (approximately 3,000 to 40,000 sequences per first time-point compared to 29 to 44 sequences per first time-point [11]). We analyzed the 454 sequences from the earliest time point (before overt immune selection) in each of the three cases to determine if the observed sequence diversity was consistent with a single-virus origin of infection.

**Figure 1:**
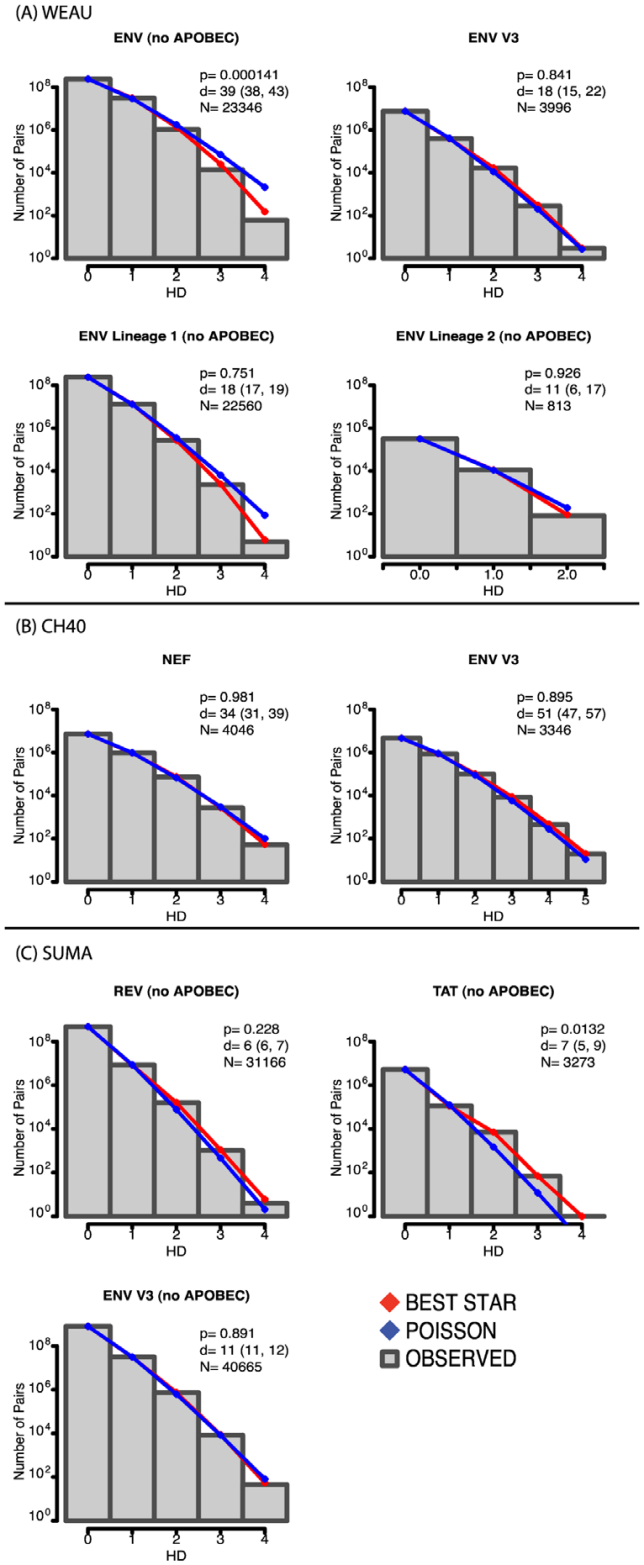
Histogram of pairwise Hamming distance (HD) frequency counts from the earliest samples compared to model predictions. The red line represents expected counts if the sample followed a star-like phylogeny; the blue line represents the expected counts of the best-fitting Poisson distribution. Goodness of fit p-values (p) (low p-values indicate a statistically significant divergence from a Poisson distribution), estimated days since the most recent common ancestor (d) (95% confidence interval in parentheses), and the number of sequences analyzed (N) are noted in legend (see also Table 1). As we observed previously, for individuals with HIV sequences with increased apobec-context G-to-A mutations [14], [16], columns containing apobec-context G-residues must be eliminated for the data to fit a Poisson. A) Subject WEAU: the ENV V3 region (top right) does not diverge significantly from a Poisson distribution; when the ENV epitope region are analyzed all together, they diverge from a Poisson (top left), but can be split into two Poisson-consistent lineages (bottom). B) subject CH40: both the NEF epitope region (left) and ENV V3 region (right) are consistent with a Poisson. C) subject SUMA: the REV epitope and ENV V3 regions (left) were consistent with a Poisson model, but the TAT epitope was not (see text). Additional details regarding the modeling are provided in Table S4.

**Figure 2:**
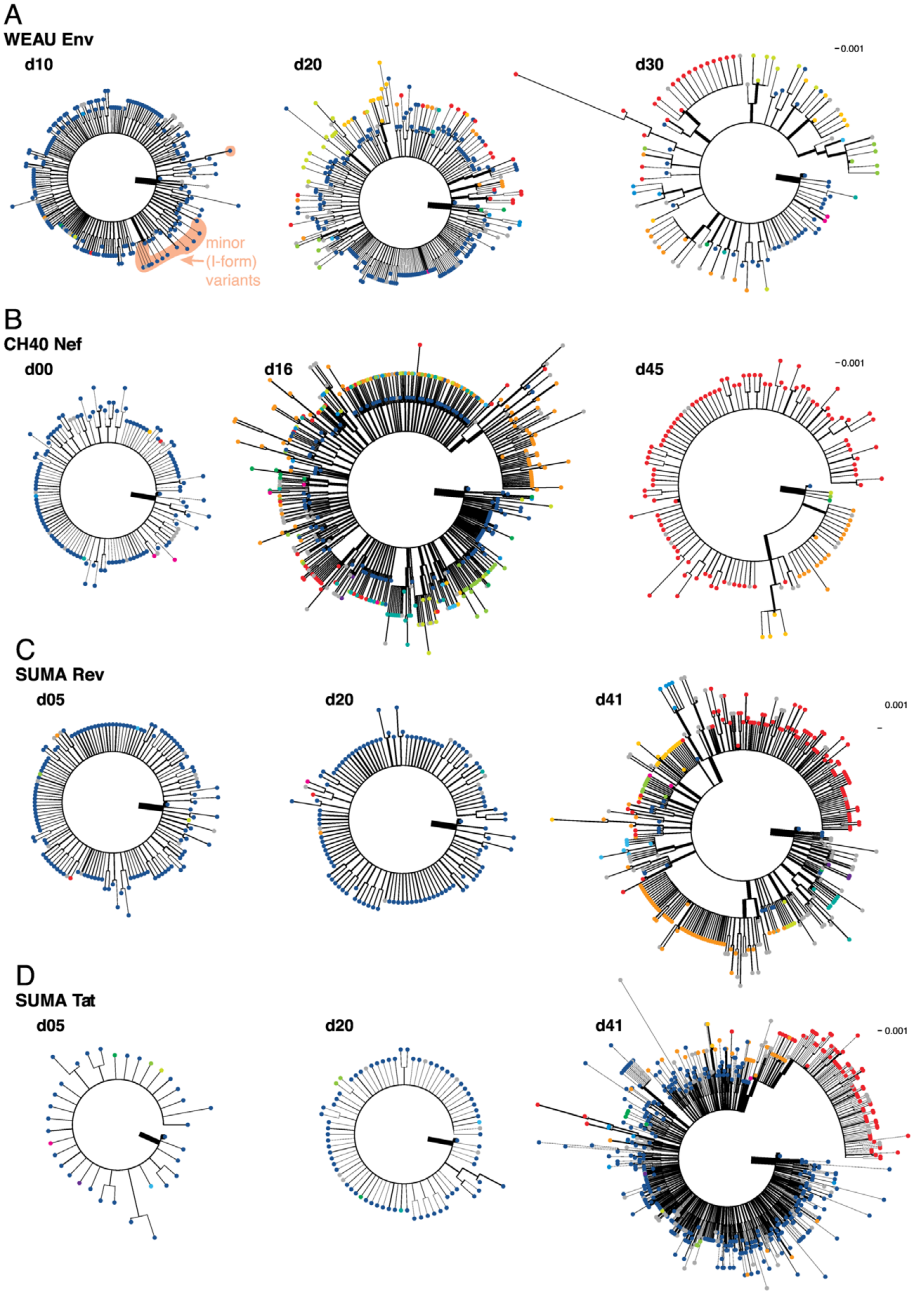
Maximum Likelihood Phylogenies from DNA sequences of epitope regions. (A) WEAU env, (B) CH40 nef, (C) SUMA rev, and (D) SUMA tat. Separate trees represent sample time points. Circles at terminal nodes represent epitope variants (colors as in Fig. 5). Trees are rooted on the transmitted variant. Branch widths are proportional to the log of variant frequency; sequences identical to an ancestral node have a negligible non-zero branch-length provided to display frequency.

When infection has been established by a single virus and mutations have not undergone positive selection, the frequencies of sequence variants relative to the most common form are expected to conform to a Poisson model of mutation and a star phylogeny [11], [14]; additionally, the time to the most recent common ancestor (tMRCA) can be estimated based on a model that incorporates the mutation rate, generation time, and basic reproductive ratio [14]. In both CH40 and SUMA, the earliest-sample data from both the epitope regions and the V3 regions were consistent with a Poisson distribution and star phylogeny (Fig. 1B,C). This suggests that in each of these patients, all the sampled viruses shared a recent common ancestor, which implies that infection was homogeneous (established by a single transmitted virus). A single variant (the inferred transmitted virus) dominates the sample, and rare variant forms are only one or two mutations removed from it (see Table S3 for sequences clustered by similarity; format of the table is annotated in Fig. S1). The phylogenetic trees from the first time point (left hand side of Fig. 2 B, C and D, and Fig. S2 B, C) illustrate the accumulation of random mutations away from the transmitted form over short time-spans in the absence of strong selection. The vast majority of the viral sequences sampled at the first time point carry the transmitted form of the epitope (dark blue dots in Fig. 2).

For subject WEAU, the data were slightly more complicated: distances between the sequences from the earliest sample (d10) fit a Poisson distribution for the Env V3 region data (Fig. 1A), but not for the Env epitope region data, where a single C-to-T nucleotide mutation (causing a T-to-I amino acid change well outside the epitope; Table S3) was present in 3.5% of sequences. When the WEAU d10 sequences were partitioned by presence or absence of this mutation, each of the two sub-lineage distributions was consistent with a Poisson distribution (Fig. 1A) and a star phylogeny. The thickness of each branch in Fig. 2 is proportional to the log of the total number of descendants from that branch; apart from the transmitted form, the only substantial branch among the first time point phylogenetic trees (Fig. 2), is that which leads to most of the WEAU minor I-form variants, indicated in Fig. 2A.

The occurrence of two highly similar but distinct lineages in WEAU could have resulted either from co-transmission of two closely related viruses, or from outgrowth of a random mutation that occurred in an early post-transmission replication [15]; while our data do not allow us to decide conclusively between these alternatives, several lines of reasoning argue for a single transmission. First, in a collection of such deep samples, the star phylogeny is not expected to hold exactly, because preservation of early mutations is likely to occur stochastically at a low frequency. Without differential survival of extant forms, a mutation with 3–4% prevalence would have to have arisen when the viral population size was about 30; this implies (if early viral growth is a fast exponential) that the mutation rate was large enough to cause a mutation in our sequenced region of 130 base pairs once in about 30 replications. For HIV, the expected mutation rate is about 2×10^−5^ per base per generation [1], and so the probability of this happening at one out of 130 bases is about 0.08. Given that we are studying 7 different regions, the observation of one such early stochastic event is consistent with expectation. Furthermore, the T-form was the most common form in both WEAU and his transmitting partner RIER, and although the I-form was present in RIER's diverse population, it was found at very low frequency (0.001); co-transmission of such a rare form that is so nearly identical to the dominant form is far less probable than the single mutation arising in WEAU in an early replication cycle. Finally, because the two WEAU lineages both conform to a Poisson distribution, we can estimate a tMRCA for each of them. The minority I-form lineage appears younger than the majority lineage: the tMRCA estimate of the major epitope-region (T-form) lineage and the V3 region were comparable (18 days, CI 16–19; 18 days, CI 16–22), but the minor lineage (I-form) tMRCA was shorter (11 days, CI 7–17), suggesting it arose after infection. WEAU's HIV exposure reportedly occurred 30 days before the earliest sample [3]. The less-than-30-day tMRCA estimates for both the V3 and the T-form epitope lineages (∼ 18 days; Fig. 1A, Table S4) could be explained either by a prolonged eclipse period before viral expansion began, purifying selection retarding the accumulation of mutations [16], or variation in viral population parameters (e.g. mutation rate or *R*_0_ [14]).

Because selective pressure had not materially affected the sequence distributions in the first time-point samples from SUMA and CH40 (as evinced by the Poisson distributions, Fig. 1B, C), tMRCA estimates were also possible in these subjects. For SUMA, the tMRCA was 5–12 days before the d5 sample, consistent with clinical data [the d5 sample was Fiebig stage II, and sampled prior to peak viremia (Table S1)]. For CH40, the first available sample (d0) was near peak viremia (Table S1), and the tMRCA estimates were discordant between regions: a plausible 31–38 days for the Nef epitope region, but a longer-than-expected 46–56 days for V3. This difference could result from variability in mutation rates between different viral genomic regions, or possibly from an unidentified class of 454 pyrosequencing error in the V3 region sequences (we took careful measures to account and control for errors, however, as described in the methods).

### Action of Natural Selection upon Epitope Variants

**Figure 3:**
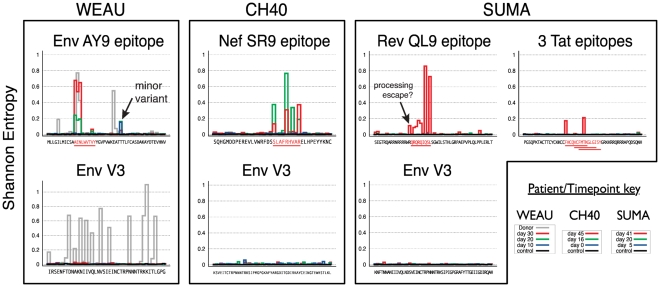
Site-by-site entropy values for inferred protein sequences. Defined epitopes are in red and underlined. Shannon entropy values are distinctly elevated in epitope regions. The position of the WEAU Env minor “I-form” variant (see text) is indicated (arrow). SUMA Rev sequences include one additional position with increased entropy, immediately to the left (5′) of the epitope (arrow), which is likely to be a processing mutation.

**Figure 4:**
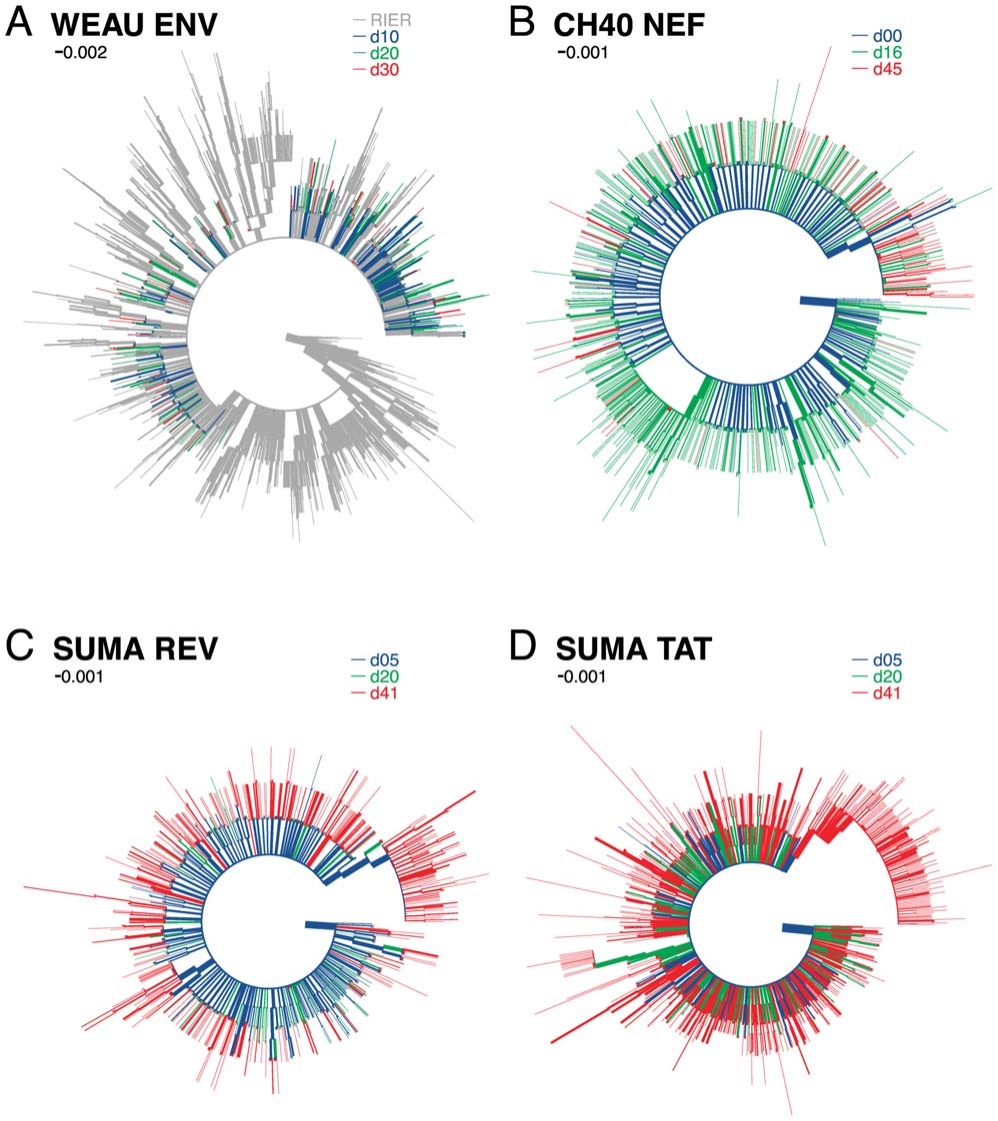
Maximum likelihood phylogenetic trees including all three time points for each subject. (A) WEAU and RIER Env, (B) CH40 Nef, (C) SUMA Rev, and (D) SUMA Tat. First time-point variants (blue), second, green; third, red; RIER (WEAU's partner and source of infection), gray. The tree in (A) is rooted on a non-transmitted variant in RIER; the other 3 trees are rooted by transmitted variant.

Shannon entropy provides a simple measure of local variation in DNA and protein sequence alignments that reflects in a single value both the number of variants and their distribution [17]. In all subjects, entropy was uniformly very low throughout the sequence in the initial time-point samples; in later samples, sequence entropy increased markedly in positions within the known CD8+ T-cell epitopes, but essentially nowhere else (Fig. 3), the sharp contrast revealing the potent immunological selective pressure focused sharply upon the epitopes. In non-epitope regions and in V3, in contrast, entropy remained low throughout early infection. The only two exceptions were the WEAU minor lineage discussed previously, and an R-to-K change immediately adjacent to the SUMA Rev epitope (both are indicated in Fig. 3); because of its immediate proximity to the epitope the latter change is a candidate for processing escape. Over the time-period studied, 454-sequencing allowed detection of 54-99 epitope variants per subject, instead of the mere 2-7 variants observed with conventional sequencing (Table 2). In contrast, 454 sequences from a chronic HIV-1 infection case (WEAU's transmitting partner, RIER), showed extensive diversity throughout both the epitope and V3 regions (Figs. 3, 4).

**Table 2:** The total number of epitope variants, and the total number of sequences available.

Subject	Gene	Epitope	Latest timepoint compared	SGA sequence Count	SGA Epitope Count	454 sequence count	454 Epitope Count	Repeated Variant Count
WEAU	Env	AY9	d30	102	7	41,068	54	40
CH40	Nef	SR9	d45	23	5	13,655	76	40
SUMA	Rev	QL9	d41	150	5	49,635	66	49
SUMA	Tat	FK10, VI10, & FY10	d41	59	2	388,326	99	55

This summary includes all sequences from samples spanning the period of study used for 454-pyrosequencing, comparing conventional (SGA) sequencing and 454 pyrosequencing. “SGA sequence Count” refers to the number of individual sequence isolates determined by SGA sequencing in previous studies; “SGA Epitope Count” gives the number of unique amino-acid sequences at the epitope site as determined by SGA sequencing; “454 sequence count” and “454 Epitope Count” give the corresponding (and much larger) numbers determined in this study; “Repeated Variant Count” refers to the number of epitope-site peptide sequences that were found more than once among the 454 sequences.

### Evolutionary Trajectory of Early Immune Escape

**Figure 5:**
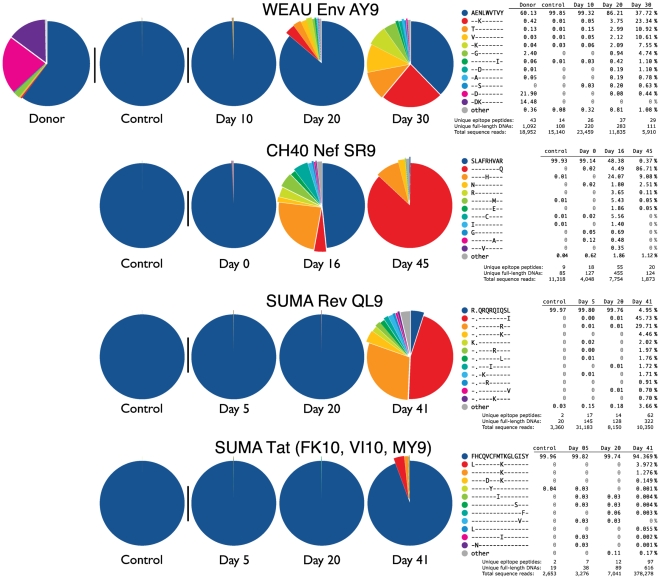
Epitope variant frequencies. Epitopes are organized as follows: inferred infective sequence (deep blue), the most common variant at the final time-point (red), and all of the 12 most common variants sorted and colored by their frequency at the last time-point.

The extensive sampling of 454-sequencing allowed us to track evolutionary trajectories of individual epitope variants in detail. By the second time point sampled in WEAU and CH40, and the third time point in SUMA, the viral population diversity had expanded dramatically and variants had become structured into clusters, each comprising a single-escape-mutation majority form surrounded by far less common single and double secondary mutations scattered throughout the region sequenced (Fig. 2 and Table S3). The phylogenetic trees illustrate the emerging relationships between escape forms over time. In Fig. 2, each time point is considered separately, and each of the epitope variant forms is indicated by a colored dot using the same coloring scheme as Fig. 5, where the frequency and sequence of each epitope variant are shown. Clades forming around each of the selected epitope variants are clearly visible. In Fig. 4, all time points are combined in a single tree, and colors denote time points rather than epitope variants.

Of note, there is extreme variation in the viral sequences from WEAU's transmitting partner RIER, which were sampled during chronic infection near the time of transmission to WEAU (Fig. 4A, grey branches); nevertheless, the variants found in WEAU, even at later time points, appear to have evolved from a single founder (Fig. 4A), a further indication that multiple variants were not transmitted. The common within-epitope escape mutations (11 of the 12 most common) found in WEAU were also present in RIER at low frequencies (Table S3). This scattering of WEAU-derived sequences among RIER's vastly greater diversity (Fig. 4A, S3) superficially resembles multiple-virus transmission – but if multiple transmissions had occurred, and rare co-transmitted variants grew out at later time points following immune suppression of the majority form, a broad sampling of the diverse RIER forms would be expected in WEAU. Instead, the dominant earliest escape variants in WEAU were each a single mutation away from the transmitted form, suggesting they arose independently in WEAU. CH40 showed striking evidence of a post-transmission bottleneck between d16 and d45 (Figs. 4 and 5). In this subject, explosive diversification at d16 was followed by a narrowing of diversity, with the outgrowth of a few variants by d45 (Fig. 4B). In SUMA, neither region shows overt evidence of selective pressure until d41 (Fig. 4C and D). In contrast to the epitope-region phylogenetic trees, the V3 sequences show little phylogenetic structure and no evidence of selection (Figs. S2, S3).

Dramatic shifts in the relative frequency of escape forms were observed in all three patients, but at different times in the course of infection (earlier in CH40 than in WEAU and SUMA). In CH40, for example, the most common variant in the Nef SR9 epitope at d16, an R-to-H change at epitope position 5, became subdominant by d45, when the dominant sequence contained an R-to-Q change at position 9 (Fig. 5). In WEAU, the relative rankings of common escape forms were unchanged from day 20 to day 30 – but despite this early consistency, the dominant escape form at these two early time points (a N-to-K change at position 3), was rare by day 45, and at d70 was undetectable by conventional sequencing (based on information from earlier studies done using single genome amplification (SGA) sequencing, summarized in Table S2). Apart from the founder virus, the two next-most-common variants found at the AY9 epitope site in WEAU's partner RIER remained very rare or undetected in WEAU throughout the sampling period (Fig. 5). In SUMA, escape in the two mutating epitopes was found in a substantial fraction of sequences only in the last time point sampled for 454 sequencing; again, based on SGA sequencing at later time points (Table S2), the most common escape form in the d41 Tat sample was essentially replaced by another escape form by d69.

To explore whether there was serial selection of mutations in these epitopes, i.e. whether the first escape mutation served as a baseline form for added selected mutations, we grouped all variants of each major escape form as a lineage, and tested whether or not the frequencies of secondary mutations around each of the most-common selected mutations were Poisson-distributed. This was essentially true in all but one case, indicating that once the initial escape occurred, subsequent selection (e.g., acquisition of additional mutations for stronger escape or local compensatory mutations) was not the norm (Table S5). The only exception was found among SUMA Tat variants: the distribution of secondary mutations in the major lineage was non-Poisson, and the dominant escape form included two mutations instead of a single substitution (Fig. 5, Table S2, Table S5). The SUMA Tat region was also was the only region studied that contained overlapping epitopes, recognized by 3 distinct early CD8+ T-lymphocyte responses focused in this region [4]; thus the more complex pattern of early escape in SUMA Tat might have been a consequence of multiple distinct selective pressures from distinct T-lymphocyte responses. The measured responses to the two subdominant epitopes in Tat, however, were of low magnitude and avidity, and so might not be expected to appreciably drive viral sequence change (ref. [5]; also see Table S1). Thus an alternative hypotheses to explain the observed acquisition of two amino acid substitutions is that either both were required to fully abrogate epitope processing, or that an additional compensatory amino acid change was required to enable the virus to tolerate the escape mutation.

### The Dynamics of Early Immune Escape

**Figure 6:**
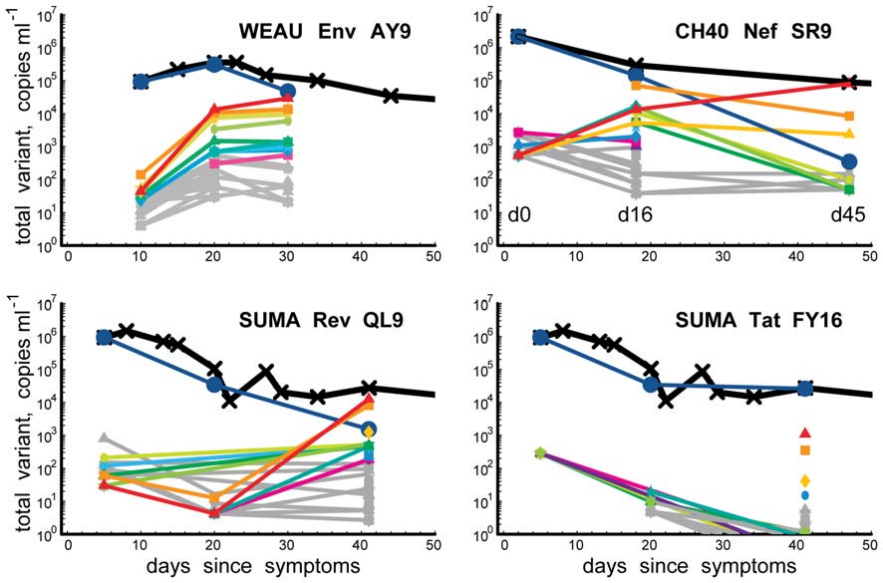
Viral dynamics: Variant counts in relation to viral load. Total numbers of viral variants per ml, estimated from 454 read frequencies and viral load. Viral load in black, measurement dates indicated. Solid blue lines denote transmitted virus; epitope colors match Fig. 5.

**Figure 7:**
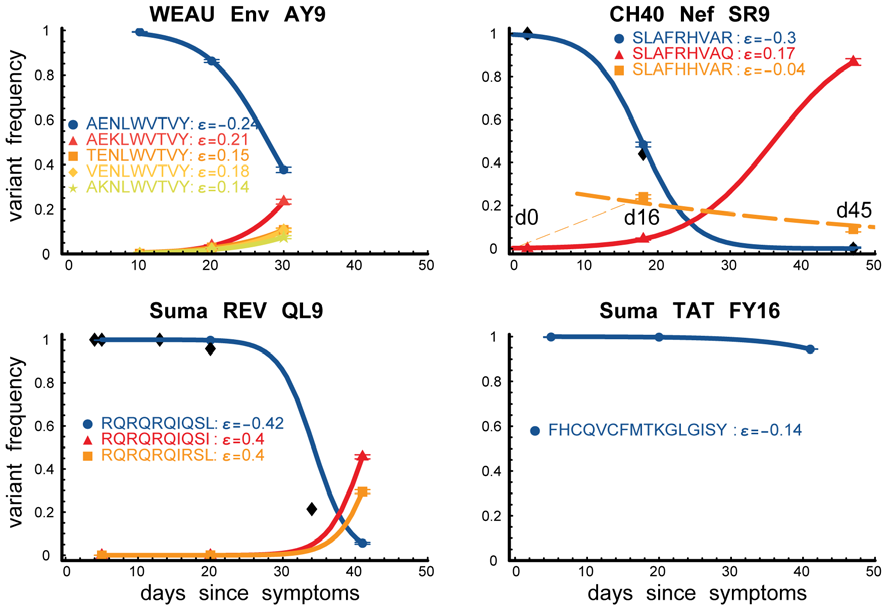
Viral dynamics: Estimated accumulation rates of most−common variants. Estimated growth rates of most-common variants. Colors match Fig. 5; black diamonds denote founder-sequence frequency previously measured by SGA [4]; horizontal bars, 95% confidence-intervals; and 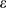, the rate of accumulation of variants. The dashed line in CH40 denotes accumulation of the SLAFHHVAR in the first 2 weeks of the infection (estimated minimal escape rate, 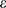 = 0.44 day^−1^, doubling time 1.6 days). Our previous SGA-sequence-derived estimates of the founder sequence loss rate were lower, 0.13 day^−1^ (doubling time 5.3 days) and 0.10 day^−1^ (doubling time 6.9 days) for WEAU Env AY9 and SUMA TAT epitopes, respectively [4].

Longitudinal sampling enabled us to infer rates of variant accumulation [4], [18], [19]; within a single epitope, the accumulation rates of different variants varied dramatically, and the spread of variant rates varied from epitope to epitope (Table S6, Fig. S4). In some cases, the proportion of particular variants increased at the expense of the founder virus while total viral load decreased (e.g. Fig. 6A and B). In other cases, an increase in the population frequency of escape-mutant viruses coincided with a rise in total viral load (Fig. 6C, SUMA d20-d41). Parallel increases in the relative frequencies of most variants in the viral population at the expense of the founder virus (e.g. WEAU AY9, Fig. 6) are likely due to preferential killing of founder virus by epitope-specific CD8+ T-lymphocyte responses; such relative increases will not involve increases in the absolute number of escape-variant virions. Indeed, our analysis showed that the total number of some viral variants remained virtually unchanged (Fig. 6) despite increases in their population frequency over the same time period (Figs. 5 and 7). Increases in the absolute number of some other viral variants, however, paralleled rises in viral load, suggesting that, prior to escape, specific T-lymphocyte responses directed to susceptible forms of these epitopes had effectively suppressed viral replication. In some cases (e.g., SUMA Rev QL9) the rate of increase in the total number of variant sequences was large (approximately 0.4 per day, corresponding to a doubling time of 1.7 days; Fig. 7C), even when total viral load was relatively stable (Fig. 6C). This observation suggests that, at least in these subjects, substantial viral replication continued through the decline from peak viremia and the approach to viral set point; this implies that the viral decline after the peak and the constant viral set point cannot be solely due to depletion of CD4+ target cells; and, since differences in expansion/decay rates are correlated with immune targeting, it is likely that, during these periods, cellular immunity is continuously involved in the control of the virus.

For every patient, we estimated the rate of loss of the founder virus sequence as well as the rate of accumulation of several viral variants that reached high frequency over the course of infection (Fig. 7). For 2 of the epitopes studied here, we previously estimated the rate of loss of the founder virus (or the rate of accumulation of all escape variants) from SGA sequences (epitopes Nef SR9 in CH40 and Rev QL9 in SUMA, [4]. For the other two other epitopes, the rate of escape was calculated from the changes in the frequency of variants based on epitope frequencies from available conventional sequence data (WEAU Env AY9 and SUMA TAT [5], applying the methods outlined previously [4]. 454 sequencing data provided estimates of the loss rate of the transmitted virus that were more precise and 25 to 50% larger than the minimal estimates derived from SGA or conventional sequencing data (Fig. 7).

The rate of accumulation of escape forms (Fig. 7) was used to estimate the timing of the start of immune selection (for details, see the Supporting Information). In the first WEAU sample (d10), non-synonymous mutations were already significantly enriched specifically within the epitope (Table S3); immune selection was estimated to have started 5 days earlier, 18 days prior to the sample with peak viremia. In CH40, selective pressure was again estimated to have begun about 5 days before the first available sample (d0); by d0, viral load was >2×10^6^/ml, and likely near its peak. In both of these cases, selection indicates CD8+ T lymphocyte responses were present and may have helped contain the transmitted virus, but escape forms rapidly accrued. In SUMA, d8 was at or near peak viremia, and the immunodominant response was to SUMA Tat FY10 [5]. Selection was estimated to have begun at d10 in FY10, but escape forms accumulated very slowly, perhaps due to constraints on the virus and fitness costs of escape; selection was not estimated to have begun in Rev QL9 until d18. The retarded escape from the Tat response(s) is also consistent with the hypothesis proposed above, that the virus may have needed 2 amino acid changes to fully achieve escape, rather than just one. Of the three patients, SUMA's viral load dropped most precipitously from the peak (Table S1, Fig. 6), and his CD4 count remained stable during the first 2 years of infection; it is possible that the combination of potent acute immune response, as originally proposed [5], coupled with the observed delay in viral escape seen here, contributed to early viral control.

### Reversion to Consensus

**Figure 8:**
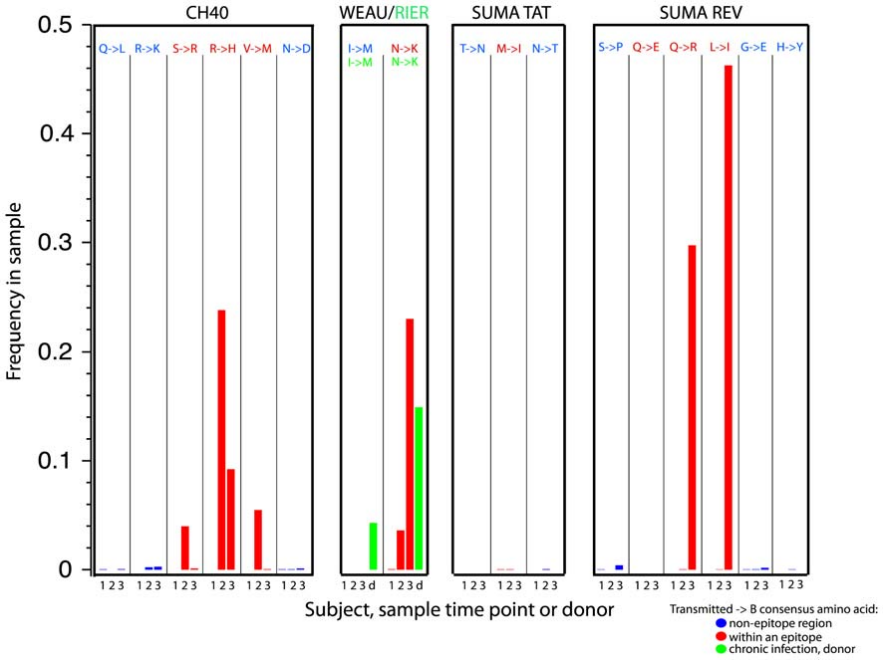
Comparison of consensus frequencies inside and outside of epitopes. Changes to consensus were much more frequent inside epitopes, and presumably these were rapidly selected for immune escape in part because they were relatively fit. Reversion to the consensus amino acids outside of the epitopes was never observed on the time scale studied here, but consensus forms were common by the time the subjects had reached chronic infection (RIER, shown here in green).

It has been shown that in some instances immune escape entails a fitness cost to the virus, and that, following transmission of an escape variant to an immunologically distinct host, reversion to subtype consensus amino acids can restore the lost viral replicative capacity [20]. This phenomenon has been intensively studied for HLA B*5701/B*5703-presented epitopes, since these alleles are strongly associated with viral control [21], but analysis of associations of other HLAs with mutational patterns at the population level suggests it may be a widespread phenomenon [22], [23]. To determine if rapid reversion towards the B subtype consensus was evident in the any of the regions under study here, we tracked mutational patterns in all positions where the transmitted form of the virus differed from the B subtype consensus amino acid. In 6 of 8 within-epitope positions where the transmitted virus differed from the B subtype consensus, the B subtype consensus was rapidly selected, suggesting the B-consensus form in these within-epitope positions was a reasonably fit way to impart immune escape (Table S7). In contrast, outside of the epitopes, there was no evidence for reversion to the subtype consensus in these early samples in any of the 35 positions where a non-consensus sequence was transmitted (Table S7). Among the 6 within-epitope toward-consensus mutations, 4 increased only transiently (Supporting Information). In chronically infected RIER, non-negligible replicating levels of B-consensus amino acids were found in 7 of the 8 positions where the within-patient consensus differed from the B subtype consensus (Fig. 8, Table S7), again indicating that the consensus form was not necessarily selected to fixation even when it did arise by mutation.

In a related consideration, we used V3 loop sequences to predict CXCR4 co-receptor usage [24] in SUMA and CH40, and found very low levels of CXCR4 candidate sequences in both subjects. The V3 region WEAU did not span enough of the loop for prediction, and transmitted virus in WEAU was a R5/X4 virus [11], Thus, like the immune escape forms, single base changes that give rise to predicted CXCR4-using variants are likely to be present from early in infection, and could be available for rapid expansion when selection pressure arises [25].

## Discussion

### Establishment of infection by a single virion?

This study provides a very deep view of the viral quasispecies through very narrow (∼ 150 bp) windows. We implemented careful clean up of the 454 data to minimize the impact of characteristic 454 sequencing errors on interpreting our data. The relative success of these measures is evident in our controls and in the biological appropriateness of the observed variation patterns in the post-clean up sequences, by three different measures: i) the very low level of background variation evident in the controls and earliest time point samples, ii) the striking contrast between the extensive diversity found within epitopes (as evident in the entropy plots) versus the very low levels of diversity found in the non-epitope regions; and iii) the accumulation of mutations in the earliest time points in a manner consistent with expectation based on modeling diversity using a Poisson distribution [11]; previously published estimates of the HIV error rate, reproductive ratio, and generation time [14]; and time from infection estimated based on independent data, both Fiebig staging and clinical histories (Table S1). We adapted our previously published model to enable exploration of very large data sets (ref. [14], and Giorgi et al., submitted).

Our earlier studies indicated that in roughly 80% of heterosexual transmission cases a single virus establishes infection [11], [26]. These previous studies, however, were typically limited to only several dozen SGA Env genes per subject. Therefore we selected three of these subjects for 454 sequencing: the analysis of these ultra-deep sequence sets presented here supported our previous findings and were consistent with single virus transmission [11]. For two of the three subjects, modeling based on an increase of sampling by 2–4 orders of magnitude over previous work (Table 1) failed to detect any plausible candidates for co-infecting variants; we therefore conclude that the HIV-1 infections of SUMA and CH40 were each established by no more than one viral genome. We also strongly favor the hypothesis of single transmission for the third subject, WEAU, for the reasons outlined in the results section; we emphasize, however, that although these points make a persuasive argument for single variant transmission in WEAU, they are not a proof.

Single-variant transmission is more common in individuals exposed heterosexually to HIV, compared to those with MSM or IV risk factors [27], [28]. This may be an important factor to consider in interpreting the results of future vaccine and prophylaxis trials. The genetic bottleneck observed in CH40 between d16 and d45 raises the possibility that immune selection during the first few months of infection may mask earlier diversity. Hence studies with sampling at, for example, 6-month intervals, may sample virus populations that have already been significantly distorted by selection. Further studies will be needed to fully resolve this issue. A profound bottleneck has also been recently reported in an HCV transmission study using ultradeep sequencing on early infection samples [29].

### Role of T-lymphocyte pressure in early infection

In the epitopes selected for this study, escape was very rapid, dozens of within-epitope escape routes were explored, and the transmitted epitope persisted at all time points studied, although in the CH40 SR9 epitope it had diminished to very low levels by day 45, suggesting effective population turnover. Recent reports of 454-sequences of SIV isolated from macaques during CD8+ T-lymphocyte escape in acute infection suggests that this situation is paralleled in SIV infection of nonhuman primates [30], [31]. We observed many shifts in the relative frequency of epitope variants (Figs. 5 and 6), which have several possible explanations. First, the immunological landscape may fluctuate, altering the selective forces acting on the virus: such changes might include de novo T-lymphocyte responses to escape forms, alterations in clonal composition and relative immunodominance, or shifting inhibitory functions of the epitope-specific responses over time as the immune response matures. Second, changes in replicative fitness may arise, for example as a result of compensatory mutations [32]. Finally, dynamic/stochastic effects might be responsible, i.e., a less-effective immune escape mutation might initially be more common due to random mutational events that preceded immune selection [16].

A pool of uncommon but replicating minor variants could provide the virus with many paths of escape from a particular immune response; the most common escape paths are likely to be relatively fit and to reflect a balance between replicative fitness and immune susceptibility. While it is not possible to block all escape routes in such a scenario, still it may be possible by vaccination with the most common variants of an epitope to inhibit rapid adoption of fit variants. This hypothesis is important for our mosaic vaccine design strategy [33]. The first results from mosaic vaccine studies in macaques [34], [35] show significantly more cross-reactive responses to the common variants of epitopes. The most common variants are included by design in the polyvalent mosaic vaccine; it remains to be seen whether this increased depth will translate to better control of the virus in a vaccine context. It is encouraging to note, however, that cross-reactive responses have been associated with slower disease progression in HIV-1 infection [36]. It is important to recognize that we focused on epitopes known from prior work to be subject to rapid escape; other CD8+ T-lymphocyte epitopes within these same individuals showed more gradual escape [9], [3], [4], [5] and may be under greater fitness constraints, less intense immune selective pressures, or both (Ferrari, et al. 2010, submitted).

Within epitopes, immune escape mutations towards the subtype consensus occurred rapidly, but were transient in 4/6 cases, perhaps reflecting a changing balance of replicative fitness and immune potency. We found a complete absence of such reversions in positions that were not under immune pressure at these early time points (Table S7). There was no evidence for a rapid reversion to the subtype consensus due to viral fitness alone – in the regions we studied, any fitness gain due to acquisition of the consensus residue [37] was insufficient to drive rapid selection.

In chronically infected RIER, B-consensus residues generally occurred at low frequencies in positions where the patient-consensus differed from the subtype-consensus. For comparison, we re-examined data from 4 additional chronic-infection subjects previously studied by 454 sequencing of the V3 region [25], finding a similar pattern in positions where patient-consensus differed from subtype-consensus (Table S8). These observations suggest that mutations towards consensus arise frequently and may be generated continuously during chronic infection, providing a reservoir of replication-competent viruses that can expand once immune selection begins to target majority forms (as was observed in the acute cases studied here). These combined data also suggest that, in early infection, reversion solely due to replicative fitness is atypical (although specific sites outside of the regions studied here may have a different dynamic), and that, in chronic infection, mutation towards the consensus is a dynamic process involving sustained turnover between consensus and mutant forms.

In summary, 454 sequencing provided epitope escape-rate estimates that were more precise and 25–50% faster than the minimal rates we derived from SGA data (Fig. 7; Text S1, Section IV), showing that the immune responses to these individual epitopes were even more potent than we showed previously [4]. Rates of escape acquisition and of loss of transmitted virus suggest CD8+ T lymphocytes are likely to have a direct role in virus containment early in natural infection, providing further impetus for continued exploration of vaccines that induce CD8+ T-lymphocyte responses [38], [34], [35].

## Materials and Methods

### Ethics Statement

This study was conducted according to the principles expressed in the Declaration of Helsinki. It was approved by the Institutional Review Boards of the University of Alabama at Birmingham, Rockefeller University, Duke University, and the University of North Carolina. All patients provided written informed consent for the collection of samples and subsequent analysis.

### Summary of Viral DNA amplification, sequencing, and sequence processing

To facilitate quantitative sampling of the viral population, cDNA generation from viral RNA and subsequent PCR reactions used high template volume, low cycle numbers, and multiple replicates that were pooled for sequencing (Fig. S5). As a control for 454 sequencing error, we also sequenced cloned DNA from the inferred transmitted virus [4], [12]. As expected, most 454-errors were insertions or deletions (indels) in homopolymer tracts. Our previous 454-cleanup approach [25] excluded such sequences, resulting in considerable data loss. We therefore added a sequence correction step to our processing pipeline [25], correcting common 454 indel error-patterns to match the transmitted virus.

### Study Subjects

Three subjects [SUMA0874 (*SUMA*), WEAU0575 (*WEAU*) and CHAVI-700-01-004-0 (*CH40*)] were selected for ultra-deep sequencing spanning early immune escape because their initial immune responses had been intensively surveyed, and the earliest selected mutations through all or part of their proteome had been mapped using conventional sequencing methods [9], [4], [5], [11]. At presentation, all three patients had symptoms of acute HIV-1 infection, were HIV-1 antibody negative, but tested positive for HIV RNA and/or P24 (hence in Fiebig stage I/II). Sampling days for each patient are presented here consistently with past publications: days following onset of symptoms (DFOSx) for SUMA and WEAU, days following screening (DFS) for CH40. Subject CH40 reported that symptom onset occurred two days before screening (JoAnn Kuruc, personal communication). A concurrent sample from WEAU's infecting partner RIER0489 (RIER) was also available for sequencing. RIER had end-stage HIV-1 infection at this time and died of AIDS several months later.

Tables S1 and S2 summarize previously published viral load, immunological responses and experimental validation of escape mutations, and epitope sequence variation patterns determined through conventional sequencing, as well as some additional historical data that has not been previously published and some newly determined viral load data based on assays of stored samples. Much of the key information was extracted from earlier studies [4], [5], [11], [9], [12] which provided the context for this ultra-deep 454 sequencing study. Immunologically important events derived from this 454 study data and analysis are integrated into the basic timeline for each subject and provided in Table S1.

### Primer design algorithm

The 454 primers were designed using a 3-step procedure. In step 1, a preexisting alignment covering the region of interest was considered and conserved primer locations were selected based on alignment positional entropy. We selected highly conserved regions within the patient that encompassed the four epitopes of interest, as well as the V3 loop in each subject, and that were an appropriate distance apart given the read-length limits of the 454 sequencing technology employed. In step 2, unique 454 identification-tags were generated. In step 3, the whole primer constructs were tested for potential dimer formation. The whole procedure has been automated (code available on request from Thomas Leitner, tkl@lanl.gov).

Step 1 considers an alignment of length *L*, which includes the region of interest (*ROI*). The user specifies the primer length (*P*) and the level of variation (*V*) that should be included in polymorphic sites of the alignment, expressed as a Hamming distance. Given a maximum expected read length *M* (∼250 bp for the Roche 454 FLX protocols and kits), *L* should be longer than *ROI*, such that *L>M*, *L*≥*ROI*+2*P*, and *M*≥*ROI*+*P*; therefore, the final read length will include at least (from 5′ to 3′) one complete primer with identification-tag and the full region of interest. The Shannon entropy for all possible *P* long primers is calculated, and low-entropy primers are identified to the left (5′) and right (3′) of *ROI*. Finally, each primer is expressed as a consensus sequence with degenerated nucleotide states to level *V*. At the 3′-end of each primer, a conserved (at level *V*) G or C was enforced. (Note: Levels of variation in the pre-existing SGA sequences were high enough in the V3 region that we could not always encompass the full V3 loop, and so sequences from this region were obtained that were slightly offset, and not centered on the loop.)

Step 2 creates a set of *N* tags of length *T*, with a controlled minimum edit distance (the Levenshtein distance [39] between tags. Known 454 sequencing problems are taken into account, so that no di-nucleotides are included in the selected tags or at the junctions with specific amplification primers (from step 1) and given 454 sequencing adaptors.

Step 3 calculates potential dimer formation such that no more than *H* potential bonds are formed anywhere between homo- and hetero-dimers of matching primers (including the specific primer from step 1, tag and 454 adaptors). The melting temperature *T_m_* is estimated according the nearest-neighbor model [40].

### Sample preparation and PCR amplification

To allow quantitative assessment of the viral population, both cDNA generation from viral RNA and subsequent PCR reactions used high template volume, low cycle numbers, and multiple replicates that were subsequently pooled for sequencing (Fig. S5). For each sample, RNA was isolated from a pellet containing at least 200,000 viral RNA copies using EZ-1 viral RNA kit (Qiagen). cDNA was synthesized using superscript III reverse transcriptase (Invitrogen) in 5 replicates with the antisense primer 1.R3.B3R (5′-ACTACTTGAAGCACTCAAGGCAAGCTTTATTG-3′; nt 9642-9611 HXB2).

The cDNA was immediately subjected to nested PCR amplification using one of two high-fidelity DNA polymerases [Phusion High-Fidelity DNA Polymerase (New England Biolabs), or Platinum Taq DNA Polymerase High Fidelity (Invitrogen); the latter amplified more reliably]. For each time point, 96 replicate PCR reactions (40 μl each) were performed with 5 μl cDNA, using forward primer BKB3F2 (5′ CGGGTTTATTACAGGGACAGCAG 3′; nt 4899–4921 HXB2) and the same reverse primer used for cDNA synthesis (1.R3.B3R). All 96 first round reactions for each time point were pooled and used as template for three inner PCR reactions. Each inner PCR reaction was performed with 32 replicates (40 μl each) using 5 μl of pooled first round template, and specific primers that incorporated a 4 base identification tag as well as a 19 base 454 adaptor sequence (Table S9). For our first 454 run, agarose gel-run PCR amplicons were visualized with ethidium bromide and ultraviolet light; for the following 3 runs, we replaced ethidium Br/UV with crystal violet/white light. Although we did not systematically test this due to the cost per experiment, avoiding DNA exposure to ultraviolet light seemed to substantially improve sequence quality. Excised amplicons were gel extracted (Qiagen) and subjected to 454 sequencing.

### 454 Sequencing

For our first run, a Bioanalyzer (Agilent, Santa Clara, California, USA) was used to determine sequence quality and quantity for 12 amplicon samples. All samples were diluted to 2×10^5^ molecules/μl and pooled. For emulsion PCR, 2.25 μl of the pooled sample was added to a reaction mix containing 450,000 capture beads per reaction. This ratio (1 copy per bead) we assumed would favor getting a single DNA molecule per capture bead in the emulsified reaction, and was the standard we had previously used for shotgun sequencing large genomes. The resulting products were recovered by breaking the emulsions and then enriching for beads containing amplified products. Approximately 400,000 enriched DNA beads from each reaction were deposited into one of 2 regions of a 70×75mm Pico Titer Plate and run on the Genome Sequencer FLX (Roche, Basel, Switzerland).

Yields were highly variable between samples, although the input stoichiometry had predicted they should be equivalent. We therefore did a titration run on multiple amplicons to determine the optimal ratio of DNA to beads. An input ratio of 9 copies per bead enhanced yields from some of the samples that had poor initial yields, suggesting that annealing to the beads was imperfect and that increasing the amplicon:capture bead ratio improved results. In subsequent runs, therefore, we diluted samples to 2×10^6^ molecules/μl and pooled them. For emulsion PCR, 2.025 μl of the pooled samples were added to a reaction mix containing 450,000 capture beads per reaction, and we proceeded as above. Sequencing of primers suggested they were of high quality, so this did not seem to be the reason increasing the amplicon-to-bead ratio improved the yield. We also compared the strategy of using a specified region on the plate for each amplicon, versus mixing the amplicons and using the whole plate and then subsequently relying on tags and primer to distinguish samples. We found the plates to be variable in terms of yield of sequence; no advantage was found in separating the different amplicons into different reactions, and running them on physically distinct subregions of each plate; such runs can in fact diminish the overall yield as part of each plate goes unused to provide delineated regions of separation. The costs of each run are so high we could not exhaustively compare strategies for enhancing yield. In summary, however, in our experience some of the low yield samples were markedly improved in a second run by increasing the amplicon-to-bead ratio.

Disproportionate replication can occur in 454 experiments during the emulsion PCR step, inflating the frequencies of some forms [41]. Despite this potential limitation on the precision of our frequency estimates, there was no overt enrichment of particular variants beyond expectation. The sequence read data for this study have been submitted to the NCBI Sequence Read Archive (http://www.ncbi.nlm.nih.gov/Traces/sra) under accession number SRA020793.

### Software based clean up

To define the nature of 454 errors, we used two controls. First, we included putatively homogeneous PCR products identical to the infective strain inferred from previous sequencing efforts [4], [12], to allow qualitative and quantitative assessment of sequencing error within context of the bases in the specific region studied. Second, we utilized the control DNA sequences included by the manufacturer and sequenced in parallel with our samples. Roughly 90% of all 454 sequencing errors we observed in the controls were insertions or deletions relative to the known control sequence; these indels were associated with homopolymer tracts. We modified these errors by deleting the extra base or adding a missing base; this strategy introduces a conservative bias (i.e., towards the transmitted sequence) and potentially underestimates the true diversity, but salvages otherwise useful sequences. This strategy may be more appropriate in the context of early infection where all sequences are very similar to the transmitted virus. Our approach differs from that of Quince and co-authors (PyroNoise, [42]) in being based entirely upon called bases rather than flowgram values; our rationale is that the normalized flowgram values are highly processed data, and appear to have distorted statistical properties. In addition to our sequence-based correction approach, we also applied the methods developed in our previous work with chronic infection samples [25] to detect and remove remaining error-containing sequences. Briefly, we trimmed the ends of the sequences relative to a reference strain, as the ends had the highest error rates; we then excluded sequences that had frameshifts relative to a reference HIV strain, that were too short, that contained long direct repeats, or that had a close pair of matched-length indels that created a short compensated frame-shift.

After the automated clean up, we were still left with a small number of problematic sequences that we excluded based on further analyses. Some sequences shared a particular stop codon, where the repeated mutation giving rise to the stop codon could be traced to a common 454 error pattern. These patterns resembled the indel patterns in homopolymer tracks, but rather than indels, they resulted in two apparently inverted bases. Since premature stop codons are likely to be lethal, we simply excluded all fragments with stop codons. Other sequences had a high frequency of indels relative to the transmitted virus; rather than attempting to repair such clearly damaged sequences, we excluded those with more than 5% positions of a sequence alignment containing an insertion or deletion when aligned to the sample consensus. Still other sequences were clearly hypermutated; these were marked so they could be considered for exclusion from subsequent analysis (p value <0.1 using the HyperMut tool, http://www.hiv.lanl.gov/content/sequence/HYPERMUT/hypermut.html). Finally, we removed several instances of sequences where the reverse and forward strand differed in a frequent base substitution, and that base substitution was embedded in a characteristic 454 error. Our clean-up software package now enables labeling of such potentially problematic sequences for future studies. The clean-up software package is available at (ftp site in progress and will be available prior to publication).

Data for entropy plots (Fig. 3) were calculated using the LANL HIV-DB “Entropy-Two” tool (http://www.hiv.lanl.gov/content/sequence/ENTROPY/entropy.html).

### Computational Methods for Sequence Analyses

We inferred phylogenies for each sample with PHYML (version 2.4.4, [43]) using the F81 DNA substitution model, and plotted the resulting trees with the APE package (version 2.4, [44]) in *R* (version 2.10.0, [45], http://www.r-project.org). *R* was also used for statistical analyses. The phylogenetic inference is based on a very small number of mutations embedded in very short sequences, with apparent convergence or recombination occurring repeatedly in the data, so the trees will inevitably be limited in reconstructing the true evolutionary history of the sequences. Nevertheless, the phylogenetic reconstructions give a clear sense of the patterns of variation over time, the emergence of escape, the complexity of the samples, and the associations of additional mutations with escape mutations. We rooted each tree with the most abundant variant from the earliest sample, i.e. the inferred transmitted virus, (except for the RIER/WEAU transmission pair tree, Fig. 4A, which was rooted among sequences from the donor, RIER). To provide a visual sense of the relative frequencies of different lineages, the branch widths are proportional to the logarithm of the cumulative frequency in the sample for all subtended (descendent) taxa. We added a small constant (0.0005) to zero-length terminal branch lengths to allow display of branch width. For CXCR4 predictions, we used the support-vector-machine and decision-tree usage-prediction strategies available at the WetCat web site (http://genomiac2.ucsd.edu:8080/wetcat/v3.html; [24]). Positional Shannon entropies were calculated using the tool available at the Los Alamos database: http://www.hiv.lanl.gov/content/sequence/ENTROPY/entropy.html).

## Supporting Information

Text S1 Additional methods and description of all supplementary figures and tables.
(0.19 MB PDF)

Table S1 Integration of previously reported and newly available basic clinical data regarding SUMA, WEAU, and CH40 with 454 sampling timeline.
(0.17 MB DOC)

Table S2 Conventional sequencing variants and previously available immunological data regarding escape.
(0.19 MB DOC)

Table S3 Aligned amino-acid sequences of the epitope regions with variant frequencies, organized by subtype, escape form, and time point.
(0.38 MB DOC)

Table S4 Modeling results for first time point samples for WEAU, CH40 and SUMA.
(0.08 MB DOC)

Table S5 Poisson compatibility within major escape lineages.
(0.09 MB DOC)

Table S6 Estimates of accumulation rates of dominant viral variants.
(0.05 MB DOC)

Table S7 Frequencies of not-transmitted B consensus amino acids.
(0.06 MB DOC)

Table S8 Summary of subtype consensus frequencies in 4 chronically infected subjects from an earlier study [supplement ref. 10, Tsibris et al.].
(0.05 MB DOC)

Table S9 Inner PCR/sequencing primers (forward, F; reverse, R).
(0.09 MB DOC)

Figure S1 Annotated example of format for Table S3, which includes aligned amino-acid sequences of the epitope regions with variant frequencies, organized by subtype, escape form, and time point. The subject ID, and count of the number of variants with a given protein sequence are shown. The epitope is in bold, and the array of secondary mutations that are found in conjunction with the N to K substitution are shown; the dominant escape forms have secondary mutations that are consistent with a Poisson distribution, with the exception of the overlapping epitope region in SUMA Tat (Table S5). Table S3 includes complete data for all 4 epitope regions.
(0.11 MB EPS)

Figure S2 Phylogenetic trees for ENV V3 DNA sequences, by time point. (A) WEAU, (B) CH40, and (C) SUMA. Branch widths are proportional to the log-ratio of abundance in the sample; trees are rooted by the most common sequence (the transmitted/founder virus); times are in days from symptoms (WEAU, SUMA) or from screening (CH40).
(1.87 MB EPS)

Figure S3 Phylogenetic trees of ENV V3 DNA sequences (all timepoint samples combined for each subject). (A) WEAU, (B) CH40,and (C) SUMA, pooled from all samples, with branch color indicating sample timepoint (blue, 1st timepoint; green, 2nd; red. 3rd). Branch widths are proportional to the log-ratio of abundance in the sample; trees are rooted by the most common sequence (the transmitted/founder virus); times are in days from symptoms (WEAU, SUMA) or from screening (CH40).
(2.12 MB EPS)

Figure S4 Distributions of accumulation rates of viral variants generated during acute infection. In the cases when the frequency of a variant was below the level of detection (i.e., less than 1 sequence per sample), we added the value of 1/N to the variant frequency at that time point. Equation 3 was used to estimate the rate of escape ! for every viral variant. The distribution of escape rates is very wide, with some variants escaping at negative rates (i.e., declining in frequency), and a very few having extremely rapid escape rates. A) WEAU Env AY9 epitope; B) CH40 Nef SR9 epitope; C) SUMA Rev QL9 epitope; D) SUMA Tat FY16 multi-epitope region.
(0.11 MB EPS)

Figure S5 Amplification protocol. The protocol was designed with the intent of reducing loss of diversity during PCR amplification by (1) limiting the number of cycles (2) using large amounts of template, and (3) using multiple small amplification reactions which were pooled for sequencing.
(0.77 MB TIF)
