# Supplementary material for: Transmission of Single HIV-1 Genomes and Dynamics of Early Immune Escape Revealed by Ultra-Deep Sequencing
Source: PLoS One. 2010 Aug 20;5(8):e12303. doi: 10.1371/journal.pone.0012303 (PMC2924888; doi:10.1371/journal.pone.0012303)
Supplement: Text S1 — Additional methods and description of all supplementary figures and tables. (0.19 MB PDF) [file pone.0012303.s001.pdf]

Supplementary Data
"Transmission of Single HIV-1 Genomes and Dynamics of Early Immune Escape Revealed by Ultra-deep Sequencing"

Will Fischer[1], Vitaly V. Ganusov[1,2,†], Elena E. Giorgi[1,3,†], Peter Hraber[1,†], Thomas Leitner[1,†], Brandon F. Keele[4,†], Cliff S. Han[1], Cheryl Gleasner[1], Lance Green[1], Chien-Chi Lo[1], Ambarish Nag[1], Timothy C. Wallstrom[1], Shuyi Wang[5], Andrew J. McMichael[6], Barton F. Haynes[7], Beatrice H. Hahn[5], Alan S. Perelson[1], Persephone Borrow[8], George M. Shaw[5], Tanmoy Bhattacharya[1,9], Bette T. Korber[1,9,*]

1 Theoretical Biology, Los Alamos National Laboratory, Los Alamos, NM 87505, USA

2 Department of Microbiology, University of Tennessee, Knoxville, TN 37996

3 Department of Mathematics and Statistics, University of Massachusetts, Amherst, MA 01002

4 SAIC-Frederick, National Cancer Institute, Frederick, MD 21702 USA

5 Department of Medicine, University of Alabama at Birmingham, AL 35294, USA

6 Weatherall Institute of Molecular Medicine, Oxford University, Oxford OX3 9DS, England, UK

7 Duke University Medical Center, Durham, North Carolina, 27710 USA

8 The Jenner Institute, University of Oxford, Compton, Berkshire RG20 7NN, England, UK

9 The Santa Fe Institute, Santa Fe, NM 87501, USA

∗ E-mail: btk@lanl.gov

† These authors contributed equally to this paper.

Contents

All supplementary figures and tables referenced below are supplied as separate files.

Section I. Basic supporting figures and tables

Table S1. Integration of previously reported and newly available basic clinical data regarding SUMA, WEAU, and CH40 with 454 sampling timeline.

Table S2. Conventional sequencing variants and previously available immunological data regarding escape.

Table S3. Aligned amino-acid sequences of the epitope regions with variant frequencies, organized by subtype, escape form, and time point.

Figure S1. Annotated example of format for Table S3.

Table S4. Modeling results for first time point samples for WEAU, CH40 and SUMA.

Table S5. Poisson compatibility within major escape lineages.

Figure S2. Phylogenetic trees for ENV V3 DNA sequences, by time point.

Figure S3. Phylogenetic trees of ENV V3 DNA sequences (all timepoint samples combined for each subject).

Section II. Immune Escape Dynamics

Table S6. Estimates of accumulation rates of dominant viral variants.

Figure S4. Distributions of accumulation rates of viral variants generated during acute infection.

Section III. Subtype B consensus: reversion and escape

Table S7. Alignments and frequencies of not-transmitted B consensus amino acids among all sequences, V3 and epitope regions.

Table S8. Summary of subtype consensus frequencies in 4 chronically infected subjects from an earlier study.

Section IV. Figures and Tables related to experimental methods

Table S9. Inner PCR primers.

Figure S5. Amplification protocol.

Section I Basic supporting figures and tables

These tables (discussed in the main text) provide a comprehensive summary of immunological data on these patients. New data generated in the course of this study were compiled and integrated with data from previous publications [1–5].

Section II Immune Escape Dynamics

We applied previously developed methods to quantify rates of viral escape from the CTL response [6–8] to the 454 data. The patterns and rates of viral escape vary between different epitope regions and different patients (see main text and Fig. S4). In particular, in two patients, escape variants that were at high frequency at the intermediate time point became substituted later by other variants (Fig. 5, main text).

For all viral variants, we estimated the rate of accumulation in the viral population during the early and late time points (Fig. S4). (The rate of accumulation, ε, is the coefficient of time in the exponential growth equation $N = N_0 \times e^{\varepsilon t}$. The doubling time of a variant is $T_2 = ln(2)/\varepsilon$.) The estimated rates are highly variable. In general, most mutations either accumulate slowly or disappear in the population (negative accumulation rates), while a few mutations accumulate rather quickly, and the distribution of accumulation rates changes over the time period studied for different epitopes.

Given our estimates on the rate of accumulation of the dominant viral escapes, we also estimated the time when the selection to avoid the CTL response started (see below for details and the assumptions). The day when the initial frequency of the selected variant was predicted to be 5×10^{-5} (i.e., between 10^{-5} and 10^{-4}) was used as the estimate of the day (range of days) on which selection was initiated. By this measure, we obtained the following estimates for the start of selection (in days relative to day 0 in our data): WEAU Env AY9, 4.9 (1.3 to 6.5; estimated escape rate $\varepsilon = 0.44$ day^{-1}; doubling time $T_2 = 1.58$ days); CH40 Nef SR9, -2.9 (-7.8 to -0.8; $\varepsilon = 0.32$ day^{-1}; doubling time $T_2 = 2.17$ days); SUMA Tat FY16, 10.2 (2.8 to 13.3; $\varepsilon = 0.22$ day^{-1}; doubling time $T_2 = 3.15$ days); SUMA Rev QR9, 17.9 (14 to 19.5; $\varepsilon = 0.42$ day^{-1}; doubling time $T_2 = 1.65$ days). Table S6 shows the accumulation or loss rates of various escape forms. To calculate the 95% confidence intervals (CIs) for the estimated rate of accumulation of different escape variants we used a bootstrap approach to regenerate escape data [9]. For a given time point, where there are total N sequences and m of these are of a particular mutant (i.e., $m < N$ and the frequency of the mutant in the population is $p = m/N$), we generated a sample frequency

of the mutant as $B_N(m/N)/N$ where $B_N(p)$ is a binomial distribution for N trials with the success rate per trial $p = m/N$. Resampling was repeated independently 1000 times for each escape variant.

The model for the dynamics of escape of a virus from a single CTL response has been described in detail previously [6–8]. Under several assumptions, change in the frequency of the escape variant $f(t)$ is given by the formula

$$f(t) = \frac{f_0}{f_0 + (1 - f_0)\mathrm{e}^{-\varepsilon t}}, \tag{1}$$

where f_0 is the frequency of the escape mutant in the population at some time which we arbitrarily call $t = 0$ and ε is the rate of accumulation of the escape variant in the population. From Equation 1 it follows that the ratio of a given escape variant to all other variants in the population:

$$z(t) = f(t)/(1 - f(t)), \tag{2}$$

changes exponentially over time:

$$z(t) = z_0 \mathrm{e}^{\varepsilon t}, \tag{3}$$

where $z(t)$ and z_0 are the ratio of the frequency of the escape mutant to the frequency of the wild type virus in the population at some time t and at time $t = 0$, respectively.

Since for many viral variants we have precise estimates of the rate of escape from the CTL response, we can calculate the approximate time when the selection started (i.e. when the CTL response began killing cells infected with the transmitted/founder virus). For that we rewrite Equation 1 by letting T be the time when the selection started with f_T being the frequency of the viral variant at this time point:

$$f(t) = \frac{f_T}{f_T + (1 - f_T)\mathrm{e}^{-\varepsilon(t-T)}}, \tag{4}$$

Note that this model assumes that selection is constant during the whole period of selection. Equation 4 can be then fit to the data on the dynamics of a given escape variant (the dominant variant by the third time point in our analysis) to estimate ε and T given that f_T is fixed (in our estimates we let $f_T = 10^{-5}, 5 \times 10^{-5}, 10^{-4}$). In those cases when there is evidence of change of the escape rate over time (i.e., ratio z changes bi-exponentially; e.g., CH40 and WEAU), we fix the rate of escape to its maximal

value ε and estimate only T. The estimated rates of escape are reported together with the range of times at which the selection pressure is estimated to have started. Because we generally obtain the upper bound average estimate on the escape rate (except for SUMA Tat where the upper bound estimate is infinity, results not shown), our estimated time of the start of the selective pressure is an upper bound. Assuming an increase in the selective pressure over time would reduce the estimated time of the start of the selective pressure.

Section III Subtype B consensus: reversion and escape

As discussed in the main text, there were a number of positions where the transmitted virus did not match the B subtype consensus in these three subjects. These are indicated in the alignment in Table S3. Fig. 8 summarizes the B consensus amino acid frequencies in each patient at each time point in the epitope regions, and Table S7 provides a more explicit breakdown of the data in the epitopes and V3 regions. Within epitopes (shown in red) there was selection for the B consensus in 6/8 positions. 4/6 of these B consensus substitutions diminished in frequency over time. There was no selection for B consensus amino acids outside of the epitopes (blue), hence no evidence for rapid reversion on this time scale. In contrast, RIER, with a chronic infection, often carried common B consensus variants (Fig. 8 in green, and Table S7 A and B): in 3 positions, over 30% of the sequences matched the consensus, at 3, the consensus was found at low but clearly replicating circulating levels (1-15%). This left only one position with undetectable levels of B consensus amino acids (green). In 4 chronic-infection patients in our earlier 454 study [10], consensus amino acid frequencies similar to those in RIER were present (See Table S8). Thus during chronic HIV-1 infection, the B consensus amino acids are generally present and replicating even when they are not the most common form in an individual.

Section IV Figures and Tables related to experimental methods

Table S9 presents the inner PCR primers used for specific fragment amplification following half-genome RT-PCR, with multiplex sequence tags.

Figure S5 summarizes the amplification protocol, which was designed to maintain diversity in the amplified DNA pools used as sequencing template.

References

1. Borrow P, Lewicki H, Hahn BH, Shaw GM, Oldstone MB (1994) Virus-specific CD8+ cytotoxic T-lymphocyte activity associated with control of viremia in primary human immunodeficiency virus type 1 infection. J Virol 68: 6103-10.

2. Goonetilleke N, Liu MK, Salazar-Gonzalez JF, Ferrari G, Giorgi E, et al. (2009) The first T cell response to transmitted/founder virus contributes to the control of acute viremia in HIV-1 infection. J Exp Med 206: 1253-72.

3. Jones NA, Wei X, Flower DR, Wong M, Michor F, et al. (2004) Determinants of human immunodeficiency virus type 1 escape from the primary CD8+ cytotoxic T lymphocyte response. J Exp Med 200: 1243-56.

4. Keele BF, Giorgi EE, Salazar-Gonzalez JF, Decker JM, Pham KT, et al. (2008) Identification and characterization of transmitted and early founder virus envelopes in primary HIV-1 infection. Proc Natl Acad Sci U S A 105: 7552-7.

5. Borrow P, Lewicki H, Wei X, Horwitz MS, Peffer N, et al. (1997) Antiviral pressure exerted by HIV-1-specific cytotoxic T lymphocytes (CTLs) during primary infection demonstrated by rapid selection of CTL escape virus. Nat Med 3: 205-11.

6. Asquith B, Edwards CT, Lipsitch M, McLean AR (2006) Inefficient cytotoxic T lymphocyte-mediated killing of HIV-1-infected cells in vivo. PLoS Biol 4: e90.

7. Fernandez CS, Stratov I, De Rose R, Walsh K, Dale CJ, et al. (2005) Rapid viral escape at an immunodominant simian-human immunodeficiency virus cytotoxic T-lymphocyte epitope exacts a dramatic fitness cost. J Virol 79: 5721-31.

8. Ganusov VV, De Boer RJ (2006) Estimating costs and benefits of CTL escape mutations in SIV/HIV infection. PLoS Comput Biol 2: e24.

9. Efron B, Tibshirani R (1993) An introduction to the Bootstrap, volume 57 of *Monographs on Statistics and Applied Probability*. Chapman and Hall/CRC Press.

10. Tsibris AM, Korber B, Arnaout R, Russ C, Lo CC, et al. (2009) Quantitative deep sequencing reveals dynamic HIV-1 escape and large population shifts during CCR5 antagonist therapy in vivo. PLoS One 4: e5683.

11. Wood N, Bhattacharya T, Keele BF, Giorgi E, Liu M, et al. (2009) HIV evolution in early infection: selection pressures, patterns of insertion and deletion, and the impact of APOBEC. PLoS Pathog 5: e1000414.

12. Lee HY, Giorgi EE, Keele BF, Gaschen B, Athreya GS, et al. (2009) Modeling sequence evolution in acute HIV-1 infection. J Theor Biol 261: 341-60.

13. Drummond AJ, Rambaut A (2007) BEAST: Bayesian evolutionary analysis by sampling trees. BMC Evol Biol 7: 214.

Supplementary Figure Legends

Figure S1. Annotated example of format for Table S3, which includes aligned amino-acid sequences of the epitope regions with variant frequencies, organized by subtype, escape form, and time point. The subject ID, and count of the number of variants with a given protein sequence are shown. The epitope is in bold, and the array of secondary mutations that are found in conjunction with the N to K substitution are shown; the dominant escape forms have secondary mutations that are consistent with a Poisson distribution, with the exception of the overlapping epitope region in SUMA Tat (Table S5). Table S3 includes complete data for all 4 epitope regions.

Figure S2. Phylogenetic trees for ENV V3 DNA sequences, by time point. (A) WEAU, (B) CH40, and (C) SUMA. Branch widths are proportional to the log-ratio of abundance in the sample; trees are rooted by the most common sequence (the transmitted/founder virus); times are in days from symptoms (WEAU, SUMA) or from screening (CH40).

Figure S3. Phylogenetic trees of ENV V3 DNA sequences (all timepoint samples combined for each subject). (A) WEAU, (B) CH40,and (C) SUMA, pooled from all samples, with branch color indicating sample timepoint (blue, 1st timepoint; green, 2nd; red. 3rd). Branch widths are proportional to the log-ratio of abundance in the sample; trees are rooted by the most common sequence (the transmitted/founder virus); times are in days from symptoms (WEAU, SUMA) or from screening (CH40).

Figure S4. Distributions of accumulation rates of viral variants generated during acute infection. In the cases when the frequency of a variant was below the level of detection (i.e., less than 1 sequence per sample), we added the value of $1/N$ to the variant frequency at that time point. Equation 3 was used to estimate the rate of escape ε for every viral variant. The distribution of escape rates is very wide, with some variants escaping at negative rates (i.e., declining in frequency), and a very few having extremely rapid escape rates. A) WEAU Env AY9 epitope; B) CH40 Nef SR9 epitope; C) SUMA Rev QL9 epitope; D) SUMA Tat FY16 multi-epitope region.

Figure S5. Amplification protocol. The protocol was designed with the intent of reducing loss of diversity during PCR amplification by (1) limiting the number of cycles (2) using large amounts of template, and (3) using multiple small amplification reactions which were pooled for sequencing.
