# Supplementary material for: Transmission of Single HIV-1 Genomes and Dynamics of Early Immune Escape Revealed by Ultra-Deep Sequencing
Source: PLoS One. 2010 Aug 20;5(8):e12303. doi: 10.1371/journal.pone.0012303 (PMC2924888; doi:10.1371/journal.pone.0012303)
Supplement: Table S1 — Integration of previously reported and newly available basic clinical data regarding SUMA, WEAU, and CH40 with 454 sampling timeline. (0.17 MB DOC) [file pone.0012303.s002.doc]

Table S1. Integration of previously reported and newly available basic clinical data regarding SUMA, WEAU, and CH40 with 454 sampling timeline (Supplement refs. [1–5] plus new information).

| **Patient** | **Risk factor** | **HLA** | **Virological course** |
| --- | --- | --- | --- |
| **SUMA0874 (SUMA)** | **MSM** | **A*1103, A*2402,**  **B*1402, B*1501,**  **Cw*0802, Cw*1203** | Low viral loads for the first year, gradually increasing; maintained high CD4 (722 cells/mm^3^ at 7 years of follow up**;** the subject subsequently moved and was lost to follow up.) |

| **Epitope** | **Presenting HLA** | **Sequence** | **Notes** |
| --- | --- | --- | --- |
| **Tat MY9** | **B*1501** | **MTKGLGISY** | Immunodominant in acute infection [3] |
| **Tat FK10** | **A*1103**  **(putative)** | **FHCQVCFMTK** | Low level response in acute infection [3] |
| **Tat VI10** | **A*2402**  **(putative)** | **VCFMTKGLGI** | Low level response in acute infection [3] |
| **Rev QL9** | **B*1402**  **(putative)** | **QRQRQIQSL** | (ref. *2, Fig. S3*) |

| **Date** | **DFOSx** | **VL** | **CD4** | **Sequences** | **Timeline of events** |
| --- | --- | --- | --- | --- | --- |
| 08-May-91 | 0 |  |  |  | Onset of symptoms (acute HIV infection) |
| 12-May-91 | 4 |  |  | Conventional: Tat, Rev |  |
| 13-May-91 | 5 | 939,260 | 760 | **454: Tat, Rev, V3**; Conventional: Rev | Estimated to be 5-12 days from the founder, Fiebig stage II |
| 16-May-91 | **8** | **1,485,040** | 853 | Conventional: Tat | TAT MY9 is one of the initial immunodominant responses [3]; peak virema. |
| 18-May-91 | 10 |  |  |  | Selective pressure estimated to have initiated in Tat MY9 at d10 (range d2.8-13.3) based on rates of mutation accumulation; the rate of accumulation was slow, 0.22 per day (see Section IV for methods). |
| 21-May-91 | 13 | 701,640 |  |  |  |
| 23-May-91 | 15 | 563,997 |  |  |  |
|  | 18 |  |  |  | Selective pressure estimated to have initiated in Rev QL9 at d17.9 (range d14-d19.5) based on rates of mutation accumulation (see Section IV for methods). |
| 28-May-91 | 20 | 106,543 |  | **454: Tat, Rev, V3**; Conventional: Tat, Rev | Neither Tat and Rev sequences show evidence for selection, in that the within-epitope amino acid substitutions are at low levels and are not enriched (Fisher’s exact p = 0.95 and 0.98, respectively) |
| 30-May-91 | 22 | 11,240 |  |  |  |
| 04-Jun-91 | 27 | 87,251 |  |  |  |
| 06-Jun-91 | 29 | 20,560 |  |  |  |
| 11-Jun-91 | 34 | 14,780 |  | Conventional: Rev |  |
| 18-Jun-91 | 41 | 27,460 |  | **454: Tat, Rev, V3**; Conventional: Tat | Clear evidence of selection: transmitted epitope is at 94% in Tat, 5% in Rev |
| 11-Jul-91 | 64 |  | 1108 |  |  |
| 16-Jul-91 | 69 | 6,320 |  | Conventional: Tat |  |
| 10-Feb-92 | 278 | 2,268 |  |  |  |
| 25-Feb-92 | 293 | 5,640 | 977 |  |  |
| 24-Apr-92 | 352 | 14,860 |  |  |  |
| 16-Jul-92 | 435 | 13,600 | 952 |  |  |
| 02-Sep-92 | 483 | 13,105 | 1045 |  |  |
| 22-Feb-93 | 656 | 54,000 | 855 |  |  |

| **Patient** | | **Risk factor** | **HLA** | **Virological course** |
| --- | --- | --- | --- | --- |
| **WEAU0575**  **(WEAU)**  **Partner: RIER0489 (RIER)** | | **MSM** | **A*2902, A*0101, B*0801, B*4403, Cw*0701, Cw*1601** | WEAU had high persistent viral loads and rapid decline in CD4 T cells by 2 years from infection; clinical AIDS (CD4+ count < 200) at d275; death at approximately d1600 (4 years, 5 months from infection) |
|  | |  |  |  |
| **Epitope** | **Presenting HLA** | | **Sequence** | **Notes** |
| **ENV AY9** | **B*4403** | | **AENLWVTVY** | AY9 is optimal for one T cell clone, AY10, AENLWVTVYY for another |

| **Date** | **DFOSx** | **VL** | **CD4** | **Sequences** | **Timeline of events** |
| --- | --- | --- | --- | --- | --- |
| 15-May-90 | 0 |  |  |  | DFOSx d0 was 20 days after a single sexual encounter with RIER, partner with AIDS [5]. |
|  | 5 |  |  |  | Selective pressure estimated to have initiated in Env AY9 at d4.9 (range d1.3-d6.5) |
| 25-May-90 | 10 | 93,600 |  | **454: Env, V3** | Within-epitope amino acid substitutions are highly enriched (Fisher’s exact p = 1.6 x 10^-8^, odds ratio 1.74 (95% CI 1.4-2.1), but the transmitted virus is still at 99%. (Note: VL measurement on stored serum, 18-Dec-2009; Roche COBAS assay) |
| 30-May-90 | 15 | 216,415 | 358 | Conventional | Fiebig stage II |
| 31-May-90 | 16 |  |  | Conventional | Initial immunodominant CTL response is measured [5] |
| 04-Jun-90 | 20 | 355,184 |  | **454: Env, V3** | Clear broad selection has initiated, transmitted epitope is at 86% |
| 07-Jun-90 | 23 | **355,360** |  | Conventional | Peak viremia |
| 11-Jun-90 | 27 | 146,816 | 748 |  |  |
| 14-Jun-90 | 30 |  |  | **454: Env, V3;**  Conventional | Transmitted epitope has declined to 38% |
| 18-Jun-90 | 34 | 100,877 | 568 |  |  |
| 24-Jun-90 | 40 |  | 972 |  |  |
| 28-Jun-90 | 44 | 34,737 |  | Conventional |  |
| 05-Jul-90 | 51 |  |  |  |  |
| 12-Jul-90 | 58 |  |  |  |  |
| 26-Jul-90 | 72 | 11,385 |  | Conventional |  |
| 08-Sep-90 | 86 |  |  |  |  |
| 28-Sep-90 | 136 | 17,322 |  | Conventional |  |
| 13-Dec-90 | 212 | 90,109 |  | Conventional |  |
| 14-Feb-91 | 275 |  | 197 |  | Clinical AIDS (CD4+ < 200) |
| 10-Jun-90 | 391 | 55,269 | 89 | Conventional |  |
| 06-Nov-91 | 540 | 94,474 | 14 |  |  |
| 03-May-92 | 660 | 61,667 |  |  |  |
| 25-Jun-92 | 772 | 55,900 | 30 |  |  |
| 14-Jul-92 | 791 | 52,745 |  |  |  |
| 09-Feb-93 | 1001 |  |  |  |  |
| 26-Jul-93 | 1168 |  | 5 |  |  |
| Oct-94 | 1602 |  |  |  | Death |

| **Patient** | **Risk factor** | **HLA** | **Virological course** |
| --- | --- | --- | --- |
| **CHAVI**  **700-01-004-0 (CH40)** | **MSM** | **A*0201 A*3101**  **B*4001 B*4402**  **Cw*0302 Cw*0501** | Enrolled in CHAVI protocol 001. Viral set point based on data from Oct 30, 2006-April 28, 2008: 13,224 copies per/ml |

| **Epitope** | | **Presenting HLA** | | | **Sequence** | | **Notes** |
| --- | --- | --- | --- | --- | --- | --- | --- |
| **NEF SR9** | | **A*2402**  **(putative)** | | | **SLAFRHVAR** | | One of the dominant responses at d16 post-screening, but rapid escape resulted in response dropping to undetectable levels [2] |
|  | |  | | |  | |  |
| **Date** | **DFS** | | **VL** | **CD4** | | **Sequences** | **Timeline of events** |
|  | -5 | |  |  | |  | Selective pressure initiated in Nef SR9 at d -4.9 (range days -9.8 to -2.8) |
| 09-Jul-06 | -2 | |  |  | |  | Self-reported onset of symptoms of acute HIV infection |
| 11-Jul-06 | 0 | | **2,197,248** |  | | **454: Nef, V3** | Screening: Fiebig stage II, no evidence for selection in SR9, in that the within-epitope amino acid substitutions are not enriched (Fisher’s exact p = 0.84), EIA negative |
| 27-Jul-06 | 16 | | 298,026 | 929 | | **454: Nef, V3**  Conventional | Enrollment: Strongest early CTL response, escape was well underway, transmitted epitope declined to 48%, Fiebig stage V |
| 27-Jul-06 | 16 | | 213,333 |  | |  | Duplicate VL test, Denny Lab |
| 25-Aug-06 | 45 | | 89,156 | - | | **454: Nef, V3**  Conventional | Transmitted virus declined further to 0.4%, ELISpot for SR9 declined to undetectable |
| 30-Oct-06 | 111 | | 17,587 | 1017 | | Conventional |  |
| 08-Jan-07 | 181 | | 29,453 | 986 | |  |  |
| 20-Apr-07 | 283 | | 7,580 | 994 | |  |  |
| 01-Aug-07 | 181 | | 29,453 | 986 | | Conventional |  |
| 27-Aug-07 | 412 | | 33,918 | 1396 | |  |  |
| 22-Oct-07 | 468 | | 8,125 | 1069 | |  |  |
| 14-Jan-08 | 552 | | 8,865 | 882 | |  |  |
| 21-Apr-08 | 650 | | 7,594 | 941 | |  |  |
| 07-Jul-08 | 727 | | 2,788 | 972 | |  |  |

Table S1. Integration of previously reported and newly available basic clinical data regarding SUMA, WEAU, and CH40 with 454 sampling timeline. Patient viral load (VL) (copies/ml), days following on set of symptoms (DFOSx) or days following screening (DFS), CD4 T cell lymphocytes/ml, samples used for 454 or conventional sequencing, and comments briefly describing key immunological events (timeline of events) are provided. The highest viral load is noted in bold. In the time line of events, a Fisher’s exact test based on 454 sequences from the time point prior to overt selection is presented. This statistic showed that amino acid substitutions were already highly over-represented within the epitope in the first WEAU sample (10 DFOSx), indicating selection was already well underway. In contrast, the amino acid substitutions were evenly distributed inside and outside of the epitopes in SUMA Rev and Tat at day 20, our second time point, and at screening, day 0, in CH40, the first time point. The Fisher’s exact test was based on counting both the number of amino acid changes and the number of unchanging amino acids either within or outside the defined epitope regions at the time point of interest. Also provided is an estimated time from beginning of selection, based on the rate of acquisition of escape mutations, described in Section II. This estimate can plausibly be a few generations prior to the observation of enriched mutations within the epitope. A “putative” HLA is noted based on anchors residues from the subject’s HLA molecules being present within a known epitope, when the HLA presenting molecule was not experimentally determined.
