# Supplementary material for: Transmission of Single HIV-1 Genomes and Dynamics of Early Immune Escape Revealed by Ultra-Deep Sequencing
Source: PLoS One. 2010 Aug 20;5(8):e12303. doi: 10.1371/journal.pone.0012303 (PMC2924888; doi:10.1371/journal.pone.0012303)
Supplement: Table S2 — Conventional sequencing variants and previously available immunological data regarding escape. (0.19 MB DOC) [file pone.0012303.s003.doc]

| Table S2. Conventional sequencing variants and previously available immunological data regarding escape (Supplementary refs. [1-5] and, for SUMA FY10, Persephone Borrow unpublished data). | | | | | |
| --- | --- | --- | --- | --- | --- |
| **SUMA Tat 3 epitopes by conventional sequencing, using SGA** | | | | | |
| **Day** | **Epitopes** | **N Variant** | **N Total** | **Fraction** | **Immunological Results, ^51^Cr release** |
| 4 | FHCQVCFMTKGLGISY | 11 | 13 | 0.85 | transmitted |
|  | FHCQVCF**T**TKGLGISY | 2 | 13 | 0.15 | FY10 susceptible, FK10 escape, VI10 escape |
|  |  |  |  |  |  |
| 8 | FHCQVCFMTKGLGISY | 11 | 11 | 1 | transmitted |
|  |  |  |  |  |  |
| 20 | FHCQVCFMTKGLGISY | 19 | 20 | 0.95 | transmitted |
| 20 | FHCQVCF**T**T**E**GLGISY | 1 | 20 | 0.05 |  |
|  |  |  |  |  |  |
| 41 | FHCQVCFMTKGLGISY | 14 | 14 | 1 | transmitted |
|  |  |  |  |  |  |
| 69 | **K**HCQVCFM**L**KGLGISY | 14 | 14 | 1 | FY10: mutant peptide is recognized in a ^51^Cr release assay, but escape is conferred by processing; FK10 escape, VI10 escape |
|  |  |  |  |  |  |
| **SUMA Rev QL9 epitope by conventional sequencing, using SGA** | | | | | |
| **Day** | **Epitope** | **N Variant** | **N Total** | **Fraction** | **Immunological Results, ELISpot** |
| 4 | QRQRQIQSLS | 26 | 26 | 1 | transmitted |
|  |  |  |  |  |  |
| 5 | QRQRQIQSLS | 41 | 41 | 1 | transmitted |
|  |  |  |  |  |  |
| 13 | QRQRQIQSLS | 31 | 31 | 1 | transmitted |
|  |  |  |  |  |  |
| 20 | QRQRQIQSLS | 23 | 24 | 0.96 | transmitted |
| 20 | QRQR**H**IQSLS | 1 | 24 | 0.04 | escape |
|  |  |  |  |  |  |
| 34 | QRQRQIQSLS | 6 | 28 | 0.21 | transmitted |
| 34 | Q**K**QRQIQSLS | 2 | 28 | 0.07 | diminished susceptibility |
| 34 | QRQRQI**R**SLS | 6 | 28 | 0.21 | escape |
| 34 | QRQRQIQ**L**LS | 4 | 28 | 0.14 | escape |
| 34 | QRQRQIQS**I**S | 10 | 28 | 0.36 | diminished susceptibility |
|  |  |  |  |  |  |
| **WEAU Env AY9 epitope by conventional sequencing, using SGA** | | | | | |
| **Day** | **Epitopes** | **N Variant** | **N Total** | **Fraction** | **Immunological Results, ^51^Cr release** |
| 15 | AENLWVTVY | 39 | 42 | 0.93 | transmitted |
| 15 | **T**ENLWVTVY | 2 | 42 | 0.05 | sensitive |
| 15 | **V**ENLWVTVY | 1 | 42 | 0.02 |  |
|  |  |  |  |  |  |
| 16 | AENLWVTVY | 4 | 4 | 1 | transmitted |
|  |  |  |  |  |  |
| 23 | AENLWVTVY | 37 | 44 | 0.84 | transmitted |
| 23 | AE**K**LWVTVY | 4 | 44 | 0.09 | escape |
| 23 | **V**ENLWVTVY | 1 | 44 | 0.02 |  |
| 23 | A**K**NLWVTVY | 1 | 44 | 0.02 | escape |
| 23 | AEN**S**WVTVY | 1 | 44 | 0.02 |  |
|  |  |  |  |  |  |
| 30 | A**G**NLWVTVY | 3 | 11 | 0.27 | escape |
| 30 | AENLWVTVY | 2 | 11 | 0.18 | transmitted |
| 30 | AE**K**LWVTVY | 2 | 11 | 0.18 | escape |
| 30 | **T**ENLWVTVY | 1 | 11 | 0.09 | sensitive |
| 30 | **V**EN**S**WVTVY | 1 | 11 | 0.09 | escape |
|  |  |  |  |  |  |
| 44 | A**G**NLWVTVY | 6 | 30 | 0.2 | escape |
| 44 | **T**ENLWVTVY | 5 | 30 | 0.17 | sensitive |
| 44 | **V**ENLWVTVY | 5 | 30 | 0.17 |  |
| 44 | AE**K**LWVTVY | 5 | 30 | 0.17 | escape |
| 44 | A**K**NLWVTVY | 3 | 30 | 0.1 | escape |
| 44 | AE**D**LWVTVY | 2 | 30 | 0.07 |  |
| 44 | AE**S**LWVTVY | 2 | 30 | 0.07 |  |
| 44 | AENLWVTVY | 1 | 30 | 0.03 | transmitted |
| 44 | AE**T**LWVTVY | 1 | 30 | 0.03 |  |
|  |  |  |  |  |  |
| 72 | A**G**NLWVTVY | 3 | 9 | 0.33 | escape |
| 72 | AENLWVTVY | 1 | 9 | 0.11 | transmitted |
| 72 | **T**ENLWVTVY | 1 | 9 | 0.11 | sensitive |
| 72 | A**K**NLWVTVY | 1 | 9 | 0.11 | escape |
| 72 | A**A**NLWVT**A**Y | 1 | 9 | 0.11 | escape |
| 72 | **T**ENLWVT**I**Y | 2 | 9 | 0.22 | sensitive |
|  |  |  |  |  |  |
| 136 | A**G**NLWVTVY | 12 | 15 | 0.8 | escape |
| 136 | A**A**NLWVTVY | 3 | 15 | 0.2 | escape |
|  |  |  |  |  |  |
| 212 | A**G**NLWVTVY | 18 | 27 | 0.67 | escape |
| 212 | **T**ENLWVTVY | 7 | 27 | 0.26 | sensitive |
| 212 | A**K**NLWVTVY | 2 | 27 | 0.07 | escape |
|  |  |  |  |  |  |
| 391 | **T**E**K**LWVTVY | 2 | 2 | 1 | escape |
|  |  |  |  |  |  |
| **CH40 Nef SR9 epitope by conventional sequencing** | | | | | |
| **Day** | **Epitope** | **N Variant** | **N Total** | **Fraction** | **Immunological Results, ELISpot** |
| 15 | SSLAFRHVAR | 39 | 42 | 0.93 | transmitted |
| 16 | SSLAF**H**HVAR | 3 | 9 | 0.33 |  |
| 16 | S**R**LAFRHVAR | 2 | 9 | 0.22 |  |
|  |  |  |  |  |  |
| 45 | SSLAFRHVA**Q** | 11 | 14 | 0.79 | escape |
| 45 | S**N**LAFRHVAR | 2 | 14 | 0.14 | escape |
| 45 | SSLAF**H**HVAR | 1 | 14 | 0.07 |  |
|  |  |  |  |  |  |
| 111 | SSLAF**H**HVAR | 4 | 9 | 0.44 |  |
| 111 | SSLAFRHVA**Q** | 4 | 9 | 0.44 | escape |
| 111 | S**N**LAFRHVAR | 1 | 9 | 0.11 | escape |
|  |  |  |  |  |  |
| 181 | SSLAFRHVA**Q** | 7 | 7 | 1 | escape |
|  |  |  |  |  |  |
| 412 | SSLAFRHVA**Q** | 7 | 12 | 0.58 | escape |
| 412 | S**N**LAFRHVAR | 5 | 12 | 0.42 | escape |

Table S2. Previously published epitope sequence variation over time and a summary of available immune escape (supplement refs. [1-5]). In the Immunological results column ^51^Cr release means the peptide recognition was tested using a ^51^Cr release assay, while ELISpot means that peptide recognition was determined using an ELISpot Assay. Only one variant was experimentally determined to escape via antigen processing, FY10, noted in the SUMA Tat table; the other escape mutations were assessed by exogenously added peptides. If conventional sequences were based on single genome amplification (SGA) it is noted. Transmitted forms of the epitopes are highlighted in grey, and escape mutations are noted in bold.
