# Supplementary material for: Transmission of Single HIV-1 Genomes and Dynamics of Early Immune Escape Revealed by Ultra-Deep Sequencing
Source: PLoS One. 2010 Aug 20;5(8):e12303. doi: 10.1371/journal.pone.0012303 (PMC2924888; doi:10.1371/journal.pone.0012303)
Supplement: Table S3 — Aligned amino-acid sequences of the epitope regions with variant frequencies, organized by subtype, escape form, and time point. (0.38 MB DOC) [file pone.0012303.s004.doc]

**Table S3. Full amino acid alignments of each of the 4 epitope regions, organized by selected amino acid changes.**

All sequences are aligned to the transmitted form, dashes indicate identity to the transmitted form, periods are used to

offset the epitope boundaries. The places where the B subtype consensus differs from the transmitted virus are indicated.

Each sequences are repeated and have line representing every time point they are found in; thus a sequence found in all

three WEAU acute time points and the donor RIER would be included 4 times. The count and frequency of the sequence at

all time points are indicated on the right.

**____________________________________________________________________________________________________________________**

**WEAU**

**ID Day Rank tally __________________Sequence_________________________ ___________Tally______________ _________Frequency_____________**

Transmitted MLLGILMICSA.AENLWVTVY.YGVPVWKEATTTLFCASDAKAYDTEVHNV d10 d20 d30 RIER d10 d20 d30 RIER

B consensus ----M------.--K------.-----------------------------

Transmitted form:

WEAU d10 0001 21975 -----------.---------.----------------------------- 21975 9535 2153 6396 0.93718 0.80601 0.36430 0.33835

WEAU d20 0001 9535 -----------.---------.----------------------------- 21975 9535 2153 6396 0.93718 0.80601 0.36430 0.33835

WEAU d30 0001 2153 -----------.---------.----------------------------- 21975 9535 2153 6396 0.93718 0.80601 0.36430 0.33835

RIER --- 0001 6396 -----------.---------.----------------------------- 21975 9535 2153 6396 0.93718 0.80601 0.36430 0.33835

Second pre-escape lineage, likely to have arisen as a stochastic event early in infection:

WEAU d10 0002 792 -----------.---------.----------I------------------ 792 395 42 2 0.03378 0.03339 0.00711 0.00011

WEAU d10 0035 6 ---E-------.---------.----------I------------------ 6 0 0 0 0.00026 0.00000 0.00000 0.00000

WEAU d10 0044 5 -F---------.---------.----------I------------------ 5 0 0 0 0.00021 0.00000 0.00000 0.00000

WEAU d10 0055 4 -----------.---------.-E--------I------------------ 4 0 0 0 0.00017 0.00000 0.00000 0.00000

WEAU d10 0099 1 --------Y--.---------.----------I------------------ 1 0 0 0 0.00004 0.00000 0.00000 0.00000

WEAU d10 0111 1 -----------.---------.-----R----I------------------ 1 0 0 0 0.00004 0.00000 0.00000 0.00000

WEAU d10 0113 1 -----------.---------.----------I-----------------F 1 0 0 0 0.00004 0.00000 0.00000 0.00000

WEAU d10 0125 1 -------T---.---------.----------I------------------ 1 0 0 0 0.00004 0.00000 0.00000 0.00000

WEAU d10 0153 1 -----------.--------S.----------I------------------ 1 0 0 0 0.00004 0.00000 0.00000 0.00000

WEAU d10 0154 1 ----------V.---------.----------I------------------ 1 0 0 0 0.00004 0.00000 0.00000 0.00000

WEAU d20 0003 395 -----------.---------.----------I------------------ 792 395 42 2 0.03378 0.03339 0.00711 0.00011

WEAU d20 0096 1 -----------.---------.----------I------E----------- 0 1 0 0 0.00000 0.00008 0.00000 0.00000

WEAU d20 0137 1 --F--------.---------.----------I------------------ 0 1 0 0 0.00000 0.00008 0.00000 0.00000

WEAU d20 0163 1 -----S-----.---------.----------I------------------ 0 1 0 0 0.00000 0.00008 0.00000 0.00000

WEAU d20 0169 1 ------R----.---------.----------I------------------ 0 1 0 0 0.00000 0.00008 0.00000 0.00000

WEAU d20 0206 1 ---------C-.---------.----------I------------------ 0 1 0 0 0.00000 0.00008 0.00000 0.00000

WEAU d20 0212 1 -----------.---------.----------I---------I-------- 0 1 0 0 0.00000 0.00008 0.00000 0.00000

WEAU d30 0010 42 -----------.---------.----------I------------------ 792 395 42 2 0.03378 0.03339 0.00711 0.00011

WEAU d30 0071 1 -----------.---------.----------I--C--------------- 0 0 1 0 0.00000 0.00000 0.00017 0.00000

RIER --- 0141 2* -----------.---------.----------I------------------ 792 395 42 2 0.03378 0.03339 0.00711 0.00011

This (N/K) is the most frequent escape form at d20, and is also the B consensus form, confers escape from polyclonal responses [1,3]*.* Conventional sequencing from subsequent time points indicate that it is common only transiently, and was rare by day 72:

WEAU d20 0050 3 -----------.--K------.----------I------------------ 0 3 0 0 0.00000 0.00025 0.00000 0.00000

WEAU d10 0015 11 -----------.--K------.----------------------------- 11 425 1356 62 0.00047 0.03593 0.22944 0.00328

WEAU d20 0002 425 -----------.--K------.----------------------------- 11 425 1356 62 0.00047 0.03593 0.22944 0.00328

WEAU d20 0039 4 ----------V.--K------.----------------------------- 0 4 0 0 0.00000 0.00034 0.00000 0.00000

WEAU d20 0115 1 -----------.--K------.---------N------------------- 0 1 0 0 0.00000 0.00008 0.00000 0.00000

WEAU d20 0121 1 -----------.--K------.------------------------D---- 0 1 0 0 0.00000 0.00008 0.00000 0.00000

WEAU d20 0139 1 --P--------.--K------.----------------------------- 0 1 0 0 0.00000 0.00008 0.00000 0.00000

WEAU d20 0161 1 -----------.--K------.--------------Y-------------- 0 1 0 0 0.00000 0.00008 0.00000 0.00000

WEAU d20 0183 1 -----------.--K------.--------T-------------------- 0 1 0 0 0.00000 0.00008 0.00000 0.00000

WEAU d20 0197 1 -----------.--K------.----------------------G------ 0 1 0 0 0.00000 0.00008 0.00000 0.00000

WEAU d20 0205 1 ------T----.--K------.----------------------------- 0 1 0 0 0.00000 0.00008 0.00000 0.00000

WEAU d20 0208 1 -----------.--K------.--------------F-------------- 0 1 0 0 0.00000 0.00008 0.00000 0.00000

WEAU d20 0211 1 -----------.--K------.----G------------------------ 0 1 0 0 0.00000 0.00008 0.00000 0.00000

WEAU d30 0002 1356 -----------.--K------.----------------------------- 11 425 1356 62 0.00047 0.03593 0.22944 0.00328

WEAU d30 0023 3 -----------.--K--I---.----------------------------- 0 0 3 0 0.00000 0.00000 0.00051 0.00000

WEAU d30 0028 3 -----------.--K------.-------------L--------------- 0 0 3 0 0.00000 0.00000 0.00051 0.00000

WEAU d30 0034 2 -----------.--K------.--------------------T-------- 0 0 2 0 0.00000 0.00000 0.00034 0.00000

WEAU d30 0037 2 -F---------.--K------.----------------------------- 0 0 2 0 0.00000 0.00000 0.00034 0.00000

WEAU d30 0038 2 -----------.--K------.------E-------------T-------- 0 0 2 0 0.00000 0.00000 0.00034 0.00000

WEAU d30 0039 2 -----------.--K------.------N---------------------- 0 0 2 0 0.00000 0.00000 0.00034 0.00000

WEAU d30 0045 2 ---------N-.--K------.----------------------------- 0 0 2 0 0.00000 0.00000 0.00034 0.00000

WEAU d30 0047 2 -----------.--K------.----G-E---------------------- 0 0 2 0 0.00000 0.00000 0.00034 0.00000

WEAU d30 0052 1 -----------.--K------.----------------------N------ 0 0 1 0 0.00000 0.00000 0.00017 0.00000

WEAU d30 0058 1 -----------.--K------.-----------I----------------- 0 0 1 0 0.00000 0.00000 0.00017 0.00000

WEAU d30 0060 1 -----------.--K------.-----------------------A----- 0 0 1 1 0.00000 0.00000 0.00017 0.00005

WEAU d30 0064 1 -----------.--K------.-------------------R--------- 0 0 1 0 0.00000 0.00000 0.00017 0.00000

WEAU d30 0065 1 -----S-----.--K------.----------------------------- 0 0 1 0 0.00000 0.00000 0.00017 0.00000

WEAU d30 0077 1 -----------.--K------.---------A------------------- 0 0 1 0 0.00000 0.00000 0.00017 0.00000

WEAU d30 0080 1 ----T------.--K------.----------------------------- 0 0 1 1 0.00000 0.00000 0.00017 0.00005

WEAU d30 0081 1 -----------.--K----G-.----------------------------- 0 0 1 0 0.00000 0.00000 0.00017 0.00000

WEAU d30 0085 1 I----------.--K------.----------------------------- 0 0 1 0 0.00000 0.00000 0.00017 0.00000

RIER --- 0012 62 -----------.--K------.----------------------------- 11 425 1356 62 0.00047 0.03593 0.22944 0.00328

Second most common form (A/T), but not "escape" from polyclonal or clone 5-1 T cell responses [1,3]; it might be a processing escape:

WEAU d20 0119 1 -----------.T-K------.----------------------------- 0 1 3 0 0.00000 0.00008 0.00051 0.00000

WEAU d20 0018 9 -----------.T--------.----------I------------------ 0 9 0 0 0.00000 0.00076 0.00000 0.00000

WEAU d10 0003 35 -----------.T--------.----------------------------- 35 335 630 13 0.00149 0.02832 0.10660 0.00069

WEAU d20 0004 335 -----------.T--------.----------------------------- 35 335 630 13 0.00149 0.02832 0.10660 0.00069

WEAU d20 0083 2 -----------.T--------.------E---------------------- 0 2 0 0 0.00000 0.00017 0.00000 0.00000

WEAU d20 0097 1 -----------.T------D-.----------------------------- 0 1 0 0 0.00000 0.00008 0.00000 0.00000

WEAU d20 0138 1 -----------.T--------.-------D--------------------- 0 1 0 9 0.00000 0.00008 0.00000 0.00048

WEAU d20 0142 1 -----------.T--------.H---------------------------- 0 1 0 0 0.00000 0.00008 0.00000 0.00000

WEAU d20 0164 1 --------G--.T--------.----------------------------- 0 1 0 0 0.00000 0.00008 0.00000 0.00000

WEAU d20 0182 1 ------I----.T--------.----------------------------- 0 1 1 0 0.00000 0.00008 0.00017 0.00000

WEAU d20 0185 1 -----------.T--------.-----------------------A----- 0 1 8 0 0.00000 0.00008 0.00135 0.00000

WEAU d20 0200 1 -P---------.T--------.----------------------------- 0 1 0 0 0.00000 0.00008 0.00000 0.00000

WEAU d20 0201 1 -----------.T--------.--------------------T-------- 0 1 0 0 0.00000 0.00008 0.00000 0.00000

WEAU d30 0003 630 -----------.T--------.----------------------------- 35 335 630 13 0.00149 0.02832 0.10660 0.00069

WEAU d30 0016 8 -----------.T--------.-----------------------A----- 0 1 8 0 0.00000 0.00008 0.00135 0.00000

WEAU d30 0035 2 -----------.T--------.---------I------------------- 0 0 2 0 0.00000 0.00000 0.00034 0.00000

WEAU d30 0049 1 -----------.T--F-----.----------------------------- 0 0 1 0 0.00000 0.00000 0.00017 0.00000

WEAU d30 0050 1 -----------.T--------.----A------------------------ 0 0 1 0 0.00000 0.00000 0.00017 0.00000

WEAU d30 0066 1 ------I----.T--------.----------------------------- 0 1 1 0 0.00000 0.00008 0.00017 0.00000

WEAU d30 0068 1 -----------.T-----I--.----------------------------- 0 0 1 0 0.00000 0.00000 0.00017 0.00000

WEAU d30 0074 1 ---------R-.T--------.----------------------------- 0 0 1 0 0.00000 0.00000 0.00017 0.00000

WEAU d30 0084 1 -----------.T--------.--------------G-------------- 0 0 1 0 0.00000 0.00000 0.00017 0.00000

RIER --- 0024 13 -----------.T--------.----------------------------- 35 335 630 13 0.00149 0.02832 0.10660 0.00069

3rd most common form (A/V), the double mutant V--S----- and it confers escape, but the single mutant was not tested: V-------- [1,3]*:*

WEAU d20 0021 7 -----------.V--------.----------I------------------ 0 7 3 0 0.00000 0.00059 0.00051 0.00000

WEAU d30 0030 3 -----------.V--------.----------I------------------ 0 7 3 0 0.00000 0.00059 0.00051 0.00000

WEAU d10 0016 11 -----------.V--------.----------------------------- 11 232 616 4 0.00047 0.01961 0.10423 0.00021

WEAU d20 0006 232 -----------.V--------.----------------------------- 11 232 616 4 0.00047 0.01961 0.10423 0.00021

WEAU d20 0068 2 -----------.V--------.-----------------------A----- 0 2 0 0 0.00000 0.00017 0.00000 0.00000

WEAU d20 0076 2 ----------V.V--------.----------------------------- 0 2 0 0 0.00000 0.00017 0.00000 0.00000

WEAU d20 0101 1 -----------.V--------.--------------------------Y-- 0 1 0 0 0.00000 0.00008 0.00000 0.00000

WEAU d20 0103 1 -----------.V--------.----------------------N------ 0 1 0 0 0.00000 0.00008 0.00000 0.00000

WEAU d20 0112 1 -----------.V--------.------R---------------------- 0 1 2 0 0.00000 0.00008 0.00034 0.00000

WEAU d20 0116 1 -----------.V--------.--------------F-------------- 0 1 0 0 0.00000 0.00008 0.00000 0.00000

WEAU d20 0166 1 ---V-------.V--------.----------------------------- 0 1 0 0 0.00000 0.00008 0.00000 0.00000

WEAU d20 0195 1 ---E-------.V--------.----------------------------- 0 1 0 0 0.00000 0.00008 0.00000 0.00000

WEAU d20 0198 1 ---E-------.V--------.------------------T---------- 0 1 0 0 0.00000 0.00008 0.00000 0.00000

WEAU d30 0004 616 -----------.V--------.----------------------------- 11 232 616 4 0.00047 0.01961 0.10423 0.00021

WEAU d30 0021 4 -----------.V-K------.----------------------------- 0 0 4 0 0.00000 0.00000 0.00068 0.00000

WEAU d30 0027 3 T----------.V--------.----------------------------- 0 0 3 0 0.00000 0.00000 0.00051 0.00000

WEAU d30 0036 2 -----------.V--------.------R---------------------- 0 1 2 0 0.00000 0.00008 0.00034 0.00000

WEAU d30 0046 2 -----------.V--------.---L------------------------- 0 0 2 0 0.00000 0.00000 0.00034 0.00000

WEAU d30 0075 1 -----------.V--------.------E---------------------- 0 0 1 0 0.00000 0.00000 0.00017 0.00000

WEAU d30 0076 1 -----------.V---R----.----------------------------- 0 0 1 0 0.00000 0.00000 0.00017 0.00000

RIER --- 0087 4 -----------.V--------.----------------------------- 11 232 616 4 0.00047 0.01961 0.10423 0.00021

4th most common form (E/K), tested and this mutation confers escape [1,3]*.*; the substitution is in a B*44 anchor residue:

WEAU d20 0060 2 -----------.-K-------.----------I------------------ 0 2 2 0 0.00000 0.00017 0.00034 0.00000

WEAU d30 0040 2 -----------.-K-------.----------I------------------ 0 2 2 0 0.00000 0.00017 0.00034 0.00000

WEAU d20 0124 1 -----------.-KK------.----------------------------- 0 1 0 0 0.00000 0.00008 0.00000 0.00000

WEAU d10 0008 14 -----------.-K-------.----------------------------- 14 235 438 2 0.00060 0.01986 0.07411 0.00011

WEAU d20 0005 235 -----------.-K-------.----------------------------- 14 235 438 2 0.00060 0.01986 0.07411 0.00011

WEAU d20 0079 2 -----------.-K-------.--I-------------------------- 0 2 0 0 0.00000 0.00017 0.00000 0.00000

WEAU d20 0107 1 -----------.-K-------.---S------------------------- 0 1 0 0 0.00000 0.00008 0.00000 0.00000

WEAU d20 0127 1 -----------.-K-------.----------------------------I 0 1 0 0 0.00000 0.00008 0.00000 0.00000

WEAU d20 0143 1 ----------S.-K-------.----------------------------- 0 1 0 0 0.00000 0.00008 0.00000 0.00000

WEAU d20 0148 1 -----------.-K-------.-------------------------A--- 0 1 0 0 0.00000 0.00008 0.00000 0.00000

WEAU d20 0158 1 -----------.-K-------.-----------------------A----- 0 1 0 0 0.00000 0.00008 0.00000 0.00000

WEAU d20 0172 1 -----------.-K-------.-------------------------I--- 0 1 1 0 0.00000 0.00008 0.00017 0.00000

WEAU d20 0189 1 -----------.-K---I---.----------------------------- 0 1 0 0 0.00000 0.00008 0.00000 0.00000

WEAU d20 0209 1 -----------.-K-------.---------------------H------- 0 1 0 0 0.00000 0.00008 0.00000 0.00000

WEAU d30 0005 438 -----------.-K-------.----------------------------- 14 235 438 2 0.00060 0.01986 0.07411 0.00011

WEAU d30 0026 3 -------S---.-K-------.----------------------------- 0 0 3 0 0.00000 0.00000 0.00051 0.00000

WEAU d30 0051 1 -----------.-K-------.-----G----------------------- 0 0 1 0 0.00000 0.00000 0.00017 0.00000

WEAU d30 0056 1 -----------.-K-------.-------------------------I--- 0 1 1 0 0.00000 0.00008 0.00017 0.00000

WEAU d30 0079 1 -----------.-K-------.-----------------E----------- 0 0 1 0 0.00000 0.00000 0.00017 0.00000

RIER --- 0147 2 -----------.-K-------.----------------------------- 14 235 438 2 0.00060 0.01986 0.07411 0.00011

5th most common form (E/G), tested and this mutation confers escape [1,3]; substitution is in a B*44 anchor residue:

WEAU d20 0070 2 -----------.-G-------.----------I------------------ 0 2 0 0 0.00000 0.00017 0.00000 0.00000

WEAU d20 0207 1 -----------.TG-------.----------------------------- 0 1 0 0 0.00000 0.00008 0.00000 0.00000

WEAU d20 0007 108 -----------.-G-------.----------------------------- 0 108 276 381 0.00000 0.00913 0.04670 0.02016

WEAU d20 0150 1 -----------.-G-------.--A-------------------------- 0 1 0 0 0.00000 0.00008 0.00000 0.00000

WEAU d30 0006 276 -----------.-G-------.----------------------------- 0 108 276 381 0.00000 0.00913 0.04670 0.02016

WEAU d30 0015 10 -----------.-GK------.----------------------------- 0 0 10 0 0.00000 0.00000 0.00169 0.00000

WEAU d30 0055 1 -----------.-G-------.-----------A----------------- 0 0 1 0 0.00000 0.00000 0.00017 0.00000

WEAU d30 0057 1 -----------.-G-------.-----------------------A----- 0 0 1 7 0.00000 0.00000 0.00017 0.00037

WEAU d30 0070 1 -----------.-G-------.----------P------------------ 0 0 1 0 0.00000 0.00000 0.00017 0.00000

WEAU d30 0078 1 -----------.-G-------.---L------------------------- 0 0 1 0 0.00000 0.00000 0.00017 0.00000

RIER --- 0006 381 -----------.-G-------.----------------------------- 0 108 276 381 0.00000 0.00913 0.04670 0.02016

Enriched form (N/D), phenotype unknown:

WEAU d20 0203 1 -----------.--D------.----------I------------------ 0 1 0 0 0.00000 0.00008 0.00000 0.00000

WEAU d30 0029 3 -----------.T-D------.----------------------------- 0 0 3 0 0.00000 0.00000 0.00051 0.00000

WEAU d20 0186 1 -----------.-GD------.----------------------------- 0 1 0 0 0.00000 0.00008 0.00000 0.00000

WEAU d20 0011 22 -----------.--D------.----------------------------- 0 22 65 1 0.00000 0.00186 0.01100 0.00005

WEAU d30 0007 65 -----------.--D------.----------------------------- 0 22 65 1 0.00000 0.00186 0.01100 0.00005

RIER --- 10377 1 -----------.--D------.----------------------------- 0 22 65 1 0.00000 0.00186 0.01100 0.00005

Enriched form (V/I): -------I- phenotype unknown, T------I- double mutation does not confer escape [1,3]:

WEAU d30 0022 3 -----------.T------I-.----------------------------- 0 0 3 0 0.00000 0.00000 0.00051 0.00000

WEAU d10 0024 8 -----------.-------I-.----------------------------- 8 48 65 3 0.00034 0.00406 0.01100 0.00016

WEAU d20 0008 48 -----------.-------I-.----------------------------- 8 48 65 3 0.00034 0.00406 0.01100 0.00016

WEAU d20 0167 1 -----------.-------I-.----------A------------------ 0 1 0 0 0.00000 0.00008 0.00000 0.00000

WEAU d30 0008 65 -----------.-------I-.----------------------------- 8 48 65 3 0.00034 0.00406 0.01100 0.00016

RIER --- 0105 3 -----------.-------I-.----------------------------- 8 48 65 3 0.00034 0.00406 0.01100 0.00016

Enriched form (E/A) was tested and confers escape [1,3];; substitution is in a B*44 anchor residue:

WEAU d20 0009 23 -----------.-A-------.----------------------------- 0 23 46 0 0.00000 0.00194 0.00778 0.00000

WEAU d30 0009 46 -----------.-A-------.----------------------------- 0 23 46 0 0.00000 0.00194 0.00778 0.00000

RIER --- 0049 7 -----------.-A-------.-------D--------------------- 0 0 0 7 0.00000 0.00000 0.00000 0.00037

Enriched form (N/S), escape phenotype unknown:

WEAU d30 0019 4 -----------.V-S------.----------------------------- 0 0 4 0 0.00000 0.00000 0.00068 0.00000

WEAU d10 0033 6 -----------.--S------.----------------------------- 6 22 36 0 0.00026 0.00186 0.00609 0.00000

WEAU d20 0010 22 -----------.--S------.----------------------------- 6 22 36 0 0.00026 0.00186 0.00609 0.00000

WEAU d20 0104 1 -----------.--S------.----------S------------------ 0 1 1 0 0.00000 0.00008 0.00017 0.00000

WEAU d20 0125 1 -----------.--S------.----------A------------------ 0 1 0 0 0.00000 0.00008 0.00000 0.00000

WEAU d30 0011 36 -----------.--S------.----------------------------- 6 22 36 0 0.00026 0.00186 0.00609 0.00000

WEAU d30 0053 1 -----------.--S------.----------S------------------ 0 1 1 0 0.00000 0.00008 0.00017 0.00000

RIER --- 0172 2 -----------.-GS------.----------------------------- 0 0 0 2 0.00000 0.00000 0.00000 0.00011

Enriched form (E/D), escape phenotype unknown:

WEAU d20 0016 10 -----------.-D-------.----------------------------- 0 10 26 3700 0.00000 0.00085 0.00440 0.19573

WEAU d30 0012 26 -----------.-D-------.----------------------------- 0 10 26 3700 0.00000 0.00085 0.00440 0.19573

RIER --- 0002 3703 -----------.-D-------.----------------------------- 0 10 26 3700 0.00000 0.00085 0.00440 0.19573

Enriched form (L/S), escape phenotype unknown:

WEAU d20 0012 18 -----------.---S-----.----------------------------- 0 18 12 1 0.00000 0.00152 0.00203 0.00005

WEAU d30 0013 12 -----------.---S-----.----------------------------- 0 18 12 1 0.00000 0.00152 0.00203 0.00005

RIER --- 0230 1 -----------.---S-----.----------------------------- 0 18 12 1 0.00000 0.00152 0.00203 0.00005

Enriched form (V/A), escape phenotype unknown:

WEAU d10 0031 7 -----------.-----A---.----------------------------- 7 11 10 0 0.00030 0.00093 0.00169 0.00000

WEAU d20 0014 11 -----------.-----A---.----------------------------- 7 11 10 0 0.00030 0.00093 0.00169 0.00000

WEAU d20 0120 1 -----------.-----A---.------------------------K---- 0 1 0 0 0.00000 0.00008 0.00000 0.00000

WEAU d30 0014 10 -----------.-----A---.----------------------------- 7 11 10 0 0.00030 0.00093 0.00169 0.00000

RIER --- 0261 1 -----------.-----A---.-------D--------------------- 0 0 0 1 0.00000 0.00000 0.00000 0.00005

Other day 10 sequences:

WEAU d10 0005 20 -----------.---------.----------------L------------ 20 3 0 7 0.00085 0.00025 0.00000 0.00037

WEAU d10 0006 15 -----------.---------.--------------Y-------------- 15 3 0 2 0.00064 0.00025 0.00000 0.00011

WEAU d10 0007 14 -----------.---------.-E--------------------------- 14 8 0 1 0.00060 0.00068 0.00000 0.00005

WEAU d10 0010 13 -----------.---------.-R--------------------------- 13 2 1 5 0.00055 0.00017 0.00017 0.00026

WEAU d10 0011 12 -----------.---------.-----------I----------------- 12 3 1 7 0.00051 0.00025 0.00017 0.00037

WEAU d10 0012 11 ------I----.---------.----------------------------- 11 6 0 0 0.00047 0.00051 0.00000 0.00000

WEAU d10 0013 11 ---E-------.---------.----------------------------- 11 3 0 0 0.00047 0.00025 0.00000 0.00000

WEAU d10 0014 11 -----------.---------.---------------------H------- 11 7 5 3 0.00047 0.00059 0.00085 0.00016

WEAU d10 0017 10 -----------.---------.---------I------------------- 10 5 2 11 0.00043 0.00042 0.00034 0.00058

WEAU d10 0018 10 -----------.---------.-----------------------A----- 10 3 1 53 0.00043 0.00025 0.00017 0.00280

WEAU d10 0020 9 -----------.---------.---------------V------------- 9 6 0 2 0.00038 0.00051 0.00000 0.00011

WEAU d10 0021 9 --------Y--.---------.----------------------------- 9 2 2 1 0.00038 0.00017 0.00034 0.00005

WEAU d10 0022 9 -----------.-------G-.----------------------------- 9 9 0 0 0.00038 0.00076 0.00000 0.00000

WEAU d10 0023 8 -----------.---------.--I-------------------------- 8 4 0 2 0.00034 0.00034 0.00000 0.00011

WEAU d10 0026 8 V----------.---------.----------------------------- 8 0 0 0 0.00034 0.00000 0.00000 0.00000

WEAU d10 0027 7 -----------.---------.---S------------------------- 7 6 0 2 0.00030 0.00051 0.00000 0.00011

WEAU d10 0028 7 --F--------.---------.----------------------------- 7 3 2 0 0.00030 0.00025 0.00034 0.00000

WEAU d10 0029 7 -----------.---------.------E---------------------- 7 5 0 3 0.00030 0.00042 0.00000 0.00016

WEAU d10 0030 7 -----------.----R----.----------------------------- 7 2 0 1 0.00030 0.00017 0.00000 0.00005

WEAU d10 0032 7 ----------V.---------.----------------------------- 7 3 0 3 0.00030 0.00025 0.00000 0.00016

WEAU d10 0034 6 -----------.------I--.----------------------------- 6 1 0 2 0.00026 0.00008 0.00000 0.00011

WEAU d10 0036 6 -----------.---------.--------------------S-------- 6 0 0 1 0.00026 0.00000 0.00000 0.00005

WEAU d10 0037 6 -----------.---------.------------------T---------- 6 3 0 0 0.00026 0.00025 0.00000 0.00000

WEAU d10 0038 6 ----------T.---------.----------------------------- 6 3 0 11 0.00026 0.00025 0.00000 0.00058

WEAU d10 0039 6 -----------.--Y------.----------------------------- 6 0 0 0 0.00026 0.00000 0.00000 0.00000

WEAU d10 0040 6 ----L------.---------.----------------------------- 6 0 0 0 0.00026 0.00000 0.00000 0.00000

WEAU d10 0041 6 -----------.-----I---.----------------------------- 6 1 0 2 0.00026 0.00008 0.00000 0.00011

WEAU d10 0042 6 -----------.--------C.----------------------------- 6 5 0 0 0.00026 0.00042 0.00000 0.00000

WEAU d10 0043 5 -----------.--------H.----------------------------- 5 8 0 0 0.00021 0.00068 0.00000 0.00000

WEAU d10 0045 5 -----------.---------.--------V-------------------- 5 4 0 2 0.00021 0.00034 0.00000 0.00011

WEAU d10 0046 5 -----------.---------.----G------------------------ 5 2 0 0 0.00021 0.00017 0.00000 0.00000

WEAU d10 0047 5 -----------.---------.--E-------------------------- 5 0 0 0 0.00021 0.00000 0.00000 0.00000

WEAU d10 0048 5 -----------.---------.--------------------V-------- 5 3 0 3 0.00021 0.00025 0.00000 0.00016

WEAU d10 0050 5 -----------.-------A-.----------------------------- 5 4 0 1 0.00021 0.00034 0.00000 0.00005

WEAU d10 0051 5 -P---------.---------.----------------------------- 5 1 0 0 0.00021 0.00008 0.00000 0.00000

WEAU d10 0052 5 -----------.---------.--------T-------------------- 5 2 0 135 0.00021 0.00017 0.00000 0.00714

WEAU d10 0053 4 -----------.---------.-------------------------A--- 4 0 0 19 0.00017 0.00000 0.00000 0.00101

WEAU d10 0054 4 -----------.---------.-----------------------I----- 4 1 0 1 0.00017 0.00008 0.00000 0.00005

WEAU d10 0057 4 -----------.---------.------------------V---------- 4 2 0 0 0.00017 0.00017 0.00000 0.00000

WEAU d10 0058 4 -----------.-----G---.----------------------------- 4 3 0 0 0.00017 0.00025 0.00000 0.00000

WEAU d10 0059 4 --------F--.---------.----------------------------- 4 0 0 0 0.00017 0.00000 0.00000 0.00000

WEAU d10 0060 4 -----------.---------.---------------------------K- 4 0 0 0 0.00017 0.00000 0.00000 0.00000

WEAU d10 0061 4 -----------.---------.--A-------------------------- 4 2 0 0 0.00017 0.00017 0.00000 0.00000

WEAU d10 0062 4 -----------.---------.-------K--------------------- 4 5 0 3 0.00017 0.00042 0.00000 0.00016

WEAU d10 0063 4 I----------.---------.----------------------------- 4 3 0 1 0.00017 0.00025 0.00000 0.00005

WEAU d10 0056 3 -----------.---------.---T------------------------- 3 0 0 0 0.00013 0.00000 0.00000 0.00000

WEAU d10 0064 3 -----------.---------.-----C----------------------- 3 1 0 1 0.00013 0.00008 0.00000 0.00005

WEAU d10 0065 3 -----------.---------.-------G--------------------- 3 1 0 3 0.00013 0.00008 0.00000 0.00016

WEAU d10 0066 3 -----------.---------.--------------F-------------- 3 0 0 1 0.00013 0.00000 0.00000 0.00005

WEAU d10 0067 3 -----------.---------.----------------A------------ 3 1 0 0 0.00013 0.00008 0.00000 0.00000

WEAU d10 0068 3 ---R-------.---------.----------------------------- 3 0 0 0 0.00013 0.00000 0.00000 0.00000

WEAU d10 0069 3 ----------D.---------.----------------------------- 3 1 0 6 0.00013 0.00008 0.00000 0.00032

WEAU d10 0070 3 -----------.---------.S---------------------------- 3 1 0 0 0.00013 0.00008 0.00000 0.00000

WEAU d10 0072 3 -----------.---------.-----------A----------------- 3 0 0 0 0.00013 0.00000 0.00000 0.00000

WEAU d10 0073 3 -----------.---------.----------S------------------ 3 1 0 1 0.00013 0.00008 0.00000 0.00005

WEAU d10 0074 3 -----------.---V-----.----------------------------- 3 3 0 0 0.00013 0.00025 0.00000 0.00000

WEAU d10 0075 3 ---W-------.---------.----------------------------- 3 0 0 1 0.00013 0.00000 0.00000 0.00005

WEAU d10 0076 3 -----------.---------.---L------------------------- 3 2 0 4 0.00013 0.00017 0.00000 0.00021

WEAU d10 0077 3 -----------.---------.------------Q---------------- 3 0 0 0 0.00013 0.00000 0.00000 0.00000

WEAU d10 0078 3 -----------.---------.-------------S--------------- 3 2 0 0 0.00013 0.00017 0.00000 0.00000

WEAU d10 0079 3 --------R--.---------.----------------------------- 3 1 0 2 0.00013 0.00008 0.00000 0.00011

WEAU d10 0080 3 -----------.---------.---H------------------------- 3 0 0 0 0.00013 0.00000 0.00000 0.00000

WEAU d10 0081 2 -----------.-----D---.----------------------------- 2 0 0 0 0.00009 0.00000 0.00000 0.00000

WEAU d10 0082 2 -----------.---------.--------------------------Y-- 2 0 1 2 0.00009 0.00000 0.00017 0.00011

WEAU d10 0083 2 -----------.---------.----------------------Y------ 2 0 0 3 0.00009 0.00000 0.00000 0.00016

WEAU d10 0084 2 -----------.---------.------------------------K---- 2 0 0 1 0.00009 0.00000 0.00000 0.00005

WEAU d10 0085 2 -----------.---------.------T---------------------- 2 0 0 1 0.00009 0.00000 0.00000 0.00005

WEAU d10 0086 2 -----------.---------.---------A------------------- 2 0 0 1 0.00009 0.00000 0.00000 0.00005

WEAU d10 0088 2 -----------.---------.--------------------T-------- 2 2 2 0 0.00009 0.00017 0.00034 0.00000

WEAU d10 0090 2 ---------I-.---------.----------------------------- 2 0 0 1 0.00009 0.00000 0.00000 0.00005

WEAU d10 0091 2 -F---------.---------.----------------------------- 2 1 0 4 0.00009 0.00008 0.00000 0.00021

WEAU d10 0092 2 -----------.---------.-----G----------------------- 2 2 0 0 0.00009 0.00017 0.00000 0.00000

WEAU d10 0093 2 ---------N-.---------.----------------------------- 2 1 0 13 0.00009 0.00008 0.00000 0.00069

WEAU d10 0094 2 -----------.---------.--------------R-------------- 2 1 0 0 0.00009 0.00008 0.00000 0.00000

WEAU d10 0095 2 -----------.---------.---------------E------------- 2 0 0 0 0.00009 0.00000 0.00000 0.00000

WEAU d10 0096 2 -----------.------A--.----------------------------- 2 7 1 1 0.00009 0.00059 0.00017 0.00005

WEAU d10 0100 1 ------V----.---------.----------------------------- 1 1 1 0 0.00004 0.00008 0.00017 0.00000

WEAU d10 0101 1 -----------.---------.--------------------E-------- 1 0 0 0 0.00004 0.00000 0.00000 0.00000

WEAU d10 0102 1 ---------G-.---------.----------------------------- 1 0 0 0 0.00004 0.00000 0.00000 0.00000

WEAU d10 0105 1 -----------.-------D-.----------------------------- 1 1 0 0 0.00004 0.00008 0.00000 0.00000

WEAU d10 0106 1 --------G--.---------.----------------------------- 1 0 0 4 0.00004 0.00000 0.00000 0.00021

WEAU d10 0107 1 ---------T-.---------.----------------------------- 1 0 0 0 0.00004 0.00000 0.00000 0.00000

WEAU d10 0108 1 -----------.---------.-----------------Y----------- 1 0 0 0 0.00004 0.00000 0.00000 0.00000

WEAU d10 0109 1 -----------.---------.----A------------------------ 1 0 0 1 0.00004 0.00000 0.00000 0.00005

WEAU d10 0110 1 I--A-------.---------.----------------------------- 1 0 0 0 0.00004 0.00000 0.00000 0.00000

WEAU d10 0112 1 -----------.---------.----------------------------A 1 0 0 1 0.00004 0.00000 0.00000 0.00005

WEAU d10 0114 1 -----------.----G----.----------------L------------ 1 0 0 0 0.00004 0.00000 0.00000 0.00000

WEAU d10 0115 1 -----------.---------.------------------G---------- 1 1 0 0 0.00004 0.00008 0.00000 0.00000

WEAU d10 0117 1 -----------.---------.--L-------------------------- 1 1 0 1 0.00004 0.00008 0.00000 0.00005

WEAU d10 0118 1 -----------.---------.-------------------------L--- 1 1 0 0 0.00004 0.00008 0.00000 0.00000

WEAU d10 0119 1 -----------.---------.------------------S---------- 1 0 0 2 0.00004 0.00000 0.00000 0.00011

WEAU d10 0120 1 -----------.---------.-----------S----------------- 1 0 0 0 0.00004 0.00000 0.00000 0.00000

WEAU d10 0121 1 -----------.---------.--------------W-------------- 1 0 0 0 0.00004 0.00000 0.00000 0.00000

WEAU d10 0122 1 -----------.---------.-------------------------I--- 1 0 0 13 0.00004 0.00000 0.00000 0.00069

WEAU d10 0124 1 -----------.------S--.----------------------------- 1 1 0 0 0.00004 0.00008 0.00000 0.00000

WEAU d10 0126 1 -----------.---------.-----R----------------------- 1 2 0 1 0.00004 0.00017 0.00000 0.00005

WEAU d10 0127 1 -----------.---------.-----------------------S----- 1 0 0 0 0.00004 0.00000 0.00000 0.00000

WEAU d10 0128 1 --P--------.---------.----------------------------- 1 1 2 0 0.00004 0.00008 0.00034 0.00000

WEAU d10 0129 1 -----------.---------.-------------L--------------- 1 1 0 0 0.00004 0.00008 0.00000 0.00000

WEAU d10 0130 1 ---V-------.---------.----------------------------- 1 1 0 1 0.00004 0.00008 0.00000 0.00005

WEAU d10 0132 1 -----------.---------.----M------------------------ 1 2 0 4 0.00004 0.00017 0.00000 0.00021

WEAU d10 0133 1 -----------.---------.-------------I--------------- 1 0 0 0 0.00004 0.00000 0.00000 0.00000

WEAU d10 0134 1 -----------.---------.C---------------------------- 1 0 0 0 0.00004 0.00000 0.00000 0.00000

WEAU d10 0135 1 -----------.---------.---------------G------------- 1 0 0 1 0.00004 0.00000 0.00000 0.00005

WEAU d10 0136 1 -----------.---------.-----------N----------------- 1 0 0 0 0.00004 0.00000 0.00000 0.00000

WEAU d10 0137 1 -----------.---------.----------A------------------ 1 3 0 1 0.00004 0.00025 0.00000 0.00005

WEAU d10 0138 1 -R---------.---------.----------------------------- 1 0 0 0 0.00004 0.00000 0.00000 0.00000

WEAU d10 0139 1 -----------.---------.----------------------N------ 1 1 0 0 0.00004 0.00008 0.00000 0.00000

WEAU d10 0140 1 --R--------.---------.----------------------------- 1 1 0 0 0.00004 0.00008 0.00000 0.00000

WEAU d10 0141 1 -----------.---F-----.----------------------------- 1 1 0 0 0.00004 0.00008 0.00000 0.00000

WEAU d10 0142 1 -----------.---------.-----------------N----------- 1 5 0 10 0.00004 0.00042 0.00000 0.00053

WEAU d10 0143 1 -----------.---------.-W--------------------------- 1 0 0 0 0.00004 0.00000 0.00000 0.00000

WEAU d10 0144 1 ------K----.---------.----------------------------- 1 0 0 0 0.00004 0.00000 0.00000 0.00000

WEAU d10 0145 1 -----------.---------.-----L----------------------- 1 0 0 0 0.00004 0.00000 0.00000 0.00000

WEAU d10 0146 1 -----------.---------.-------------------N--------- 1 0 0 0 0.00004 0.00000 0.00000 0.00000

WEAU d10 0147 1 -----------.---------.--------------G-------------- 1 0 0 0 0.00004 0.00000 0.00000 0.00000

WEAU d10 0148 1 -----------.---------.-------------------R--------- 1 1 0 0 0.00004 0.00008 0.00000 0.00000

WEAU d10 0149 1 -----------.---------.---------------------------D- 1 1 0 0 0.00004 0.00008 0.00000 0.00000

WEAU d10 0150 1 --F--------.---------.--------------F-------------- 1 0 0 0 0.00004 0.00000 0.00000 0.00000

WEAU d10 0151 1 -----------.S--------.----------------------------- 1 0 0 0 0.00004 0.00000 0.00000 0.00000

WEAU d10 0152 1 -----------.---------.------------------------D---- 1 1 0 0 0.00004 0.00008 0.00000 0.00000

WEAU d10 0155 1 -----------.---------.-------------Y--------------- 1 0 0 0 0.00004 0.00000 0.00000 0.00000

WEAU d10 0156 1 -----------.---------.----L------------------------ 1 1 0 1 0.00004 0.00008 0.00000 0.00005

Other d20 sequences:

WEAU d20 0015 10 -----------.---------.---------------T------------- 0 10 0 0 0.00000 0.00085 0.00000 0.00000

WEAU d20 0017 9 -----------.-------G-.----------------------------- 9 9 0 0 0.00038 0.00076 0.00000 0.00000

WEAU d20 0019 8 -----------.--------H.----------------------------- 5 8 0 0 0.00021 0.00068 0.00000 0.00000

WEAU d20 0020 8 -----------.---------.-E--------------------------- 14 8 0 1 0.00060 0.00068 0.00000 0.00005

WEAU d20 0022 7 -----------.---------.---------------------H------- 11 7 5 3 0.00047 0.00059 0.00085 0.00016

WEAU d20 0023 7 -----------.------A--.----------------------------- 2 7 1 1 0.00009 0.00059 0.00017 0.00005

WEAU d20 0024 6 ------I----.---------.----------------------------- 11 6 0 0 0.00047 0.00051 0.00000 0.00000

WEAU d20 0025 6 -----------.---------.---S------------------------- 7 6 0 2 0.00030 0.00051 0.00000 0.00011

WEAU d20 0026 6 -----------.---------.---------------V------------- 9 6 0 2 0.00038 0.00051 0.00000 0.00011

WEAU d20 0028 5 -----------.---------.---------I------------------- 10 5 2 11 0.00043 0.00042 0.00034 0.00058

WEAU d20 0029 5 -----------.---------.------E---------------------- 7 5 0 3 0.00030 0.00042 0.00000 0.00016

WEAU d20 0031 5 -----------.---------.-----------------N----------- 1 5 0 10 0.00004 0.00042 0.00000 0.00053

WEAU d20 0032 5 -----------.---------.-------K--------------------- 4 5 0 3 0.00017 0.00042 0.00000 0.00016

WEAU d20 0033 5 -----------.--------C.----------------------------- 6 5 0 0 0.00026 0.00042 0.00000 0.00000

WEAU d20 0034 4 -----------.---------.--I-------------------------- 8 4 0 2 0.00034 0.00034 0.00000 0.00011

WEAU d20 0035 4 -------L---.---------.----------------------------- 0 4 0 0 0.00000 0.00034 0.00000 0.00000

WEAU d20 0036 4 -----------.---------.--------V-------------------- 5 4 0 2 0.00021 0.00034 0.00000 0.00011

WEAU d20 0037 4 -----------.-------A-.----------------------------- 5 4 0 1 0.00021 0.00034 0.00000 0.00005

WEAU d20 0038 4 -----------.--T------.----------------------------- 0 4 3 0 0.00000 0.00034 0.00051 0.00000

WEAU d20 0040 3 -----------.---------.-----------I----------------- 12 3 1 7 0.00051 0.00025 0.00017 0.00037

WEAU d20 0041 3 ---E-------.---------.----------------------------- 11 3 0 0 0.00047 0.00025 0.00000 0.00000

WEAU d20 0042 3 --F--------.---------.----------------------------- 7 3 2 0 0.00030 0.00025 0.00034 0.00000

WEAU d20 0043 3 -----------.---------.------------------T---------- 6 3 0 0 0.00026 0.00025 0.00000 0.00000

WEAU d20 0044 3 -----------.-----G---.----------------------------- 4 3 0 0 0.00017 0.00025 0.00000 0.00000

WEAU d20 0045 3 ----------T.---------.----------------------------- 6 3 0 11 0.00026 0.00025 0.00000 0.00058

WEAU d20 0046 3 -----------.---------.----------------L------------ 20 3 0 7 0.00085 0.00025 0.00000 0.00037

WEAU d20 0047 3 -----------.---V-----.----------------------------- 3 3 0 0 0.00013 0.00025 0.00000 0.00000

WEAU d20 0048 3 -----------.---------.--------------------V-------- 5 3 0 3 0.00021 0.00025 0.00000 0.00016

WEAU d20 0049 3 -----------.---------.----------A------------------ 1 3 0 1 0.00004 0.00025 0.00000 0.00005

WEAU d20 0051 3 ----------V.---------.----------------------------- 7 3 0 3 0.00030 0.00025 0.00000 0.00016

WEAU d20 0052 3 --H--------.---------.----------------------------- 0 3 0 0 0.00000 0.00025 0.00000 0.00000

WEAU d20 0053 3 -----------.---------.---------I------------N------ 0 3 0 0 0.00000 0.00025 0.00000 0.00000

WEAU d20 0054 3 -----------.---------.--------------Y-------------- 15 3 0 2 0.00064 0.00025 0.00000 0.00011

WEAU d20 0055 3 I----------.---------.----------------------------- 4 3 0 1 0.00017 0.00025 0.00000 0.00005

WEAU d20 0056 3 -----------.---------.-----------------------A----- 10 3 1 53 0.00043 0.00025 0.00017 0.00280

WEAU d20 0057 2 -----------.---------.----------------------------I 0 2 0 3 0.00000 0.00017 0.00000 0.00016

WEAU d20 0058 2 -----------.---------.---------N------------------- 0 2 4 0 0.00000 0.00017 0.00068 0.00000

WEAU d20 0061 2 -----------.---------.---------------------------S- 0 2 0 5 0.00000 0.00017 0.00000 0.00026

WEAU d20 0062 2 -----------.---------.--------------------T-------- 2 2 2 0 0.00009 0.00017 0.00034 0.00000

WEAU d20 0063 2 ----------P.---------.----------------------------- 0 2 0 0 0.00000 0.00017 0.00000 0.00000

WEAU d20 0064 2 -----------.---------.----------------P------------ 0 2 0 0 0.00000 0.00017 0.00000 0.00000

WEAU d20 0065 2 -----------.---------.----G------------------------ 5 2 0 0 0.00021 0.00017 0.00000 0.00000

WEAU d20 0066 2 -----------.---------.------------------V---------- 4 2 0 0 0.00017 0.00017 0.00000 0.00000

WEAU d20 0067 2 --------Y--.---------.----------------------------- 9 2 2 1 0.00038 0.00017 0.00034 0.00005

WEAU d20 0069 2 -----------.---------.-----R----------------------- 1 2 0 1 0.00004 0.00017 0.00000 0.00005

WEAU d20 0071 2 -----------.E--------.----------------------------- 0 2 0 3 0.00000 0.00017 0.00000 0.00016

WEAU d20 0072 2 -----------.---------.-----G----------------------- 2 2 0 0 0.00009 0.00017 0.00000 0.00000

WEAU d20 0073 2 -----------.----R----.----------------------------- 7 2 0 1 0.00030 0.00017 0.00000 0.00005

WEAU d20 0075 2 -----------.---------.----M------------------------ 1 2 0 4 0.00004 0.00017 0.00000 0.00021

WEAU d20 0077 2 -----W-----.---------.----------------------------- 0 2 0 3 0.00000 0.00017 0.00000 0.00016

WEAU d20 0078 2 -I---------.---------.----------------------------- 0 2 0 4 0.00000 0.00017 0.00000 0.00021

WEAU d20 0080 2 -----------.-V-------.----------------------------- 0 2 1 0 0.00000 0.00017 0.00017 0.00000

WEAU d20 0081 2 -----------.---------.--A-------------------------- 4 2 0 0 0.00017 0.00017 0.00000 0.00000

WEAU d20 0082 2 -----------.---------.--G-------------------------- 0 2 0 0 0.00000 0.00017 0.00000 0.00000

WEAU d20 0084 2 -----------.--------S.----------------------------- 0 2 0 0 0.00000 0.00017 0.00000 0.00000

WEAU d20 0085 2 -----------.---------.---L------------------------- 3 2 0 4 0.00013 0.00017 0.00000 0.00021

WEAU d20 0086 2 -----------.---------.-----------------G----------- 0 2 0 1 0.00000 0.00017 0.00000 0.00005

WEAU d20 0087 2 -----------.---------.-------------S--------------- 3 2 0 0 0.00013 0.00017 0.00000 0.00000

WEAU d20 0088 2 -------T---.---------.----------------------------- 0 2 2 5 0.00000 0.00017 0.00034 0.00026

WEAU d20 0089 2 -----------.---------.-----------------E----------- 0 2 4 0 0.00000 0.00017 0.00068 0.00000

WEAU d20 0090 2 ----V------.---------.----------------------------- 0 2 0 0 0.00000 0.00017 0.00000 0.00000

WEAU d20 0091 2 -----------.---------.-R--------------------------- 13 2 1 5 0.00055 0.00017 0.00017 0.00026

WEAU d20 0092 2 -----------.---------.--------T-------------------- 5 2 0 135 0.00021 0.00017 0.00000 0.00714

WEAU d20 0093 1 ------V----.---------.----------------------------- 1 1 1 0 0.00004 0.00008 0.00017 0.00000

WEAU d20 0094 1 -----------.---------.-----C----------------------- 3 1 0 1 0.00013 0.00008 0.00000 0.00005

WEAU d20 0095 1 -----------.---------.-------G--------------------- 3 1 0 3 0.00013 0.00008 0.00000 0.00016

WEAU d20 0098 1 -----------.---------.-------------------------G--- 0 1 0 1 0.00000 0.00008 0.00000 0.00005

WEAU d20 0102 1 -----------.---------.----------------------------F 0 1 0 3 0.00000 0.00008 0.00000 0.00016

WEAU d20 0105 1 -----------.-------D-.----------------------------- 1 1 0 0 0.00004 0.00008 0.00000 0.00000

WEAU d20 0106 1 --------S--.---------.----------------------------- 0 1 0 0 0.00000 0.00008 0.00000 0.00000

WEAU d20 0108 1 -----------.---------.--------------WT------------- 0 1 0 0 0.00000 0.00008 0.00000 0.00000

WEAU d20 0109 1 R----------.---------.----------------------------- 0 1 0 0 0.00000 0.00008 0.00000 0.00000

WEAU d20 0110 1 -----------.------I--.----------------------------- 6 1 0 2 0.00026 0.00008 0.00000 0.00011

WEAU d20 0111 1 ---------R-.---------.----------------------------- 0 1 0 4 0.00000 0.00008 0.00000 0.00021

WEAU d20 0114 1 -----------.---------.------R---------------------- 0 1 0 0 0.00000 0.00008 0.00000 0.00000

WEAU d20 0117 1 -----------.---------.-----------------------K----- 0 1 0 0 0.00000 0.00008 0.00000 0.00000

WEAU d20 0118 1 -----------.---------.----------------A------------ 3 1 0 0 0.00013 0.00008 0.00000 0.00000

WEAU d20 0122 1 -----------.---------.-----------------------I----- 4 1 0 1 0.00017 0.00008 0.00000 0.00005

WEAU d20 0126 1 ----------D.---------.----------------------------- 3 1 0 6 0.00013 0.00008 0.00000 0.00032

WEAU d20 0128 1 -----------.---------.D---------------------------- 0 1 0 0 0.00000 0.00008 0.00000 0.00000

WEAU d20 0129 1 -----------.---------.S---------------------------- 3 1 0 0 0.00013 0.00008 0.00000 0.00000

WEAU d20 0130 1 -----------.---------.---------------------------T- 0 1 0 0 0.00000 0.00008 0.00000 0.00000

WEAU d20 0131 1 -----------.---------.------------------G---------- 1 1 0 0 0.00004 0.00008 0.00000 0.00000

WEAU d20 0132 1 -----------.---------.--L-------------------------- 1 1 0 1 0.00004 0.00008 0.00000 0.00005

WEAU d20 0133 1 -----------.---------.-------------------------L--- 1 1 0 0 0.00004 0.00008 0.00000 0.00000

WEAU d20 0134 1 -----------.---------.-------------C--------------- 0 1 0 1 0.00000 0.00008 0.00000 0.00005

WEAU d20 0135 1 -----V-----.---------.----------------------------- 0 1 0 0 0.00000 0.00008 0.00000 0.00000

WEAU d20 0136 1 -----------.---------.-----------------A----------- 0 1 0 0 0.00000 0.00008 0.00000 0.00000

WEAU d20 0140 1 -F---------.---------.----------------------------- 2 1 0 4 0.00009 0.00008 0.00000 0.00021

WEAU d20 0145 1 -----------.---------.---------------------N------- 0 1 0 0 0.00000 0.00008 0.00000 0.00000

WEAU d20 0146 1 -----------.------S--.----------------------------- 1 1 0 0 0.00004 0.00008 0.00000 0.00000

WEAU d20 0147 1 L----------.---------.----------------------------- 0 1 0 1 0.00000 0.00008 0.00000 0.00005

WEAU d20 0149 1 -----------.---------.----------S------------------ 3 1 0 1 0.00013 0.00008 0.00000 0.00005

WEAU d20 0151 1 -----------.---------.----------------------G------ 0 1 0 1 0.00000 0.00008 0.00000 0.00005

WEAU d20 0152 1 --P--------.---------.----------------------------- 1 1 2 0 0.00004 0.00008 0.00034 0.00000

WEAU d20 0154 1 -----------.---------.----------N------------------ 0 1 0 1 0.00000 0.00008 0.00000 0.00005

WEAU d20 0155 1 -----------.---------.-------------L--------------- 1 1 0 0 0.00004 0.00008 0.00000 0.00000

WEAU d20 0156 1 ---V-------.---------.----------------------------- 1 1 0 1 0.00004 0.00008 0.00000 0.00005

WEAU d20 0157 1 ---------N-.---------.----------------------------- 2 1 0 13 0.00009 0.00008 0.00000 0.00069

WEAU d20 0159 1 -----------.---------.--------------R-------------- 2 1 0 0 0.00009 0.00008 0.00000 0.00000

WEAU d20 0160 1 -----------.---------.----------------L------A----- 0 1 0 0 0.00000 0.00008 0.00000 0.00000

WEAU d20 0162 1 -----S-----.---------.----------------------------- 0 1 0 0 0.00000 0.00008 0.00000 0.00000

WEAU d20 0165 1 -----------.---------.-------------------E--------- 0 1 0 0 0.00000 0.00008 0.00000 0.00000

WEAU d20 0168 1 -----W-----.---------.---------S------------------- 0 1 0 0 0.00000 0.00008 0.00000 0.00000

WEAU d20 0170 1 -----------.---------.--------------------------Q-- 0 1 0 0 0.00000 0.00008 0.00000 0.00000

WEAU d20 0173 1 -----------.---------.----------------------N------ 1 1 0 0 0.00004 0.00008 0.00000 0.00000

WEAU d20 0174 1 -----------.----S----.----------------------------- 0 1 0 0 0.00000 0.00008 0.00000 0.00000

WEAU d20 0175 1 -----------.---------.H---------------------------- 0 1 0 0 0.00000 0.00008 0.00000 0.00000

WEAU d20 0176 1 -----------.---F-----.----------------------------- 1 1 0 0 0.00004 0.00008 0.00000 0.00000

WEAU d20 0177 1 --R--------.---------.----------------------------- 1 1 0 0 0.00004 0.00008 0.00000 0.00000

WEAU d20 0178 1 -----------.---------.---------------------------H- 0 1 0 0 0.00000 0.00008 0.00000 0.00000

WEAU d20 0179 1 -------S---.---------.----------------------------- 0 1 1 0 0.00000 0.00008 0.00017 0.00000

WEAU d20 0180 1 -----------.---------.---------P------------------- 0 1 0 0 0.00000 0.00008 0.00000 0.00000

WEAU d20 0181 1 -----------.---------.----------------------------G 0 1 0 0 0.00000 0.00008 0.00000 0.00000

WEAU d20 0184 1 -----S-----.--------H.----------------------------- 0 1 0 0 0.00000 0.00008 0.00000 0.00000

WEAU d20 0187 1 -----------.---------.-------------------R--------- 1 1 0 0 0.00004 0.00008 0.00000 0.00000

WEAU d20 0188 1 -----------.---------.---------------------------D- 1 1 0 0 0.00004 0.00008 0.00000 0.00000

WEAU d20 0190 1 -----------.-----I---.----------------------------- 6 1 0 2 0.00026 0.00008 0.00000 0.00011

WEAU d20 0191 1 -----------.---------.---------S------------------- 0 1 0 0 0.00000 0.00008 0.00000 0.00000

WEAU d20 0192 1 -----------.---------.------------------------D---- 1 1 0 0 0.00004 0.00008 0.00000 0.00000

WEAU d20 0193 1 -----------.---------.---------------------------I- 0 1 0 0 0.00000 0.00008 0.00000 0.00000

WEAU d20 0194 1 -P---------.---------.----------------------------- 5 1 0 0 0.00021 0.00008 0.00000 0.00000

WEAU d20 0196 1 --------R--.---------.----------------------------- 3 1 0 2 0.00013 0.00008 0.00000 0.00011

WEAU d20 0204 1 -----------.------I--.-------------C--------------- 0 1 0 0 0.00000 0.00008 0.00000 0.00000

WEAU d20 0210 1 -----------.---------.----L------------------------ 1 1 0 1 0.00004 0.00008 0.00000 0.00005

WEAU d20 0213 1 -----------.---------.---------------------C------- 0 1 0 0 0.00000 0.00008 0.00000 0.00000

Other d30 sequences:

WEAU d30 0017 5 -----------.---------.---------------------H------- 11 7 5 3 0.00047 0.00059 0.00085 0.00016

WEAU d30 0018 4 -----------.---------.---------N------------------- 0 2 4 0 0.00000 0.00017 0.00068 0.00000

WEAU d30 0020 4 -----------.---------.-----------------E----------- 0 2 4 0 0.00000 0.00017 0.00068 0.00000

WEAU d30 0025 3 -----------.--T------.----------------------------- 0 4 3 0 0.00000 0.00034 0.00051 0.00000

WEAU d30 0031 2 --P--------.---------.----------------------------- 1 1 2 0 0.00004 0.00008 0.00034 0.00000

WEAU d30 0032 2 -----------.---------.---------I------------------- 10 5 2 11 0.00043 0.00042 0.00034 0.00058

WEAU d30 0033 2 -----------.-Q-------.----------------------------- 0 0 2 0 0.00000 0.00000 0.00034 0.00000

WEAU d30 0041 2 -----------.---------.--------------------T-------- 2 2 2 0 0.00009 0.00017 0.00034 0.00000

WEAU d30 0042 2 --F--------.---------.----------------------------- 7 3 2 0 0.00030 0.00025 0.00034 0.00000

WEAU d30 0043 2 --------Y--.---------.----------------------------- 9 2 2 1 0.00038 0.00017 0.00034 0.00005

WEAU d30 0044 2 -------T---.---------.----------------------------- 0 2 2 5 0.00000 0.00017 0.00034 0.00026

WEAU d30 0048 1 ------V----.---------.----------------------------- 1 1 1 0 0.00004 0.00008 0.00017 0.00000

WEAU d30 0054 1 -----------.---------.--------------------------Y-- 2 0 1 2 0.00009 0.00000 0.00017 0.00011

WEAU d30 0059 1 -----------.---------.-----------I----------------- 12 3 1 7 0.00051 0.00025 0.00017 0.00037

WEAU d30 0061 1 -----------.-V-------.----------------------------- 0 2 1 0 0.00000 0.00017 0.00017 0.00000

WEAU d30 0062 1 -------S---.---------.----------------------------- 0 1 1 0 0.00000 0.00008 0.00017 0.00000

WEAU d30 0063 1 -------V---.---------.----------------------------- 0 0 1 1 0.00000 0.00000 0.00017 0.00005

WEAU d30 0067 1 ------L----.---------.----------------------------- 0 0 1 0 0.00000 0.00000 0.00017 0.00000

WEAU d30 0072 1 -----------.P--------.----------------------------- 0 0 1 0 0.00000 0.00000 0.00017 0.00000

WEAU d30 0073 1 -----------.------A--.----------------------------- 2 7 1 1 0.00009 0.00059 0.00017 0.00005

WEAU d30 0082 1 -----------.---------.-R--------------------------- 13 2 1 5 0.00055 0.00017 0.00017 0.00026

WEAU d30 0083 1 -----------.---------.-----------------------A----- 10 3 1 53 0.00043 0.00025 0.00017 0.00280

Other RIER sequences:

RIER --- 0003 3645 -----------.---------.-------D--------------------- 0 0 0 3640 0.00000 0.00000 0.00000 0.19257

RIER --- 0004 2265 -----------.-DK------.----------------------------- 0 0 0 2265 0.00000 0.00000 0.00000 0.11982

RIER --- 0005 547 ----M------.---------.----------------------------- 0 0 0 547 0.00000 0.00000 0.00000 0.02894

RIER --- 0007 208 -----------.-DK------.-------D--------------------- 0 0 0 208 0.00000 0.00000 0.00000 0.01100

RIER --- 0008 147 -----------.-D-------.-------D--------------------- 0 0 0 147 0.00000 0.00000 0.00000 0.00778

RIER --- 0009 135 -----------.---------.--------T-------------------- 5 2 0 135 0.00021 0.00017 0.00000 0.00714

RIER --- 0010 108 ----M------.-DK------.----------------------------- 0 0 0 108 0.00000 0.00000 0.00000 0.00571

RIER --- 0011 68 ----M------.---------.-------D--------------------- 0 0 0 68 0.00000 0.00000 0.00000 0.00360

RIER --- 0013 53 -----------.---------.-----------------------A----- 10 3 1 53 0.00043 0.00025 0.00017 0.00280

RIER --- 0014 40 -----------.-D-------.--------T-------------------- 0 0 0 40 0.00000 0.00000 0.00000 0.00212

RIER --- 0015 35 -----------.-D-------.-----------------------A----- 0 0 0 35 0.00000 0.00000 0.00000 0.00185

RIER --- 0016 33 -----------.---------.-------D-A------------------- 0 0 0 33 0.00000 0.00000 0.00000 0.00175

RIER --- 0017 30 -----------.-G-------.-------D--------------------- 0 0 0 30 0.00000 0.00000 0.00000 0.00159

RIER --- 0018 21 ---------N-.-D-------.----------------------------- 0 0 0 21 0.00000 0.00000 0.00000 0.00111

RIER --- 0019 19 -----------.---------.-------------------------A--- 4 0 0 19 0.00017 0.00000 0.00000 0.00101

RIER --- 0020 17 ----M------.-D-------.----------------------------- 0 0 0 17 0.00000 0.00000 0.00000 0.00090

RIER --- 0021 16 ---------R-.-D-------.----------------------------- 0 0 0 16 0.00000 0.00000 0.00000 0.00085

RIER --- 0022 14 -----------.-DK------.--------T-------------------- 0 0 0 14 0.00000 0.00000 0.00000 0.00074

RIER --- 0023 14 ----------T.-DK------.----------------------------- 0 0 0 14 0.00000 0.00000 0.00000 0.00074

RIER --- 0025 13 -----------.---------.-------------------------I--- 1 0 0 13 0.00004 0.00000 0.00000 0.00069

RIER --- 0026 13 -----------.-D-------.---------I------------------- 0 0 0 13 0.00000 0.00000 0.00000 0.00069

RIER --- 0027 13 ---------N-.---------.----------------------------- 2 1 0 13 0.00009 0.00008 0.00000 0.00069

RIER --- 0028 12 ---------N-.-DK------.----------------------------- 0 0 0 12 0.00000 0.00000 0.00000 0.00063

RIER --- 0029 12 -----------.TD-------.----------------------------- 0 0 0 12 0.00000 0.00000 0.00000 0.00063

RIER --- 0030 11 ----------T.---------.----------------------------- 6 3 0 11 0.00026 0.00025 0.00000 0.00058

RIER --- 0031 11 -----------.---------.---------I------------------- 10 5 2 11 0.00043 0.00042 0.00034 0.00058

RIER --- 0032 11 ----M------.---------.--------T-------------------- 0 0 0 11 0.00000 0.00000 0.00000 0.00058

RIER --- 0033 11 --------S--.---------.-------D--------------------- 0 0 0 11 0.00000 0.00000 0.00000 0.00058

RIER --- 0034 10 -----------.---------.-------D---------------A----- 0 0 0 10 0.00000 0.00000 0.00000 0.00053

RIER --- 0035 10 -----------.---------.-----------------N----------- 1 5 0 10 0.00004 0.00042 0.00000 0.00053

RIER --- 0036 10 ---------N-.---------.-------D--------------------- 0 0 0 10 0.00000 0.00000 0.00000 0.00053

RIER --- 0037 9 -----------.T--------.-------D--------------------- 0 1 0 9 0.00000 0.00008 0.00000 0.00048

RIER --- 0038 9 -----------.-------I-.-------D--------------------- 0 0 0 9 0.00000 0.00000 0.00000 0.00048

RIER --- 0039 9 ----M------.--K------.----------------------------- 0 0 0 9 0.00000 0.00000 0.00000 0.00048

RIER --- 0040 9 -----------.---------.-------DT-------------------- 0 0 0 9 0.00000 0.00000 0.00000 0.00048

RIER --- 0041 9 -----------.---------.-------DS-------------------- 0 0 0 9 0.00000 0.00000 0.00000 0.00048

RIER --- 0042 9 -----------.-G-------.--------------------T-------- 0 0 0 9 0.00000 0.00000 0.00000 0.00048

RIER --- 0043 8 -----------.-N-------.----------------------------- 0 0 0 8 0.00000 0.00000 0.00000 0.00042

RIER --- 0044 8 V----------.-D-------.----------------------------- 0 0 0 8 0.00000 0.00000 0.00000 0.00042

RIER --- 0045 8 ----T------.---------.-------D--------------------- 0 0 0 8 0.00000 0.00000 0.00000 0.00042

RIER --- 0046 8 -----------.-DK------.---------------------H------- 0 0 0 8 0.00000 0.00000 0.00000 0.00042

RIER --- 0047 8 -----------.---------.--I----D--------------------- 0 0 0 8 0.00000 0.00000 0.00000 0.00042

RIER --- 0048 7 -----------.---------.-----------I----------------- 12 3 1 7 0.00051 0.00025 0.00017 0.00037

RIER --- 0050 7 ----T------.---------.----------------------------- 0 0 0 7 0.00000 0.00000 0.00000 0.00037

RIER --- 0051 7 -----------.---------.-------D---------N----------- 0 0 0 7 0.00000 0.00000 0.00000 0.00037

RIER --- 0052 7 -----------.---------.----------------L------------ 20 3 0 7 0.00085 0.00025 0.00000 0.00037

RIER --- 0053 7 ----------T.-D-------.----------------------------- 0 0 0 7 0.00000 0.00000 0.00000 0.00037

RIER --- 0054 7 -----------.-G-------.-----------------------A----- 0 0 1 7 0.00000 0.00000 0.00017 0.00037

RIER --- 0056 6 -----------.-D-------.-------D-A------------------- 0 0 0 6 0.00000 0.00000 0.00000 0.00032

RIER --- 0058 6 -----------.-DK------.--I-------------------------- 0 0 0 6 0.00000 0.00000 0.00000 0.00032

RIER --- 0059 6 ------T----.-D-------.----------------------------- 0 0 0 6 0.00000 0.00000 0.00000 0.00032

RIER --- 0060 6 -----------.-D-------.-----------------N----------- 0 0 0 6 0.00000 0.00000 0.00000 0.00032

RIER --- 0061 6 ----------D.---------.----------------------------- 3 1 0 6 0.00013 0.00008 0.00000 0.00032

RIER --- 0062 6 --F--------.-D-------.-------D--------------------- 0 0 0 6 0.00000 0.00000 0.00000 0.00032

RIER --- 0063 6 -----------.-G-------.----------------------A------ 0 0 0 6 0.00000 0.00000 0.00000 0.00032

RIER --- 0064 5 -----------.-DK------.---------I------------------- 0 0 0 5 0.00000 0.00000 0.00000 0.00026

RIER --- 0065 5 -----------.---------.---------------------------S- 0 2 0 5 0.00000 0.00017 0.00000 0.00026

RIER --- 0066 5 -----------.---------.-------D--------------Y------ 0 0 0 5 0.00000 0.00000 0.00000 0.00026

RIER --- 0067 5 -----------.-DK------.-------D-A------------------- 0 0 0 5 0.00000 0.00000 0.00000 0.00026

RIER --- 0068 5 -----------.---------.----------------------E------ 0 0 0 5 0.00000 0.00000 0.00000 0.00026

RIER --- 0069 5 -----------.-DK------.-----------------------A----- 0 0 0 5 0.00000 0.00000 0.00000 0.00026

RIER --- 0070 5 T----------.---------.----------------------------- 0 0 0 5 0.00000 0.00000 0.00000 0.00026

RIER --- 0071 5 --------Y--.-D-------.----------------------------- 0 0 0 5 0.00000 0.00000 0.00000 0.00026

RIER --- 0072 5 -----------.-D-------.----------------------------I 0 0 0 5 0.00000 0.00000 0.00000 0.00026

RIER --- 0073 5 -------T---.---------.----------------------------- 0 2 2 5 0.00000 0.00017 0.00034 0.00026

RIER --- 0074 5 -----F-----.---------.-------D-A------------------- 0 0 0 5 0.00000 0.00000 0.00000 0.00026

RIER --- 0075 5 -----------.---------.-------D-------------H------- 0 0 0 5 0.00000 0.00000 0.00000 0.00026

RIER --- 0076 5 -----------.---------.-R--------------------------- 13 2 1 5 0.00055 0.00017 0.00017 0.00026

RIER --- 0077 5 ----M------.-DK------.-------D--------------------- 0 0 0 5 0.00000 0.00000 0.00000 0.00026

RIER --- 0078 4 ----M------.---------.--------T----------------A--- 0 0 0 4 0.00000 0.00000 0.00000 0.00021

RIER --- 0079 4 ----M------.---------.-------D---------N----------- 0 0 0 4 0.00000 0.00000 0.00000 0.00021

RIER --- 0080 4 ------I----.---------.-------D--------------------- 0 0 0 4 0.00000 0.00000 0.00000 0.00021

RIER --- 0081 4 -----------.-D-------.----------------------G------ 0 0 0 4 0.00000 0.00000 0.00000 0.00021

RIER --- 0082 4 -----------.-G-------.--I-------------------------- 0 0 0 4 0.00000 0.00000 0.00000 0.00021

RIER --- 0083 4 -----------.---------.---S---D--------------------- 0 0 0 4 0.00000 0.00000 0.00000 0.00021

RIER --- 0084 4 -I---------.---------.----------------------------- 0 2 0 4 0.00000 0.00017 0.00000 0.00021

RIER --- 0085 4 ----M------.---------.-------DT-------------------- 0 0 0 4 0.00000 0.00000 0.00000 0.00021

RIER --- 0086 4 -----------.---------.---L------------------------- 3 2 0 4 0.00013 0.00017 0.00000 0.00021

RIER --- 0088 4 --I--------.---------.----------------------------- 0 0 0 4 0.00000 0.00000 0.00000 0.00021

RIER --- 0090 4 -------T---.---------.-------D--------------------- 0 0 0 4 0.00000 0.00000 0.00000 0.00021

RIER --- 0091 4 -----------.---------.-------D-----------------I--- 0 0 0 4 0.00000 0.00000 0.00000 0.00021

RIER --- 0092 4 ----------V.-DK------.----------------------------- 0 0 0 4 0.00000 0.00000 0.00000 0.00021

RIER --- 0093 4 ---------R-.---------.----------------------------- 0 1 0 4 0.00000 0.00008 0.00000 0.00021

RIER --- 0094 4 -----------.---------.-R-----D--------------------- 0 0 0 4 0.00000 0.00000 0.00000 0.00021

RIER --- 0095 4 --------G--.---------.----------------------------- 1 0 0 4 0.00004 0.00000 0.00000 0.00021

RIER --- 0096 4 -----------.---------.-------D--------------E------ 0 0 0 4 0.00000 0.00000 0.00000 0.00021

RIER --- 0097 4 -F---------.---------.----------------------------- 2 1 0 4 0.00009 0.00008 0.00000 0.00021

RIER --- 0098 4 -----------.---------.----M------------------------ 1 2 0 4 0.00004 0.00017 0.00000 0.00021

RIER --- 0099 4 -----------.--K------.-------D--------------------- 0 0 0 4 0.00000 0.00000 0.00000 0.00021

RIER --- 0100 3 -----------.---------.-------G--------------------- 3 1 0 3 0.00013 0.00008 0.00000 0.00016

RIER --- 0101 3 -----------.---------.----------------------------F 0 1 0 3 0.00000 0.00008 0.00000 0.00016

RIER --- 0102 3 ----M------.-DK------.---------A------------------- 0 0 0 3 0.00000 0.00000 0.00000 0.00016

RIER --- 0103 3 -----------.---------.----------------------------I 0 2 0 3 0.00000 0.00017 0.00000 0.00016

RIER --- 0104 3 -----------.---------.------E---------------------- 7 5 0 3 0.00030 0.00042 0.00000 0.00016

RIER --- 0106 3 ----M------.---------.-----------------N----------- 0 0 0 3 0.00000 0.00000 0.00000 0.00016

RIER --- 0107 3 -----------.VD-------.----------------------------- 0 0 0 3 0.00000 0.00000 0.00000 0.00016

RIER --- 0108 3 -----------.-DK------.-----------------N----------- 0 0 0 3 0.00000 0.00000 0.00000 0.00016

RIER --- 0109 3 -----------.-D-------.----------------------A------ 0 0 0 3 0.00000 0.00000 0.00000 0.00016

RIER --- 0110 3 -----------.-DK------.--------------------T-------- 0 0 0 3 0.00000 0.00000 0.00000 0.00016

RIER --- 0111 3 ----------V.---------.----------------------------- 7 3 0 3 0.00030 0.00025 0.00000 0.00016

RIER --- 0112 3 -----------.---------.---------------------H------- 11 7 5 3 0.00047 0.00059 0.00085 0.00016

RIER --- 0113 3 -----------.---------.-------K--------------------- 4 5 0 3 0.00017 0.00042 0.00000 0.00016

RIER --- 0114 3 -----------.---------.-------D-I------------------- 0 0 0 3 0.00000 0.00000 0.00000 0.00016

RIER --- 0115 3 ----M------.-DK------.---------I------------------- 0 0 0 3 0.00000 0.00000 0.00000 0.00016

RIER --- 0116 3 -----------.---------.----------------------Y------ 2 0 0 3 0.00009 0.00000 0.00000 0.00016

RIER --- 0117 3 I----------.-D-------.----------------------------- 0 0 0 3 0.00000 0.00000 0.00000 0.00016

RIER --- 0118 3 -----------.-DK------.----------------------E------ 0 0 0 3 0.00000 0.00000 0.00000 0.00016

RIER --- 0119 3 -----------.-D-------.-----------P----------------- 0 0 0 3 0.00000 0.00000 0.00000 0.00016

RIER --- 0120 3 -----------.-D-------.----G------------------------ 0 0 0 3 0.00000 0.00000 0.00000 0.00016

RIER --- 0121 3 -----------.---------.-------D--------T------------ 0 0 0 3 0.00000 0.00000 0.00000 0.00016

RIER --- 0122 3 -----W-----.---------.-------D--------------------- 0 0 0 3 0.00000 0.00000 0.00000 0.00016

RIER --- 0123 3 -----------.E--------.----------------------------- 0 2 0 3 0.00000 0.00017 0.00000 0.00016

RIER --- 0124 3 -----------.-D-------.---------------------------K- 0 0 0 3 0.00000 0.00000 0.00000 0.00016

RIER --- 0125 3 -----------.---------.--------------------V-------- 5 3 0 3 0.00021 0.00025 0.00000 0.00016

RIER --- 0126 3 -----W-----.---------.----------------------------- 0 2 0 3 0.00000 0.00017 0.00000 0.00016

RIER --- 0127 3 -----------.-DK------.---------------V------------- 0 0 0 3 0.00000 0.00000 0.00000 0.00016

RIER --- 0128 3 -----------.-DK------.-------D-N------------------- 0 0 0 3 0.00000 0.00000 0.00000 0.00016

RIER --- 0129 3 -----S-----.-D-------.----------------------------- 0 0 0 3 0.00000 0.00000 0.00000 0.00016

RIER --- 0130 2 ----T-----T.---------.----------------------------- 0 0 0 2 0.00000 0.00000 0.00000 0.00011

RIER --- 0131 2 ---------G-.-D-------.----------------------------- 0 0 0 2 0.00000 0.00000 0.00000 0.00011

RIER --- 0132 2 -----------.---------.--------------------------Y-- 2 0 1 2 0.00009 0.00000 0.00017 0.00011

RIER --- 0133 2 -----------.---------.-------DV-------------------- 0 0 0 2 0.00000 0.00000 0.00000 0.00011

RIER --- 0134 2 ----M------.-G-------.------R---------------------- 0 0 0 2 0.00000 0.00000 0.00000 0.00011

RIER --- 0135 2 -----------.-DK------.-------------------------A--- 0 0 0 2 0.00000 0.00000 0.00000 0.00011

RIER --- 0136 2 -----------.---------.-------D--I------------------ 0 0 0 2 0.00000 0.00000 0.00000 0.00011

RIER --- 0137 2 -----------.-D-------.-------------------------A--- 0 0 0 2 0.00000 0.00000 0.00000 0.00011

RIER --- 0138 2 -----------.---------.---------------V------------- 9 6 0 2 0.00038 0.00051 0.00000 0.00011

RIER --- 0140 2 ------R----.---------.----------------------------- 0 0 0 2 0.00000 0.00000 0.00000 0.00011

RIER --- 0142 2 -----S-----.---------.-------D--------------------- 0 0 0 2 0.00000 0.00000 0.00000 0.00011

RIER --- 0143 2 ----M------.---------.-----------------------A----- 0 0 0 2 0.00000 0.00000 0.00000 0.00011

RIER --- 0144 2 -----------.---------.-------D-----------------A--- 0 0 0 2 0.00000 0.00000 0.00000 0.00011

RIER --- 0145 2 -F---------.-D-------.----------------------------- 0 0 0 2 0.00000 0.00000 0.00000 0.00011

RIER --- 0146 2 ----------T.-DK------.H---------------------------- 0 0 0 2 0.00000 0.00000 0.00000 0.00011

RIER --- 0148 2 -----------.-D-------.-------------S--------------- 0 0 0 2 0.00000 0.00000 0.00000 0.00011

RIER --- 0149 2 -----------.-D-------.--------V-------------------- 0 0 0 2 0.00000 0.00000 0.00000 0.00011

RIER --- 0150 2 -----------.---------.-------D------------------Y-- 0 0 0 2 0.00000 0.00000 0.00000 0.00011

RIER --- 0151 2 -----------.-D-------.------R---------------------- 0 0 0 2 0.00000 0.00000 0.00000 0.00011

RIER --- 0152 2 -----------.-DK------.--------------------V-------- 0 0 0 2 0.00000 0.00000 0.00000 0.00011

RIER --- 0153 2 I----------.-DK------.----------------------------- 0 0 0 2 0.00000 0.00000 0.00000 0.00011

RIER --- 0154 2 -----------.-DK------.-------D-----------------A--- 0 0 0 2 0.00000 0.00000 0.00000 0.00011

RIER --- 0155 2 -----------.-K-------.-------D--------------------- 0 0 0 2 0.00000 0.00000 0.00000 0.00011

RIER --- 0156 2 -----------.---------.--I-------------------------- 8 4 0 2 0.00034 0.00034 0.00000 0.00011

RIER --- 0157 2 -F---------.-DK------.----------------------------- 0 0 0 2 0.00000 0.00000 0.00000 0.00011

RIER --- 0158 2 -----------.---------.-R--------------------Y------ 0 0 0 2 0.00000 0.00000 0.00000 0.00011

RIER --- 0159 2 -----------.---------.--------------V-------------- 0 0 0 2 0.00000 0.00000 0.00000 0.00011

RIER --- 0160 2 -----------.-D-------.-----L----------------------- 0 0 0 2 0.00000 0.00000 0.00000 0.00011

RIER --- 0161 2 ----T------.-D-------.----------------------------- 0 0 0 2 0.00000 0.00000 0.00000 0.00011

RIER --- 0163 2 -----------.-DK------.---------A------------------- 0 0 0 2 0.00000 0.00000 0.00000 0.00011

RIER --- 0164 2 -----------.---------.-------D-AA------------------ 0 0 0 2 0.00000 0.00000 0.00000 0.00011

RIER --- 0165 2 -----------.------I--.----------------------------- 6 1 0 2 0.00026 0.00008 0.00000 0.00011

RIER --- 0166 2 -----------.---------.---S------------------------- 7 6 0 2 0.00030 0.00051 0.00000 0.00011

RIER --- 0167 2 -------S---.-DK------.----------------------------- 0 0 0 2 0.00000 0.00000 0.00000 0.00011

RIER --- 0168 2 -----------.-D-------.---------------------------S- 0 0 0 2 0.00000 0.00000 0.00000 0.00011

RIER --- 0169 2 ----------V.-D-------.----------------------------- 0 0 0 2 0.00000 0.00000 0.00000 0.00011

RIER --- 0170 2 -----------.--------F.----------------------------- 0 0 0 2 0.00000 0.00000 0.00000 0.00011

RIER --- 0171 2 -P---------.-D-------.----------------------------- 0 0 0 2 0.00000 0.00000 0.00000 0.00011

RIER --- 0173 2 -----------.---------.--------V-------------------- 5 4 0 2 0.00021 0.00034 0.00000 0.00011

RIER --- 0174 2 -----------.-D-------.----------------------Y------ 0 0 0 2 0.00000 0.00000 0.00000 0.00011

RIER --- 0175 2 -----------.---------.------------------S---------- 1 0 0 2 0.00004 0.00000 0.00000 0.00011

RIER --- 0177 2 -----------.-DK--A---.----------------------------- 0 0 0 2 0.00000 0.00000 0.00000 0.00011

RIER --- 0178 2 -----------.-DK------.-----------------G----------- 0 0 0 2 0.00000 0.00000 0.00000 0.00011

RIER --- 0179 2 ---------R-.-D-------.-------D--------------------- 0 0 0 2 0.00000 0.00000 0.00000 0.00011

RIER --- 0180 2 -----------.-D-V-----.----------------------------- 0 0 0 2 0.00000 0.00000 0.00000 0.00011

RIER --- 0181 2 -----------.---------.-------N--------------------- 0 0 0 2 0.00000 0.00000 0.00000 0.00011

RIER --- 0182 2 --------G--.---------.-------D--------------------- 0 0 0 2 0.00000 0.00000 0.00000 0.00011

RIER --- 0183 2 -----------.---------.-------D-------V------------- 0 0 0 2 0.00000 0.00000 0.00000 0.00011

RIER --- 0184 2 ---V-------.-D-------.----------------------------- 0 0 0 2 0.00000 0.00000 0.00000 0.00011

RIER --- 0185 2 ----M------.T--------.----------------------------- 0 0 0 2 0.00000 0.00000 0.00000 0.00011

RIER --- 0186 2 ---------N-.-D-------.-------D--------------------- 0 0 0 2 0.00000 0.00000 0.00000 0.00011

RIER --- 0187 2 -----------.-----I---.----------------------------- 6 1 0 2 0.00026 0.00008 0.00000 0.00011

RIER --- 0188 2 --------R--.---------.----------------------------- 3 1 0 2 0.00013 0.00008 0.00000 0.00011

RIER --- 0189 2 ---------R-.-A-------.----------------------------- 0 0 0 2 0.00000 0.00000 0.00000 0.00011

RIER --- 0190 2 -----------.-DK-----F.----------------------------- 0 0 0 2 0.00000 0.00000 0.00000 0.00011

RIER --- 0191 2 -----------.---------.--------------Y-------------- 15 3 0 2 0.00064 0.00025 0.00000 0.00011

RIER --- 0192 2 -----------.-G-------.----------------------------I 0 0 0 2 0.00000 0.00000 0.00000 0.00011

RIER --- 0195 1 -----------.-DK------.-----------------------I----- 0 0 0 1 0.00000 0.00000 0.00000 0.00005

RIER --- 0196 1 -----------.---------.-V--------------------------- 0 0 0 1 0.00000 0.00000 0.00000 0.00005

RIER --- 0197 1 -----------.-D-------.----------I------------------ 0 0 0 1 0.00000 0.00000 0.00000 0.00005

RIER --- 0198 1 -----------.-D-------.-------D--------L------------ 0 0 0 1 0.00000 0.00000 0.00000 0.00005

RIER --- 0199 1 -----------.---------.-------D-----C--------------- 0 0 0 1 0.00000 0.00000 0.00000 0.00005

RIER --- 0200 1 ---------N-.-D-------.--------T-------------------- 0 0 0 1 0.00000 0.00000 0.00000 0.00005

RIER --- 0201 1 -----------.-D-------.---------------T------------- 0 0 0 1 0.00000 0.00000 0.00000 0.00005

RIER --- 0202 1 -----------.---------.-------------------------G--- 0 1 0 1 0.00000 0.00008 0.00000 0.00005

RIER --- 0203 1 V----------.---------.-------D--------------------- 0 0 0 1 0.00000 0.00000 0.00000 0.00005

RIER --- 0204 1 ---------G-.-DK------.----------------------------- 0 0 0 1 0.00000 0.00000 0.00000 0.00005

RIER --- 0205 1 -----------.-G-------.-------D-N------------------- 0 0 0 1 0.00000 0.00000 0.00000 0.00005

RIER --- 0206 1 ------V----.---------.-------D--------------------- 0 0 0 1 0.00000 0.00000 0.00000 0.00005

RIER --- 0207 1 -----------.------I--.-------D--------------------- 0 0 0 1 0.00000 0.00000 0.00000 0.00005

RIER --- 0208 1 --F--------.-D-------.-------------------------A--- 0 0 0 1 0.00000 0.00000 0.00000 0.00005

RIER --- 0209 1 ------L----.---------.-------D--------------------- 0 0 0 1 0.00000 0.00000 0.00000 0.00005

RIER --- 0210 1 --------Y--.-DK------.----------------------------- 0 0 0 1 0.00000 0.00000 0.00000 0.00005

RIER --- 0211 1 -----------.-DK---A--.-------D--------------------- 0 0 0 1 0.00000 0.00000 0.00000 0.00005

RIER --- 0212 1 ----M------.---------.----------------------E------ 0 0 0 1 0.00000 0.00000 0.00000 0.00005

RIER --- 0214 1 -----------.-D-------.------------------------G---- 0 0 0 1 0.00000 0.00000 0.00000 0.00005

RIER --- 0215 1 -----------.---------.-------D--------------------A 0 0 0 1 0.00000 0.00000 0.00000 0.00005

RIER --- 0216 1 -----------.---------.---------A------------------- 2 0 0 1 0.00009 0.00000 0.00000 0.00005

RIER --- 0217 1 -----------.--K------.-----------------------A----- 0 0 1 1 0.00000 0.00000 0.00017 0.00005

RIER --- 0218 1 -----------.-DK------.---L------------------------- 0 0 0 1 0.00000 0.00000 0.00000 0.00005

RIER --- 0219 1 ------V----.-D-------.----------------------------- 0 0 0 1 0.00000 0.00000 0.00000 0.00005

RIER --- 0220 1 -----------.-D-------.----------------P------------ 0 0 0 1 0.00000 0.00000 0.00000 0.00005

RIER --- 0221 1 -----------.---------.-------D-------------------K- 0 0 0 1 0.00000 0.00000 0.00000 0.00005

RIER --- 0222 1 ------R----.---------.-------D-A------------------- 0 0 0 1 0.00000 0.00000 0.00000 0.00005

RIER --- 0223 1 -----------.---------.-----------------------I----- 4 1 0 1 0.00017 0.00008 0.00000 0.00005

RIER --- 0224 1 -----------.---------.-------D----------------D---- 0 0 0 1 0.00000 0.00000 0.00000 0.00005

RIER --- 0225 1 -----------.-DK------.---S------------------------- 0 0 0 1 0.00000 0.00000 0.00000 0.00005

RIER --- 0226 1 -----------.-D-------.---S------------------------- 0 0 0 1 0.00000 0.00000 0.00000 0.00005

RIER --- 0227 1 -----------.-D---F---.----------------------------- 0 0 0 1 0.00000 0.00000 0.00000 0.00005

RIER --- 0228 1 -----------.-D-------.---------------S------------- 0 0 0 1 0.00000 0.00000 0.00000 0.00005

RIER --- 0229 1 -----------.---------.------------------------A---- 0 0 0 1 0.00000 0.00000 0.00000 0.00005

RIER --- 0231 1 -----------.---------.--------------R--------A----- 0 0 0 1 0.00000 0.00000 0.00000 0.00005

RIER --- 0232 1 -----------.-D-------.--------------R--------A----- 0 0 0 1 0.00000 0.00000 0.00000 0.00005

RIER --- 0234 1 -----------.-DK------.-------------------------I--- 0 0 0 1 0.00000 0.00000 0.00000 0.00005

RIER --- 0235 1 -----------.---------.-------------C--------------- 0 1 0 1 0.00000 0.00008 0.00000 0.00005

RIER --- 0236 1 -----------.-D-------.---------A------------------- 0 0 0 1 0.00000 0.00000 0.00000 0.00005

RIER --- 0237 1 -----------.-DK------.----A------------------------ 0 0 0 1 0.00000 0.00000 0.00000 0.00005

RIER --- 0238 1 -----------.-NK------.-------D--------------------- 0 0 0 1 0.00000 0.00000 0.00000 0.00005

RIER --- 0239 1 ----M------.-KK------.----------------------------- 0 0 0 1 0.00000 0.00000 0.00000 0.00005

RIER --- 0240 1 -----------.-NK------.-------D--------L------------ 0 0 0 1 0.00000 0.00000 0.00000 0.00005

RIER --- 0241 1 -----------.---------.---------------------------KF 0 0 0 1 0.00000 0.00000 0.00000 0.00005

RIER --- 0242 1 -----------.---------.--------------------S-------- 6 0 0 1 0.00026 0.00000 0.00000 0.00005

RIER --- 0243 1 -----------.-D-------.------E---------------------- 0 0 0 1 0.00000 0.00000 0.00000 0.00005

RIER --- 0244 1 --------Y--.---------.----------------------------- 9 2 2 1 0.00038 0.00017 0.00034 0.00005

RIER --- 0245 1 ---E-------.-G-------.----------------------------- 0 0 0 1 0.00000 0.00000 0.00000 0.00005

RIER --- 0246 1 -----------.-D-------.-R--------------------------- 0 0 0 1 0.00000 0.00000 0.00000 0.00005

RIER --- 0247 1 ---R------P.-D-------.----------------------------- 0 0 0 1 0.00000 0.00000 0.00000 0.00005

RIER --- 0248 1 -----------.-D-------.-------D----P---------------- 0 0 0 1 0.00000 0.00000 0.00000 0.00005

RIER --- 0249 1 -----------.-D---I---.----------------------------- 0 0 0 1 0.00000 0.00000 0.00000 0.00005

RIER --- 0251 1 -----------.-D-------.-------V--------------------- 0 0 0 1 0.00000 0.00000 0.00000 0.00005

RIER --- 0253 1 -----------.---------.----------S------------------ 3 1 0 1 0.00013 0.00008 0.00000 0.00005

RIER --- 0254 1 ----M------.-K-------.----------------------------- 0 0 0 1 0.00000 0.00000 0.00000 0.00005

RIER --- 0255 1 -----------.---------.----------------------G------ 0 1 0 1 0.00000 0.00008 0.00000 0.00005

RIER --- 0256 1 -----------.---------.----------N------------------ 0 1 0 1 0.00000 0.00008 0.00000 0.00005

RIER --- 0257 1 -----------.----R----.----------------------------- 7 2 0 1 0.00030 0.00017 0.00000 0.00005

RIER --- 0258 1 -----------.-D-------.-----------------------I----- 0 0 0 1 0.00000 0.00000 0.00000 0.00005

RIER --- 0259 1 -----------.---------.-------D-------------------S- 0 0 0 1 0.00000 0.00000 0.00000 0.00005

RIER --- 0260 1 ----M------.---------.----G---T-------------------- 0 0 0 1 0.00000 0.00000 0.00000 0.00005

RIER --- 0261 1 -----------.-----A---.-------D--------------------- 0 0 0 1 0.00000 0.00000 0.00000 0.00005

RIER --- 0262 1 I---M------.-DK------.----------------------------- 0 0 0 1 0.00000 0.00000 0.00000 0.00005

RIER --- 0263 1 -F---------.---------.-------------------------I--- 0 0 0 1 0.00000 0.00000 0.00000 0.00005

RIER --- 0264 1 ------T----.---------.----------------------------- 0 0 0 1 0.00000 0.00000 0.00000 0.00005

RIER --- 0265 1 ---E-------.---------.-------D--------------------- 0 0 0 1 0.00000 0.00000 0.00000 0.00005

RIER --- 0266 1 -----------.---------.----------A------------------ 1 3 0 1 0.00004 0.00025 0.00000 0.00005

RIER --- 0267 1 -----------.--I------.----------------------------- 0 0 0 1 0.00000 0.00000 0.00000 0.00005

RIER --- 0268 1 -----------.-DK------.-------DS-------------------- 0 0 0 1 0.00000 0.00000 0.00000 0.00005

RIER --- 0269 1 -P---W-----.---------.-------D--------------------- 0 0 0 1 0.00000 0.00000 0.00000 0.00005

RIER --- 0270 1 -----------.---------.-------D--------------------G 0 0 0 1 0.00000 0.00000 0.00000 0.00005

RIER --- 0271 1 -----------.-------A-.----------------------------- 5 4 0 1 0.00021 0.00034 0.00000 0.00005

RIER --- 0272 1 ----M------.---------.-------------------R---A----- 0 0 0 1 0.00000 0.00000 0.00000 0.00005

RIER --- 0273 1 -----------.---------.-------DR-------------------- 0 0 0 1 0.00000 0.00000 0.00000 0.00005

RIER --- 0274 1 -----------.---------.---A------------------------- 0 0 0 1 0.00000 0.00000 0.00000 0.00005

RIER --- 0275 1 -----------.---------.-------D--A------------------ 0 0 0 1 0.00000 0.00000 0.00000 0.00005

RIER --- 0276 1 -----------.-DK--L---.----------------------------- 0 0 0 1 0.00000 0.00000 0.00000 0.00005

RIER --- 0277 1 V----------.-D-------.-------D--------------------- 0 0 0 1 0.00000 0.00000 0.00000 0.00005

RIER --- 0278 1 ----T------.-DK------.-------D--------------------- 0 0 0 1 0.00000 0.00000 0.00000 0.00005

RIER --- 0282 1 -----------.-D-------.-------------------T--------- 0 0 0 1 0.00000 0.00000 0.00000 0.00005

RIER --- 0283 1 -----------.---------.---------------S------------- 0 0 0 1 0.00000 0.00000 0.00000 0.00005

RIER --- 0284 1 -----------.---------.-------DV---I---------------- 0 0 0 1 0.00000 0.00000 0.00000 0.00005

RIER --- 0285 1 -----------.-D-------.-V--------------------------- 0 0 0 1 0.00000 0.00000 0.00000 0.00005

RIER --- 0286 1 -----------.-G-------.----------------------E------ 0 0 0 1 0.00000 0.00000 0.00000 0.00005

RIER --- 0287 1 -----------.-D-------.-------D-A---------------A--- 0 0 0 1 0.00000 0.00000 0.00000 0.00005

RIER --- 0288 1 -----------.-DK------.C------------------------A--- 0 0 0 1 0.00000 0.00000 0.00000 0.00005

RIER --- 0289 1 ----M------.-DK------.-------DT-------------------- 0 0 0 1 0.00000 0.00000 0.00000 0.00005

RIER --- 0290 1 -------M---.---------.-------D--------------------- 0 0 0 1 0.00000 0.00000 0.00000 0.00005

RIER --- 0291 1 -----------.-D-------.-------D-AA------------------ 0 0 0 1 0.00000 0.00000 0.00000 0.00005

RIER --- 0292 1 -----------.-D-------.--------------------------Y-- 0 0 0 1 0.00000 0.00000 0.00000 0.00005

RIER --- 0293 1 -----------.---------.----L------------------------ 1 1 0 1 0.00004 0.00008 0.00000 0.00005

RIER --- 0294 1 -----------.-D--L----.----------------------------- 0 0 0 1 0.00000 0.00000 0.00000 0.00005

RIER --- 0295 1 -----------.---------.-----C----------------------- 3 1 0 1 0.00013 0.00008 0.00000 0.00005

RIER --- 0296 1 ---E-------.-D-------.----------------------------- 0 0 0 1 0.00000 0.00000 0.00000 0.00005

RIER --- 0297 1 -----------.---------.------RD--------------------- 0 0 0 1 0.00000 0.00000 0.00000 0.00005

RIER --- 0298 1 -----------.-D-------.----A------------------------ 0 0 0 1 0.00000 0.00000 0.00000 0.00005

RIER --- 0299 1 -----W-----.-DK------.----------------------------- 0 0 0 1 0.00000 0.00000 0.00000 0.00005

RIER --- 0300 1 -----------.VDK------.----------------------------- 0 0 0 1 0.00000 0.00000 0.00000 0.00005

RIER --- 0301 1 -----------.-D-------.--------------W-------------- 0 0 0 1 0.00000 0.00000 0.00000 0.00005

RIER --- 0302 1 -----------.-DK---I--.----------------------------- 0 0 0 1 0.00000 0.00000 0.00000 0.00005

RIER --- 0303 1 -----------.---------.--------------F-------------- 3 0 0 1 0.00013 0.00000 0.00000 0.00005

RIER --- 0304 1 -----------.---------.--------------S-------------- 0 0 0 1 0.00000 0.00000 0.00000 0.00005

RIER --- 0305 1 ----------T.-G-------.----------------------------- 0 0 0 1 0.00000 0.00000 0.00000 0.00005

RIER --- 0306 1 -----------.---------.-----R-D--------------------- 0 0 0 1 0.00000 0.00000 0.00000 0.00005

RIER --- 0307 1 -----------.-D-------.---------------V------------- 0 0 0 1 0.00000 0.00000 0.00000 0.00005

RIER --- 0308 1 ----------D.-D-------.----------------------------- 0 0 0 1 0.00000 0.00000 0.00000 0.00005

RIER --- 0309 1 -----------.V--------.-------D--------------------- 0 0 0 1 0.00000 0.00000 0.00000 0.00005

RIER --- 0310 1 -----------.-DK------.-------------------R--------- 0 0 0 1 0.00000 0.00000 0.00000 0.00005

RIER --- 0311 1 -----------.-D-------.--E-------------------------- 0 0 0 1 0.00000 0.00000 0.00000 0.00005

RIER --- 0312 1 -----------.----C----.----------------------------- 0 0 0 1 0.00000 0.00000 0.00000 0.00005

RIER --- 0313 1 ----M---S--.---------.-------D--------------------- 0 0 0 1 0.00000 0.00000 0.00000 0.00005

RIER --- 0314 1 -----------.-D-------.----------------------N------ 0 0 0 1 0.00000 0.00000 0.00000 0.00005

RIER --- 0315 1 ----------D.---------.-------D--------------------- 0 0 0 1 0.00000 0.00000 0.00000 0.00005

RIER --- 0316 1 -----------.---------.----A------------------------ 1 0 0 1 0.00004 0.00000 0.00000 0.00005

RIER --- 0317 1 -----------.---------.------------------------K---- 2 0 0 1 0.00009 0.00000 0.00000 0.00005

RIER --- 0318 1 -----------.---------.------T---------------------- 2 0 0 1 0.00009 0.00000 0.00000 0.00005

RIER --- 0319 1 -----------.---------.---S---D-I------------------- 0 0 0 1 0.00000 0.00000 0.00000 0.00005

RIER --- 0320 1 -------V---.---------.----------------------------- 0 0 1 1 0.00000 0.00000 0.00017 0.00005

RIER --- 0321 1 -----------.---------.----------------T------------ 0 0 0 1 0.00000 0.00000 0.00000 0.00005

RIER --- 0322 1 I----------.-D-------.-------D--------------------- 0 0 0 1 0.00000 0.00000 0.00000 0.00005

RIER --- 0323 1 -----------.---------.----------------------------A 1 0 0 1 0.00004 0.00000 0.00000 0.00005

RIER --- 0324 1 -----------.---------.-------D-----------R--------- 0 0 0 1 0.00000 0.00000 0.00000 0.00005

RIER --- 0325 1 -----------.---------.-E--------------------------- 14 8 0 1 0.00060 0.00068 0.00000 0.00005

RIER --- 0326 1 ----M------.-DK------.-V--------------------------- 0 0 0 1 0.00000 0.00000 0.00000 0.00005

RIER --- 0327 1 ---------N-.-G-------.----------------------------- 0 0 0 1 0.00000 0.00000 0.00000 0.00005

RIER --- 0328 1 --------Y--.-D-------.--------------------T-------- 0 0 0 1 0.00000 0.00000 0.00000 0.00005

RIER --- 0329 1 -----------.---------.--L-------------------------- 1 1 0 1 0.00004 0.00008 0.00000 0.00005

RIER --- 0331 1 ---------I-.---------.----------------------------- 2 0 0 1 0.00009 0.00000 0.00000 0.00005

RIER --- 0332 1 -----------.--------H.-------D--------------------- 0 0 0 1 0.00000 0.00000 0.00000 0.00005

RIER --- 0333 1 -----------.-D-------.------------P---------------- 0 0 0 1 0.00000 0.00000 0.00000 0.00005

RIER --- 0334 1 ----M----N-.---------.----------------------------- 0 0 0 1 0.00000 0.00000 0.00000 0.00005

RIER --- 0335 1 -----------.---------.-----S----------------------- 0 0 0 1 0.00000 0.00000 0.00000 0.00005

RIER --- 0336 1 ----M------.-----A---.----------------------------- 0 0 0 1 0.00000 0.00000 0.00000 0.00005

RIER --- 0337 1 -----------.-DK------.-------D------Y--N----------- 0 0 0 1 0.00000 0.00000 0.00000 0.00005

RIER --- 0338 1 I----------.---------.-------D--------------------- 0 0 0 1 0.00000 0.00000 0.00000 0.00005

RIER --- 0339 1 -----------.-D-------.----------------------------A 0 0 0 1 0.00000 0.00000 0.00000 0.00005

RIER --- 0340 1 ----L------.-D-------.----------------------------- 0 0 0 1 0.00000 0.00000 0.00000 0.00005

RIER --- 0341 1 -----------.-D-------.-------------I--------------- 0 0 0 1 0.00000 0.00000 0.00000 0.00005

RIER --- 0342 1 ----M-----T.---------.-------D--------------------- 0 0 0 1 0.00000 0.00000 0.00000 0.00005

RIER --- 0343 1 --I--------.-D-------.----------------------------- 0 0 0 1 0.00000 0.00000 0.00000 0.00005

RIER --- 0345 1 L----------.---------.----------------------------- 0 1 0 1 0.00000 0.00008 0.00000 0.00005

RIER --- 0346 1 ----T------.--K------.----------------------------- 0 0 1 1 0.00000 0.00000 0.00017 0.00005

RIER --- 0347 1 -----------.-DK--I---.--------T-------------------- 0 0 0 1 0.00000 0.00000 0.00000 0.00005

RIER --- 0349 1 ----M------.---------.-------D-A------------------- 0 0 0 1 0.00000 0.00000 0.00000 0.00005

RIER --- 0350 1 -----------.SD-------.----------------------------- 0 0 0 1 0.00000 0.00000 0.00000 0.00005

RIER --- 0351 1 -----------.---------.-----R----------------------- 1 2 0 1 0.00004 0.00017 0.00000 0.00005

RIER --- 0352 1 -----------.-D-------.-------------L--------------- 0 0 0 1 0.00000 0.00000 0.00000 0.00005

RIER --- 0353 1 -----------.-DK-R----.----------------------------- 0 0 0 1 0.00000 0.00000 0.00000 0.00005

RIER --- 0354 1 ----------D.---------.---------------------------S- 0 0 0 1 0.00000 0.00000 0.00000 0.00005

RIER --- 0355 1 ---V-------.---------.----------------------------- 1 1 0 1 0.00004 0.00008 0.00000 0.00005

RIER --- 0356 1 ----------V.---------.--------------------T-------- 0 0 0 1 0.00000 0.00000 0.00000 0.00005

RIER --- 0357 1 -----------.-D-----F-.----------------------------- 0 0 0 1 0.00000 0.00000 0.00000 0.00005

RIER --- 0358 1 -----------.-DK------.--------------WL------------- 0 0 0 1 0.00000 0.00000 0.00000 0.00005

RIER --- 0359 1 ----M------.------I--.----------------------------- 0 0 0 1 0.00000 0.00000 0.00000 0.00005

RIER --- 0360 1 --P--------.-D-------.----------------------------- 0 0 0 1 0.00000 0.00000 0.00000 0.00005

RIER --- 0361 1 ---W-------.---------.----------------------------- 3 0 0 1 0.00013 0.00000 0.00000 0.00005

RIER --- 0363 1 -----------.---------.---L---D--------------------- 0 0 0 1 0.00000 0.00000 0.00000 0.00005

RIER --- 0364 1 -----------.---------.---------------G------------- 1 0 0 1 0.00004 0.00000 0.00000 0.00005

RIER --- 0365 1 -----------.-D-------.---------------------H------- 0 0 0 1 0.00000 0.00000 0.00000 0.00005

RIER --- 0366 1 --------R--.-DK------.----------------------------- 0 0 0 1 0.00000 0.00000 0.00000 0.00005

RIER --- 0367 1 ----M------.---------.---H------------------------- 0 0 0 1 0.00000 0.00000 0.00000 0.00005

RIER --- 0368 1 -----------.-DK------.------------------S---------- 0 0 0 1 0.00000 0.00000 0.00000 0.00005

RIER --- 0369 1 ----------T.-DK------.-------D--------------------- 0 0 0 1 0.00000 0.00000 0.00000 0.00005

RIER --- 0370 1 ----R------.-D-------.----------------------------- 0 0 0 1 0.00000 0.00000 0.00000 0.00005

RIER --- 0371 1 -----------.--K------.-------DV-------------------- 0 0 0 1 0.00000 0.00000 0.00000 0.00005

RIER --- 0373 1 -----------.-D-------.--------------------V-------- 0 0 0 1 0.00000 0.00000 0.00000 0.00005

RIER --- 0374 1 ----M------.---------.---------------------------Y- 0 0 0 1 0.00000 0.00000 0.00000 0.00005

RIER --- 0375 1 --R-M------.---------.----------------------------- 0 0 0 1 0.00000 0.00000 0.00000 0.00005

RIER --- 0376 1 ----M------.-G-------.-------D--------------------- 0 0 0 1 0.00000 0.00000 0.00000 0.00005

RIER --- 0378 1 -----------.-G-------.--------T-------------------- 0 0 0 1 0.00000 0.00000 0.00000 0.00005

RIER --- 0379 1 ----M------.-DK------.--------T-------------------- 0 0 0 1 0.00000 0.00000 0.00000 0.00005

RIER --- 0380 1 ----------V.---------.-------D--------------------- 0 0 0 1 0.00000 0.00000 0.00000 0.00005

RIER --- 0381 1 -----------.-G-------.---------I------------------- 0 0 0 1 0.00000 0.00000 0.00000 0.00005

RIER --- 0382 1 -----------.---------.-------D------------E-------- 0 0 0 1 0.00000 0.00000 0.00000 0.00005

RIER --- 0383 1 ----T------.-DK------.----------------------------- 0 0 0 1 0.00000 0.00000 0.00000 0.00005

RIER --- 0384 1 -----------.---------.-----------------G----------- 0 2 0 1 0.00000 0.00017 0.00000 0.00005

RIER --- 0385 1 ----M------.---------.--------------------T-------- 0 0 0 1 0.00000 0.00000 0.00000 0.00005

RIER --- 0386 1 -----------.-D-------.-----------I----------------- 0 0 0 1 0.00000 0.00000 0.00000 0.00005

RIER --- 0387 1 -----------.------A--.----------------------------- 2 7 1 1 0.00009 0.00059 0.00017 0.00005

RIER --- 0388 1 -I---------.-G-------.----------------------------- 0 0 0 1 0.00000 0.00000 0.00000 0.00005

RIER --- 0389 1 ------I----.-D-------.----------------------------- 0 0 0 1 0.00000 0.00000 0.00000 0.00005

RIER --- 0390 1 ----M------.-G-------.--------------------T-------- 0 0 0 1 0.00000 0.00000 0.00000 0.00005

RIER --- 0391 1 --------G--.-DK------.----------------------------- 0 0 0 1 0.00000 0.00000 0.00000 0.00005

RIER --- 0392 1 -----------.---------.-------D-----Y--------------- 0 0 0 1 0.00000 0.00000 0.00000 0.00005

RIER --- 0393 1 -----------.-G-------.--------V-------------------- 0 0 0 1 0.00000 0.00000 0.00000 0.00005

RIER --- 0394 1 -----------.-D-------.-------D-------V------------- 0 0 0 1 0.00000 0.00000 0.00000 0.00005

RIER --- 0395 1 --I--------.-DK------.----------------------------- 0 0 0 1 0.00000 0.00000 0.00000 0.00005

RIER --- 0396 1 ----M------.---------.---------------------------S- 0 0 0 1 0.00000 0.00000 0.00000 0.00005

RIER --- 0397 1 ----M------.--K------.-------D--------------------- 0 0 0 1 0.00000 0.00000 0.00000 0.00005

RIER --- 0398 1 -----------.-G-------.---------------------F------- 0 0 0 1 0.00000 0.00000 0.00000 0.00005

RIER --- 0399 1 ----------T.---------.--------------------V-------- 0 0 0 1 0.00000 0.00000 0.00000 0.00005

RIER --- 0400 1 --F--------.-D-------.----------------------------- 0 0 0 1 0.00000 0.00000 0.00000 0.00005

RIER --- 0401 1 ----M------.---------.-------D------------------Y-- 0 0 0 1 0.00000 0.00000 0.00000 0.00005

RIER --- 0402 1 I----------.---------.----------------------------- 4 3 0 1 0.00017 0.00025 0.00000 0.00005

RIER --- 0403 1 -----------.-DK------.----------------------------I 0 0 0 1 0.00000 0.00000 0.00000 0.00005

RIER --- 0404 1 -----------.-D-------.-----------------------AS--S- 0 0 0 1 0.00000 0.00000 0.00000 0.00005

**_______________________________________________________________________________________________________________________**

**CH40**

**ID Day Rank Tally _____________Sequence__________________ ________Tally______ _______Frequency_______**

Transmitted - - - SQHGMDDPEREVLVWRFDS.SLAFRHVAR.ELHPEYYKNC d00 d16 d45 d00 d16 d45

Bcon - - - -L-------------K---.R---H-M--.--------D-

Transmitted form:

CH40.Nef d00 0001 3917 -------------------.---------.---------- 3916 3617 7 0.96739 0.46840 0.00374

CH40.Nef d16 0001 3617 -------------------.---------.---------- 3916 3617 7 0.96739 0.46840 0.00374

CH40.Nef d45 0004 7 -------------------.---------.---------- 3916 3617 7 0.96739 0.46840 0.00374

Major escape day 45 (R/Q), anchor motif lost:

CH40.Nef d00 0040 1 -------------------.--------Q.---------- 1 304 1560 0.00025 0.03937 0.83289

CH40.Nef d16 0005 304 -------------------.--------Q.---------- 1 304 1560 0.00025 0.03937 0.83289

CH40.Nef d16 0041 4 -------------------.-------VQ.---------- 0 4 3 0.00000 0.00052 0.00160

CH40.Nef d16 0057 2 -------------------.--------Q.G--------- 0 2 0 0.00000 0.00026 0.00000

CH40.Nef d16 0089 2 ----------------S--.--------Q.---------- 0 2 2 0.00000 0.00026 0.00107

CH40.Nef d16 0097 1 ------------S------.--------Q.---------- 0 1 0 0.00000 0.00013 0.00000

CH40.Nef d16 0101 1 -------------M-----.--------Q.---------- 0 1 0 0.00000 0.00013 0.00000

CH40.Nef d16 0102 1 -------------------.--------Q.-P-------- 0 1 2 0.00000 0.00013 0.00107

CH40.Nef d16 0107 1 -------------------.---L----Q.---------- 0 1 2 0.00000 0.00013 0.00107

CH40.Nef d16 0109 1 -------------------.--E-----Q.---------- 0 1 0 0.00000 0.00013 0.00000

CH40.Nef d16 0121 1 -------------------.--T-----Q.---------- 0 1 0 0.00000 0.00013 0.00000

CH40.Nef d16 0134 1 G------------------.--------Q.---------- 0 1 2 0.00000 0.00013 0.00107

CH40.Nef d16 0149 1 ----V--------------.--------Q.---------- 0 1 0 0.00000 0.00013 0.00000

CH40.Nef d16 0162 1 -----------------G-.--------Q.---------- 0 1 1 0.00000 0.00013 0.00053

CH40.Nef d16 0166 1 ----------G--------.--------Q.G--------- 0 1 0 0.00000 0.00013 0.00000

CH40.Nef d16 0176 1 -----G-------------.--------Q.---------- 0 1 5 0.00000 0.00013 0.00267

CH40.Nef d16 0178 1 -------------G-----.--------Q.---------- 0 1 0 0.00000 0.00013 0.00000

CH40.Nef d45 0001 1560 -------------------.--------Q.---------- 1 304 1560 0.00025 0.03937 0.83289

CH40.Nef d45 0005 5 ---------------K---.--------Q.---------- 0 0 5 0.00000 0.00000 0.00267

CH40.Nef d45 0006 5 -----G-------------.--------Q.---------- 0 1 5 0.00000 0.00013 0.00267

CH40.Nef d45 0007 5 ---------K---------.--------Q.---------- 0 0 5 0.00000 0.00000 0.00267

CH40.Nef d45 0008 5 -------------------.------A-Q.---------- 0 0 5 0.00000 0.00000 0.00267

CH40.Nef d45 0009 4 ------------------G.--------Q.---------- 0 0 4 0.00000 0.00000 0.00214

CH40.Nef d45 0010 3 --------------R----.--------Q.---------- 0 0 3 0.00000 0.00000 0.00160

CH40.Nef d45 0011 3 -------------------.-------VQ.---------- 0 4 3 0.00000 0.00052 0.00160

CH40.Nef d45 0012 3 ------------------N.--------Q.---------- 0 0 3 0.00000 0.00000 0.00160

CH40.Nef d45 0013 2 -------S-----------.--------Q.---------- 0 0 2 0.00000 0.00000 0.00107

CH40.Nef d45 0014 2 -------------------.--------Q.-P-------- 0 1 2 0.00000 0.00013 0.00107

CH40.Nef d45 0015 2 -------------A-----.--------Q.---------- 0 0 2 0.00000 0.00000 0.00107

CH40.Nef d45 0016 2 --Q----------------.--------Q.---------- 0 0 2 0.00000 0.00000 0.00107

CH40.Nef d45 0017 2 -R-----------------.--------Q.---------- 0 0 2 0.00000 0.00000 0.00107

CH40.Nef d45 0018 2 -------------------.---L----Q.---------- 0 1 2 0.00000 0.00013 0.00107

CH40.Nef d45 0019 2 -------------------.--------Q.--------D- 0 0 2 0.00000 0.00000 0.00107

CH40.Nef d45 0020 2 -------------------.-------TQ.---------- 0 0 2 0.00000 0.00000 0.00107

CH40.Nef d45 0021 2 G------------------.--------Q.---------- 0 1 2 0.00000 0.00013 0.00107

CH40.Nef d45 0022 2 ----------------S--.--------Q.---------- 0 2 2 0.00000 0.00026 0.00107

CH40.Nef d45 0025 1 C------------------.--------Q.---------- 0 0 1 0.00000 0.00000 0.00053

CH40.Nef d45 0026 1 ------------------C.--------Q.---------- 0 0 1 0.00000 0.00000 0.00053

CH40.Nef d45 0027 1 ---R---------------.--------Q.---------- 0 0 1 0.00000 0.00000 0.00053

CH40.Nef d45 0031 1 -------------------.--------Q.----G----- 0 0 1 0.00000 0.00000 0.00053

CH40.Nef d45 0032 1 -------------------.------G-Q.---------- 0 0 1 0.00000 0.00000 0.00053

CH40.Nef d45 0033 1 -------------------.--------Q.--Y------- 0 0 1 0.00000 0.00000 0.00053

CH40.Nef d45 0034 1 -------------------.G-------Q.---------- 0 0 1 0.00000 0.00000 0.00053

CH40.Nef d45 0035 1 -----------------G-.--------Q.---------- 0 1 1 0.00000 0.00013 0.00053

CH40.Nef d45 0036 1 ---E---------------.--------Q.---------- 0 0 1 0.00000 0.00000 0.00053

CH40.Nef d45 0038 1 ---------I--------G.--------Q.---------- 0 0 1 0.00000 0.00000 0.00053

CH40.Nef d45 0041 1 N------------------.--------Q.---------- 0 0 1 0.00000 0.00000 0.00053

CH40.Nef d45 0042 1 -------------------.---S----Q.---------- 0 0 1 0.00000 0.00000 0.00053

CH40.Nef d45 0043 1 ----------------L--.--------Q.---------- 0 0 1 0.00000 0.00000 0.00053

CH40.Nef d45 0044 1 ------G------------.--------Q.---------- 0 0 1 0.00000 0.00000 0.00053

CH40.Nef d45 0047 1 --------G----------.--------Q.---------- 0 0 1 0.00000 0.00000 0.00053

CH40.Nef d45 0048 1 -------------------.--------Q.------C--- 0 0 1 0.00000 0.00000 0.00053

CH40.Nef d45 0050 1 -------------------.--------Q.--------S- 0 0 1 0.00000 0.00000 0.00053

CH40.Nef d45 0051 1 ---------------G---.--------Q.---------- 0 0 1 0.00000 0.00000 0.00053

CH40.Nef d45 0054 1 -----------A-------.--------Q.---------- 0 0 1 0.00000 0.00000 0.00053

CH40.Nef d45 0055 1 -------------------.-----R--Q.---------- 0 0 1 0.00000 0.00000 0.00053

CH40.Nef d45 0059 1 --Y----------------.--------Q.---------- 0 0 1 0.00000 0.00000 0.00053

CH40.Nef d45 0060 1 -------------------.-P------Q.---------- 0 0 1 0.00000 0.00000 0.00053

CH40.Nef d45 0061 1 ----------------I--.--------Q.---------- 0 0 1 0.00000 0.00000 0.00053

CH40.Nef d45 0062 1 -------------------.--------Q.-M-------- 0 0 1 0.00000 0.00000 0.00053

CH40.Nef d45 0063 1 -------------------.--------Q.-----C---- 0 0 1 0.00000 0.00000 0.00053

CH40.Nef d45 0065 1 ----T--------------.--------Q.---------- 0 0 1 0.00000 0.00000 0.00053

CH40.Nef d45 0066 1 -------------------.--------Q.----K----- 0 0 1 0.00000 0.00000 0.00053

CH40.Nef d45 0067 1 -L-----------------.--------Q.---------- 0 0 1 0.00000 0.00000 0.00053

Day 16 most common escape form (R/H), diminishing by d45, B consensus form:

CH40.Nef d16 0126 1 -------------------.----H---Q.---------- 0 1 0 0.00000 0.00013 0.00000

CH40.Nef d16 0002 1797 -------------------.----H----.---------- 0 1797 160 0.00000 0.23271 0.08542

CH40.Nef d16 0027 5 -----G-------------.----H----.---------- 0 5 0 0.00000 0.00065 0.00000

CH40.Nef d16 0033 4 ----------G--------.----H----.---------- 0 4 0 0.00000 0.00052 0.00000

CH40.Nef d16 0034 4 -------------------.----H----.------C--- 0 4 2 0.00000 0.00052 0.00107

CH40.Nef d16 0038 4 -------------------.----H----.G--------- 0 4 0 0.00000 0.00052 0.00000

CH40.Nef d16 0040 4 -------------------.----HR---.---------- 0 4 0 0.00000 0.00052 0.00000

CH40.Nef d16 0042 3 --------------R----.----H----.---------- 0 3 1 0.00000 0.00039 0.00053

CH40.Nef d16 0044 3 ----V--------------.----H----.---------- 0 3 0 0.00000 0.00039 0.00000

CH40.Nef d16 0045 3 ----------------L--.----H----.---------- 0 3 0 0.00000 0.00039 0.00000

CH40.Nef d16 0048 3 -------------------.----H----.-P-------- 0 3 1 0.00000 0.00039 0.00053

CH40.Nef d16 0064 2 -------------------.----H----.---------R 0 2 0 0.00000 0.00026 0.00000

CH40.Nef d16 0069 2 -------------A-----.----H----.---------- 0 2 0 0.00000 0.00026 0.00000

CH40.Nef d16 0073 2 -------------------.-P--H----.---------- 0 2 0 0.00000 0.00026 0.00000

CH40.Nef d16 0074 2 -------------------.----H--V-.---------- 0 2 1 0.00000 0.00026 0.00053

CH40.Nef d16 0081 2 G------------------.----H----.---------- 0 2 1 0.00000 0.00026 0.00053

CH40.Nef d16 0082 2 -R-----------------.----H----.---------- 0 2 0 0.00000 0.00026 0.00000

CH40.Nef d16 0086 2 -----------------G-.----H----.---------- 0 2 1 0.00000 0.00026 0.00053

CH40.Nef d16 0087 2 ------------------G.----H----.---------- 0 2 0 0.00000 0.00026 0.00000

CH40.Nef d16 0088 2 N------------------.----H----.---------- 0 2 1 0.00000 0.00026 0.00053

CH40.Nef d16 0091 2 -------------------.----H----.------H--- 0 2 0 0.00000 0.00026 0.00000

CH40.Nef d16 0098 1 ----------D--------.----H----.---------- 0 1 0 0.00000 0.00013 0.00000

CH40.Nef d16 0108 1 ----------------S--.----H----.---------- 0 1 0 0.00000 0.00013 0.00000

CH40.Nef d16 0112 1 -------------------.----H----.-Q-------- 0 1 0 0.00000 0.00013 0.00000

CH40.Nef d16 0115 1 ----------------L--.----H--V-.---------- 0 1 0 0.00000 0.00013 0.00000

CH40.Nef d16 0120 1 ------------S------.----H----.---------- 0 1 0 0.00000 0.00013 0.00000

CH40.Nef d16 0122 1 -------------------.----H----.--C------- 0 1 0 0.00000 0.00013 0.00000

CH40.Nef d16 0124 1 ------G------------.----H----.---------- 0 1 0 0.00000 0.00013 0.00000

CH40.Nef d16 0127 1 -----N-------------.----H----.---------- 0 1 0 0.00000 0.00013 0.00000

CH40.Nef d16 0128 1 -------------------.G---H----.---------- 0 1 0 0.00000 0.00013 0.00000

CH40.Nef d16 0130 1 -------------------.----H--T-.---------- 0 1 0 0.00000 0.00013 0.00000

CH40.Nef d16 0133 1 -----E-------------.----H----.---------- 0 1 0 0.00000 0.00013 0.00000

CH40.Nef d16 0135 1 -------------------.----H-A--.---------- 0 1 1 0.00000 0.00013 0.00053

CH40.Nef d16 0145 1 -------------------.----H----.-------R-- 0 1 0 0.00000 0.00013 0.00000

CH40.Nef d16 0152 1 -------------------.----H----.--Y------- 0 1 0 0.00000 0.00013 0.00000

CH40.Nef d16 0155 1 ---------------K---.----H----.---------- 0 1 0 0.00000 0.00013 0.00000

CH40.Nef d16 0157 1 ----T--------------.----H----.---------- 0 1 0 0.00000 0.00013 0.00000

CH40.Nef d16 0159 1 ----------------V--.----H----.---------- 0 1 0 0.00000 0.00013 0.00000

CH40.Nef d16 0160 1 ----R--------------.----H----.---------- 0 1 0 0.00000 0.00013 0.00000

CH40.Nef d16 0165 1 -------------------.--V-H----.---------- 0 1 0 0.00000 0.00013 0.00000

CH40.Nef d16 0171 1 -------------------.----H----.----G----- 0 1 0 0.00000 0.00013 0.00000

CH40.Nef d16 0175 1 -------------------.----H----.-P-----N-- 0 1 0 0.00000 0.00013 0.00000

CH40.Nef d16 0179 1 -------------------.----H----.--R------- 0 1 0 0.00000 0.00013 0.00000

CH40.Nef d16 0188 1 --R----------------.----H----.---------- 0 1 0 0.00000 0.00013 0.00000

CH40.Nef d16 0193 1 G----G-------------.----H----.---------- 0 1 0 0.00000 0.00013 0.00000

CH40.Nef d16 0194 1 ------G------------.----H--V-.---------- 0 1 0 0.00000 0.00013 0.00000

CH40.Nef d16 0195 1 ----------------Y--.----H----.---------- 0 1 0 0.00000 0.00013 0.00000

CH40.Nef d16 0197 1 -------------------.---IH----.---------- 0 1 0 0.00000 0.00013 0.00000

CH40.Nef d45 0002 160 -------------------.----H----.---------- 0 1797 160 0.00000 0.23271 0.08542

CH40.Nef d45 0024 2 -------------------.----H----.------C--- 0 4 2 0.00000 0.00052 0.00107

CH40.Nef d45 0028 1 -------------------.----H----.-P-------- 0 3 1 0.00000 0.00039 0.00053

CH40.Nef d45 0029 1 --------------R----.----H----.---------- 0 3 1 0.00000 0.00039 0.00053

CH40.Nef d45 0039 1 -------------------.----H--V-.---------- 0 2 1 0.00000 0.00026 0.00053

CH40.Nef d45 0040 1 -------------------.----H----.---L------ 0 0 1 0.00000 0.00000 0.00053

CH40.Nef d45 0045 1 G------------------.----H----.---------- 0 2 1 0.00000 0.00026 0.00053

CH40.Nef d45 0046 1 -----------------E-.----H----.---------- 0 0 1 0.00000 0.00000 0.00053

CH40.Nef d45 0052 1 -----------------G-.----H----.---------- 0 2 1 0.00000 0.00026 0.00053

CH40.Nef d45 0053 1 N------------------.----H----.---------- 0 2 1 0.00000 0.00026 0.00053

CH40.Nef d45 0057 1 -------------------.----H-A--.---------- 0 1 1 0.00000 0.00013 0.00053

CH40.Nef d45 0058 1 -------------------.---SH----.---------- 0 0 1 0.00000 0.00000 0.00053

CH40.Nef d45 0064 1 -----V-------------.----H----.---------- 0 0 1 0.00000 0.00000 0.00053

d16 (V/M) form lost by d45, B consensus form:

CH40.Nef con 0020 1 -------------------.------M--.---------- 0 403 1 0.00000 0.05219 0.00053

CH40.Nef d16 0004 403 -------------------.------M--.---------- 0 403 1 0.00000 0.05219 0.00053

CH40.Nef d45 0056 1 -------------------.------M--.---------- 0 403 1 0.00000 0.05219 0.00053

CH40.Nef d16 0022 7 -----N-------------.------M--.---------- 0 7 0 0.00000 0.00091 0.00000

CH40.Nef d16 0056 2 -----------------G-.------M--.---------- 0 2 0 0.00000 0.00026 0.00000

CH40.Nef d16 0104 1 ----------------S--.------M--.---------- 0 1 0 0.00000 0.00013 0.00000

CH40.Nef d16 0105 1 -------------------.---S--M--.---------- 0 1 0 0.00000 0.00013 0.00000

CH40.Nef d16 0143 1 --------G----------.------M--.---------- 0 1 0 0.00000 0.00013 0.00000

CH40.Nef d16 0148 1 -------------M-----.------M--.---------- 0 1 0 0.00000 0.00013 0.00000

CH40.Nef d16 0151 1 ----V--------------.------M--.---------- 0 1 0 0.00000 0.00013 0.00000

CH40.Nef d16 0163 1 ------------------G.------M--.---------- 0 1 0 0.00000 0.00013 0.00000

CH40.Nef d16 0170 1 ----T--------------.------M--.---------- 0 1 0 0.00000 0.00013 0.00000

CH40.Nef d16 0173 1 -------------------.---L--M--.---------- 0 1 0 0.00000 0.00013 0.00000

CH40.Nef d16 0185 1 ------G------------.------M--.---------- 0 1 0 0.00000 0.00013 0.00000

CH40.Nef d16 0191 1 -------------------.------M--.----G----- 0 1 0 0.00000 0.00013 0.00000

Steady low level form (S/N):

CH40.Nef d16 0093 1 -------------------.N---H----.--Y------- 0 1 0 0.00000 0.00013 0.00000

CH40.Nef d00 0032 1 -------------------.N--------.---------- 1 133 46 0.00025 0.01722 0.02456

CH40.Nef d16 0008 133 -------------------.N--------.---------- 1 133 46 0.00025 0.01722 0.02456

CH40.Nef d45 0003 46 -------------------.N--------.---------- 1 133 46 0.00025 0.01722 0.02456

CH40.Nef d45 0030 1 -------------------.N-------Q.---------- 0 0 1 0.00000 0.00000 0.00053

CH40.Nef d16 0043 3 ----------K--------.N--------.---------- 0 3 0 0.00000 0.00039 0.00000

CH40.Nef d16 0103 1 -----G-------------.N--------.---------- 0 1 0 0.00000 0.00013 0.00000

CH40.Nef d16 0117 1 -------------------.N--------.-------R-- 0 1 0 0.00000 0.00013 0.00000

CH40.Nef d16 0186 1 -------------------.N-----A--.---------- 0 1 0 0.00000 0.00013 0.00000

CH40.Nef d16 0189 1 -------------A-----.N--------.---------- 0 1 0 0.00000 0.00013 0.00000

CH40.Nef d45 0049 1 N------------------.N--------.---------- 0 0 1 0.00000 0.00000 0.00053

d16 form (S/R) lost by d45, B consensus form:

CH40.Nef d16 0014 19 -------------------.R---H----.---------- 0 19 0 0.00000 0.00246 0.00000

CH40.Nef d16 0094 1 -P-----------------.R---H----.---------- 0 1 0 0.00000 0.00013 0.00000

CH40.Nef d16 0006 268 -------------------.R--------.---------- 0 268 2 0.00000 0.03471 0.00107

CH40.Nef d45 0023 2 -------------------.R--------.---------- 0 268 2 0.00000 0.03471 0.00107

CH40.Nef d16 0180 1 -------------------.R-----M--.---------- 0 1 0 0.00000 0.00013 0.00000

CH40.Nef d16 0051 3 --------G----------.R--------.---------- 0 3 0 0.00000 0.00039 0.00000

CH40.Nef d16 0079 2 ----V--------------.R--------.---------- 0 2 0 0.00000 0.00026 0.00000

CH40.Nef d16 0100 1 -------------------.R--------.--------D- 0 1 0 0.00000 0.00013 0.00000

CH40.Nef d16 0114 1 -----------------G-.R--------.---------- 0 1 0 0.00000 0.00013 0.00000

CH40.Nef d16 0118 1 ---------------G---.R--------.---------- 0 1 0 0.00000 0.00013 0.00000

CH40.Nef d16 0136 1 --------------R----.R--------.---------- 0 1 0 0.00000 0.00013 0.00000

CH40.Nef d16 0140 1 -------------------.R---C----.---------- 0 1 0 0.00000 0.00013 0.00000

CH40.Nef d16 0141 1 ------G------------.R--------.-------E-- 0 1 0 0.00000 0.00013 0.00000

CH40.Nef d16 0150 1 -------S-----------.R--------.---------- 0 1 0 0.00000 0.00013 0.00000

CH40.Nef d16 0154 1 -------------------.R-----A--.---------- 0 1 0 0.00000 0.00013 0.00000

CH40.Nef d16 0169 1 -------------------.R--------.-----H---- 0 1 0 0.00000 0.00013 0.00000

CH40.Nef d16 0172 1 -----------------N-.R--------.---------- 0 1 0 0.00000 0.00013 0.00000

CH40.Nef d16 0182 1 -------------------.R------D-.---------- 0 1 0 0.00000 0.00013 0.00000

CH40.Nef d16 0202 1 ------G------------.R--------.---------- 0 1 0 0.00000 0.00013 0.00000

d16 form (V/E) but lost by d45:

CH40.Nef d16 0007 141 -------------------.------E--.---------- 0 141 1 0.00000 0.01826 0.00053

CH40.Nef d45 0037 1 -------------------.------E--.---------- 0 141 1 0.00000 0.01826 0.00053

CH40.Nef d16 0060 2 -------------------.------E--.-P-------- 0 2 0 0.00000 0.00026 0.00000

Other day 0 sequences:

CH40.Nef d00 0002 12 -------------------.---------.-P-------- 12 5 0 0.00296 0.00065 0.00000

CH40.Nef d00 0003 9 ------G------------.---------.---------- 9 0 0 0.00222 0.00000 0.00000

CH40.Nef d00 0004 8 -----G-------------.---------.---------- 8 9 0 0.00198 0.00117 0.00000

CH40.Nef d00 0005 8 ----V--------------.---------.---------- 8 2 0 0.00198 0.00026 0.00000

CH40.Nef d00 0006 5 -----------------V-.---------.---------- 5 0 0 0.00124 0.00000 0.00000

CH40.Nef d00 0007 5 -------------------.------A--.---------- 5 36 0 0.00124 0.00466 0.00000

CH40.Nef d00 0008 5 ----T--------------.---------.---------- 5 2 0 0.00124 0.00026 0.00000

CH40.Nef d00 0009 5 -------------------.-P-------.---------- 5 8 0 0.00124 0.00104 0.00000

CH40.Nef d00 0010 4 -------------------.-------V-.---------- 4 6 0 0.00099 0.00078 0.00000

CH40.Nef d00 0011 4 -------------------.---S-----.---------- 4 2 0 0.00099 0.00026 0.00000

CH40.Nef d00 0012 4 -------------------.---------.------C--- 4 0 0 0.00099 0.00000 0.00000

CH40.Nef d00 0013 4 G------------------.---------.---------- 4 5 0 0.00099 0.00065 0.00000

CH40.Nef d00 0014 4 ------------------G.---------.---------- 4 12 0 0.00099 0.00155 0.00000

CH40.Nef d00 0015 3 -------------------.---------.---------Y 3 0 0 0.00074 0.00000 0.00000

CH40.Nef d00 0016 3 -R-----------------.---------.---------- 3 2 0 0.00074 0.00026 0.00000

CH40.Nef d00 0017 2 -------------------.G--------.---------- 2 51 0 0.00049 0.00660 0.00000

CH40.Nef d00 0018 2 -------------------.-----R---.---------- 2 2 0 0.00049 0.00026 0.00000

CH40.Nef d00 0019 2 ---------G---------.---------.---------- 2 0 0 0.00049 0.00000 0.00000

CH40.Nef d00 0020 2 --------------R----.---------.---------- 2 4 0 0.00049 0.00052 0.00000

CH40.Nef d00 0021 2 -------S-----------.---------.---------- 2 0 0 0.00049 0.00000 0.00000

CH40.Nef d00 0022 2 -------------------.---L-----.---------- 2 4 0 0.00049 0.00052 0.00000

CH40.Nef d00 0023 2 -----------------G-.---------.---------- 2 2 0 0.00049 0.00026 0.00000

CH40.Nef d00 0024 2 -----------A-------.---------.---------- 2 0 0 0.00049 0.00000 0.00000

CH40.Nef d00 0025 2 -------------------.---------.G--------- 2 3 0 0.00049 0.00039 0.00000

CH40.Nef d00 0026 2 -------------------.---------.-----C---- 2 4 0 0.00049 0.00052 0.00000

CH40.Nef d00 0027 2 ----------------L--.---------.---------- 2 6 0 0.00049 0.00078 0.00000

CH40.Nef d00 0028 1 -------------------.---------.-Q-------- 1 2 0 0.00025 0.00026 0.00000

CH40.Nef d00 0029 1 -------------------.------G--.---------- 1 1 0 0.00025 0.00013 0.00000

CH40.Nef d00 0030 1 -------------M-----.---------.---------- 1 0 0 0.00025 0.00000 0.00000

CH40.Nef d00 0031 1 --------G----------.---------.---------- 1 1 0 0.00025 0.00013 0.00000

CH40.Nef d00 0033 1 -------------------.---------.K--------- 1 0 0 0.00025 0.00000 0.00000

CH40.Nef d00 0034 1 -------------------.---Y-----.---------- 1 0 0 0.00025 0.00000 0.00000

CH40.Nef d00 0035 1 -------------------.-Q-------.---------- 1 0 0 0.00025 0.00000 0.00000

CH40.Nef d00 0036 1 R------------------.---------.---------- 1 0 0 0.00025 0.00000 0.00000

CH40.Nef d00 0037 1 -------------------.---------.--------D- 1 1 0 0.00025 0.00013 0.00000

CH40.Nef d00 0038 1 -------------------.-------T-.---------- 1 1 0 0.00025 0.00013 0.00000

CH40.Nef d00 0039 1 --------K----------.---------.---------- 1 1 0 0.00025 0.00013 0.00000

CH40.Nef d00 0041 1 ------------------I.---------.---------- 1 0 0 0.00025 0.00000 0.00000

CH40.Nef d00 0042 1 -------------------.----C----.---------- 1 415 0 0.00025 0.05374 0.00000

CH40.Nef d00 0043 1 -------------------.-----Y---.---------- 1 25 0 0.00025 0.00324 0.00000

CH40.Nef d00 0044 1 -------------------.---------.--R------- 1 3 0 0.00025 0.00039 0.00000

CH40.Nef d00 0045 1 -------------------.---------.---L------ 1 1 0 0.00025 0.00013 0.00000

CH40.Nef d00 0046 1 -L-----------------.-------V-.---------- 1 0 0 0.00025 0.00000 0.00000

CH40.Nef d00 0047 1 ----------A--------.---------.---------- 1 0 0 0.00025 0.00000 0.00000

CH40.Nef d00 0048 1 -------------------.---------.V--------- 1 0 0 0.00025 0.00000 0.00000

CH40.Nef d00 0049 1 -------------------.--E------.---------- 1 0 0 0.00025 0.00000 0.00000

CH40.Nef d00 0050 1 -------------------.--V------.---------- 1 0 0 0.00025 0.00000 0.00000

CH40.Nef d00 0052 1 -------------------.---------.----G----- 1 1 0 0.00025 0.00013 0.00000

CH40.Nef d00 0053 1 -------------------.---------.---S------ 1 0 0 0.00025 0.00000 0.00000

Other day 16 sequences:

CH40.Nef d16 0003 415 -------------------.----C----.---------- 1 415 0 0.00025 0.05374 0.00000

CH40.Nef d16 0009 100 -------------------.I--------.---------- 0 100 0 0.00000 0.01295 0.00000

CH40.Nef d16 0010 51 -------------------.G--------.---------- 2 51 0 0.00049 0.00660 0.00000

CH40.Nef d16 0011 36 -------------------.------A--.---------- 5 36 0 0.00124 0.00466 0.00000

CH40.Nef d16 0012 27 -------------------.---V-----.---------- 0 27 0 0.00000 0.00350 0.00000

CH40.Nef d16 0013 25 -------------------.-----Y---.---------- 1 25 0 0.00025 0.00324 0.00000

CH40.Nef d16 0015 13 -------------------.---C-----.---------- 0 13 0 0.00000 0.00168 0.00000

CH40.Nef d16 0016 12 ------------------G.---------.---------- 4 12 0 0.00099 0.00155 0.00000

CH40.Nef d16 0017 11 ---------------K---.---------.---------- 0 11 0 0.00000 0.00142 0.00000

CH40.Nef d16 0018 9 -----G-------------.---------.---------- 8 9 0 0.00198 0.00117 0.00000

CH40.Nef d16 0019 9 -------------------.---------.-----H---- 0 9 0 0.00000 0.00117 0.00000

CH40.Nef d16 0020 8 -------------------.--V-C----.---------- 0 8 0 0.00000 0.00104 0.00000

CH40.Nef d16 0021 8 -------------------.-P-------.---------- 5 8 0 0.00124 0.00104 0.00000

CH40.Nef d16 0023 6 ----------------L--.---------.---------- 2 6 0 0.00049 0.00078 0.00000

CH40.Nef d16 0024 6 -------------------.-------V-.---------- 4 6 0 0.00099 0.00078 0.00000

CH40.Nef d16 0025 5 -------------------.---------.-P-------- 12 5 0 0.00296 0.00065 0.00000

CH40.Nef d16 0026 5 --Y----------------.---------.---------- 0 5 0 0.00000 0.00065 0.00000

CH40.Nef d16 0028 5 -------------------.----S----.---------- 0 5 0 0.00000 0.00065 0.00000

CH40.Nef d16 0029 5 G------------------.---------.---------- 4 5 0 0.00099 0.00065 0.00000

CH40.Nef d16 0030 4 ---------------K---.--T------.---------- 0 4 0 0.00000 0.00052 0.00000

CH40.Nef d16 0032 4 --------------R----.---------.---------- 2 4 0 0.00049 0.00052 0.00000

CH40.Nef d16 0036 4 -------------------.---------.--------T- 0 4 0 0.00000 0.00052 0.00000

CH40.Nef d16 0037 4 -------------------.---L-----.---------- 2 4 0 0.00049 0.00052 0.00000

CH40.Nef d16 0039 4 -------------------.---------.-----C---- 2 4 0 0.00049 0.00052 0.00000

CH40.Nef d16 0046 3 -------------------.---------.--R------- 1 3 0 0.00025 0.00039 0.00000

CH40.Nef d16 0047 3 ------------S------.---------.---------- 0 3 0 0.00000 0.00039 0.00000

CH40.Nef d16 0049 3 N------------------.---------.---------- 0 3 0 0.00000 0.00039 0.00000

CH40.Nef d16 0050 3 --R----------------.---------.---------- 0 3 0 0.00000 0.00039 0.00000

CH40.Nef d16 0052 3 -------------------.---------.G--------- 2 3 0 0.00049 0.00039 0.00000

CH40.Nef d16 0053 2 -------------------.---------.-Q-------- 1 2 0 0.00025 0.00026 0.00000

CH40.Nef d16 0054 2 --Y----------------.----C----.---------- 0 2 0 0.00000 0.00026 0.00000

CH40.Nef d16 0055 2 -------------------.-R-------.---------- 0 2 0 0.00000 0.00026 0.00000

CH40.Nef d16 0058 2 -------------------.----C----.-P-------- 0 2 0 0.00000 0.00026 0.00000

CH40.Nef d16 0059 2 ----T--------------.---------.---------- 5 2 0 0.00124 0.00026 0.00000

CH40.Nef d16 0061 2 -------------A-----.---------.---------- 0 2 0 0.00000 0.00026 0.00000

CH40.Nef d16 0063 2 -------------------.---------.-------R-- 0 2 0 0.00000 0.00026 0.00000

CH40.Nef d16 0065 2 -----------------G-.---------.---------- 2 2 0 0.00049 0.00026 0.00000

CH40.Nef d16 0066 2 -------------------.---------.--Y------- 0 2 0 0.00000 0.00026 0.00000

CH40.Nef d16 0067 2 ------------------G.I--------.---------- 0 2 0 0.00000 0.00026 0.00000

CH40.Nef d16 0068 2 -R-----------------.---------.---------- 3 2 0 0.00074 0.00026 0.00000

CH40.Nef d16 0070 2 -------------------.G------V-.---------- 0 2 0 0.00000 0.00026 0.00000

CH40.Nef d16 0071 2 -------------------.---------.-------E-- 0 2 0 0.00000 0.00026 0.00000

CH40.Nef d16 0072 2 ------G------------.----C----.---------- 0 2 0 0.00000 0.00026 0.00000

CH40.Nef d16 0075 2 -------L-----------.---------.---------- 0 2 0 0.00000 0.00026 0.00000

CH40.Nef d16 0076 2 -------------------.---------.----K----- 0 2 0 0.00000 0.00026 0.00000

CH40.Nef d16 0077 2 -------------------.---------.--------S- 0 2 0 0.00000 0.00026 0.00000

CH40.Nef d16 0078 2 -------------------.--------P.---------- 0 2 0 0.00000 0.00026 0.00000

CH40.Nef d16 0080 2 ----V--------------.---------.---------- 8 2 0 0.00198 0.00026 0.00000

CH40.Nef d16 0083 2 -------------------.---S-----.---------- 4 2 0 0.00099 0.00026 0.00000

CH40.Nef d16 0084 2 -------------------.-----R---.---------- 2 2 0 0.00049 0.00026 0.00000

CH40.Nef d16 0085 2 -------------------.---------.------H--- 0 2 0 0.00000 0.00026 0.00000

CH40.Nef d16 0092 1 -------------------.----C----.-----H---- 0 1 0 0.00000 0.00013 0.00000

CH40.Nef d16 0095 1 -------------------.--T-C----.---------R 0 1 0 0.00000 0.00013 0.00000

CH40.Nef d16 0096 1 ----------------S--.G--------.---------- 0 1 0 0.00000 0.00013 0.00000

CH40.Nef d16 0099 1 -------------A-----.----C----.---------- 0 1 0 0.00000 0.00013 0.00000

CH40.Nef d16 0106 1 -R-----------------.I--------.---------- 0 1 0 0.00000 0.00013 0.00000

CH40.Nef d16 0110 1 ---------------G---.------A--.---------- 0 1 0 0.00000 0.00013 0.00000

CH40.Nef d16 0111 1 ----R--------------.---C-----.---------- 0 1 0 0.00000 0.00013 0.00000

CH40.Nef d16 0113 1 -------------------.---------.----G----- 1 1 0 0.00025 0.00013 0.00000

CH40.Nef d16 0116 1 -------------------.------G--.---------- 1 1 0 0.00025 0.00013 0.00000

CH40.Nef d16 0119 1 ----I--------------.----C----.---------- 0 1 0 0.00000 0.00013 0.00000

CH40.Nef d16 0123 1 -------------------.D--------.---------- 0 1 0 0.00000 0.00013 0.00000

CH40.Nef d16 0125 1 ---R---------------.---------.---------- 0 1 0 0.00000 0.00013 0.00000

CH40.Nef d16 0131 1 -----------A-------.----C----.---------- 0 1 0 0.00000 0.00013 0.00000

CH40.Nef d16 0132 1 --------K----------.---------.---------- 1 1 0 0.00025 0.00013 0.00000

CH40.Nef d16 0137 1 -------------------.----L----.---------- 0 1 0 0.00000 0.00013 0.00000

CH40.Nef d16 0138 1 ---A---------------.---------.---------- 0 1 0 0.00000 0.00013 0.00000

CH40.Nef d16 0139 1 -------------------.----C----.---L------ 0 1 0 0.00000 0.00013 0.00000

CH40.Nef d16 0142 1 -------------------.I-V------.---------- 0 1 0 0.00000 0.00013 0.00000

CH40.Nef d16 0144 1 ----T-------------G.---------.---------- 0 1 0 0.00000 0.00013 0.00000

CH40.Nef d16 0146 1 -----------A-------.I--------.---------- 0 1 0 0.00000 0.00013 0.00000

CH40.Nef d16 0147 1 --------G----------.----C----.---------- 0 1 0 0.00000 0.00013 0.00000

CH40.Nef d16 0153 1 -----------------G-.----C----.---------- 0 1 0 0.00000 0.00013 0.00000

CH40.Nef d16 0158 1 -------------------.----C----.----V----- 0 1 0 0.00000 0.00013 0.00000

CH40.Nef d16 0161 1 -------------------.---------.--------D- 1 1 0 0.00025 0.00013 0.00000

CH40.Nef d16 0167 1 -------------------.G--------.----G----- 0 1 0 0.00000 0.00013 0.00000

CH40.Nef d16 0174 1 -------------------.I--------.---------R 0 1 0 0.00000 0.00013 0.00000

CH40.Nef d16 0177 1 --R----------------.I--------.---------- 0 1 0 0.00000 0.00013 0.00000

CH40.Nef d16 0181 1 -------------------.----C----.--R------- 0 1 0 0.00000 0.00013 0.00000

CH40.Nef d16 0183 1 --------G----------.---------.---------- 1 1 0 0.00025 0.00013 0.00000

CH40.Nef d16 0187 1 -------------------.-------T-.---------- 1 1 0 0.00025 0.00013 0.00000

CH40.Nef d16 0190 1 ---------------K--G.--T------.---------- 0 1 0 0.00000 0.00013 0.00000

CH40.Nef d16 0192 1 -------------------.I----R---.---------- 0 1 0 0.00000 0.00013 0.00000

CH40.Nef d16 0196 1 ---------------G---.---------.---------- 0 1 0 0.00000 0.00013 0.00000

CH40.Nef d16 0198 1 -------------------.-----N---.---------- 0 1 0 0.00000 0.00013 0.00000

CH40.Nef d16 0199 1 -------------------.---------.---L------ 1 1 0 0.00025 0.00013 0.00000

CH40.Nef d16 0200 1 -------------------.I--------.G--------- 0 1 0 0.00000 0.00013 0.00000

CH40.Nef d16 0201 1 ---E---------------.---------.---------- 0 1 0 0.00000 0.00013 0.00000

CH40.Nef d16 0204 1 -------------------.I--------.-P-------- 0 1 0 0.00000 0.00013 0.00000

There were no additional CH40 sequences at d45 that had not already been grouped above, with transmitted or within-epitope substitutions.

**___________________________________________________________________________________________________________________________________________________**

**SUMA Tat**

**B*1501**

**_________**

**___________**

**__________**

**________**

**SubReg day rank tally Sequence Tally Frequency**

**_______ ___ ____ _____ ______________________________________________________ ___________________ ________________________**

Transmitted PGSQPKTACTTCYCKKCC.FHCQVCFMTKGLGISY.GRKKRRQRRRAPQDSQNH d05 d20 d41 d05 d20 d41

B consensus ----------N-------.-------I--------.----------------T-

Transmitted form:

SumaTAT d05 0001 3246 ------------------.----------------.------------------ 3246 6946 352378 0.99145 0.98821 0.93153

SumaTAT d20 0001 6946 ------------------.----------------.------------------ 3246 6946 352378 0.99145 0.98821 0.93153

SumaTAT d41 0001 352378 ------------------.----------------.------------------ 3246 6946 352378 0.99145 0.98821 0.93153

Most common single mutation by d41 (T/K):

SumaTAT d41 0003 4622 ------------------.--------K-------.------------------ 0 0 4622 0.00000 0.00000 0.01312

SumaTAT d41 0005 561 ------------------.----D---K-------.------------------ 0 0 561 0.00000 0.00000 0.00148

SumaTAT d41 0014 118 -----R------------.--------K-------.------------------ 0 0 118 0.00000 0.00000 0.00031

SumaTAT d41 0046 23 ------------------.--------K-------.-----K------------ 0 0 23 0.00000 0.00000 0.00006

SumaTAT d41 0048 23 ------------------.--------K-------.--------------N--- 0 0 23 0.00000 0.00000 0.00006

SumaTAT d41 0070 15 ------------------.--------K-------.-----------------Y 0 0 15 0.00000 0.00000 0.00004

SumaTAT d41 0139 4 ------------------.--------K-------.----------------I- 0 0 4 0.00000 0.00000 0.00001

SumaTAT d41 0151 3 ------------------.--------KQ------.------------------ 0 0 3 0.00000 0.00000 0.00001

SumaTAT d41 0199 2 -------V----------.--------K-------.------------------ 0 0 2 0.00000 0.00000 0.00001

SumaTAT d41 0211 2 ------------------.----D---K-------.----------------I- 0 0 2 0.00000 0.00000 0.00001

SumaTAT d41 0224 2 ------------------.--------KE------.------------------ 0 0 2 0.00000 0.00000 0.00001

SumaTAT d41 0227 2 ------------------.--------K-------.----W------------- 0 0 2 0.00000 0.00000 0.00001

SumaTAT d41 0235 1 ------------------.--------K-------.--N--------------- 0 0 1 0.00000 0.00000 0.00000

SumaTAT d41 0245 1 ---L--------------.--------K-------.------------------ 0 0 1 0.00000 0.00000 0.00000

SumaTAT d41 0248 1 ------------------.--------K--I----.------------------ 0 0 1 0.00000 0.00000 0.00000

SumaTAT d41 0252 1 -V----------------.--------K-------.------------------ 0 0 1 0.00000 0.00000 0.00000

SumaTAT d41 0285 1 ------------------.------Y-K-------.------------------ 0 0 1 0.00000 0.00000 0.00000

SumaTAT d41 0300 1 Q-----------------.--------K-------.------------------ 0 0 1 0.00000 0.00000 0.00000

SumaTAT d41 0304 1 ------------------.------S-K-------.------------------ 0 0 1 0.00000 0.00000 0.00000

SumaTAT d41 0342 1 ----L-------------.--------K-------.------------------ 0 0 1 0.00000 0.00000 0.00000

SumaTAT d41 0347 1 ------------------.----D---K---A---.------------------ 0 0 1 0.00000 0.00000 0.00000

SumaTAT d41 0349 1 ----------I-------.--------K-------.------------------ 0 0 1 0.00000 0.00000 0.00000

SumaTAT d41 0391 1 ---R--------------.--------K-------.------------------ 0 0 1 0.00000 0.00000 0.00000

SumaTAT d41 0395 1 ------------------.--------K-------.-------------N---- 0 0 1 0.00000 0.00000 0.00000

SumaTAT d41 0402 1 ------------------.---R----K-------.------------------ 0 0 1 0.00000 0.00000 0.00000

SumaTAT d41 0407 1 ------------------.--------K-------.----------T------- 0 0 1 0.00000 0.00000 0.00000

SumaTAT d41 0414 1 S-----------------.--------K-------.------------------ 0 0 1 0.00000 0.00000 0.00000

SumaTAT d41 0419 1 ------------------.--------K-------.D----------------- 0 0 1 0.00000 0.00000 0.00000

Second most common single mutation by d41 (F/L):

SumaTAT d41 0009 204 ------------------.L---------------.------------------ 0 0 204 0.00000 0.00000 0.00054

SumaTAT d41 0255 1 ---------I--------.L---------------.------------------ 0 0 1 0.00000 0.00000 0.00000

SumaTAT d41 0262 1 ------------------.L---------------.----------------D- 0 0 1 0.00000 0.00000 0.00000

SumaTAT d41 0417 1 ------------------.L---------------.----------------S- 0 0 1 0.00000 0.00000 0.00000

Most common form by d41 is a double mutation (F/L, T/K), embedded in several overlapping epitopes:

SumaTAT d41 0002 14721 ------------------.L-------K-------.------------------ 0 0 14721 0.00000 0.00000 0.04175

SumaTAT d41 0021 60 ------------------.L-------K-------.----W------------- 0 0 60 0.00000 0.00000 0.00016

SumaTAT d41 0035 30 ------I-----------.L-------K-------.------------------ 0 0 30 0.00000 0.00000 0.00008

SumaTAT d41 0037 30 ------------------.L-------K-------.-------------G---- 0 0 30 0.00000 0.00000 0.00008

SumaTAT d41 0053 18 ----------P-------.L-------K-------.------------------ 0 0 18 0.00000 0.00000 0.00005

SumaTAT d41 0054 18 ---------------R--.L-------K-------.------------------ 0 0 18 0.00000 0.00000 0.00005

SumaTAT d41 0057 17 ------------------.L-------K-------.----------------I- 0 0 17 0.00000 0.00000 0.00004

SumaTAT d41 0060 17 ------------------.L-------K-------.----------------D- 0 0 17 0.00000 0.00000 0.00004

SumaTAT d41 0076 13 ---------A--------.L-------K-------.------------------ 0 0 13 0.00000 0.00000 0.00003

SumaTAT d41 0081 13 ------------------.L-------K-------.-----------------Y 0 0 13 0.00000 0.00000 0.00003

SumaTAT d41 0104 8 -------V----------.L-------K-------.------------------ 0 0 8 0.00000 0.00000 0.00002

SumaTAT d41 0149 4 ------------H-----.L-------K-------.------------------ 0 0 4 0.00000 0.00000 0.00001

SumaTAT d41 0168 3 ------------------.L-------K-D-----.------------------ 0 0 3 0.00000 0.00000 0.00001

SumaTAT d41 0169 3 ------------------.L-------K-------.--------Q--------- 0 0 3 0.00000 0.00000 0.00001

SumaTAT d41 0182 2 ----S-------------.L-------K-------.------------------ 0 0 2 0.00000 0.00000 0.00001

SumaTAT d41 0184 2 ------------------.L---I---K-------.------------------ 0 0 2 0.00000 0.00000 0.00001

SumaTAT d41 0197 2 ------------------.L-------K-------.----Q------------- 0 0 2 0.00000 0.00000 0.00001

SumaTAT d41 0200 2 ------------------.L-------K-------.--E--------------- 0 0 2 0.00000 0.00000 0.00001

SumaTAT d41 0202 2 ------------------.L-------K-------.--------------N--- 0 0 2 0.00000 0.00000 0.00001

SumaTAT d41 0209 2 ---------I--------.L-------K-------.------------------ 0 0 2 0.00000 0.00000 0.00001

SumaTAT d41 0219 2 ------------------.L-------K-------.-------------E---- 0 0 2 0.00000 0.00000 0.00001

SumaTAT d41 0222 2 ------------------.L-------K-------.-----------L------ 0 0 2 0.00000 0.00000 0.00001

SumaTAT d41 0229 2 ---------S--------.L-------K-------.------------------ 0 0 2 0.00000 0.00000 0.00001

SumaTAT d41 0231 2 ------------------.L-------K-------.-----------------N 0 0 2 0.00000 0.00000 0.00001

SumaTAT d41 0233 1 -------P----------.L-------K-------.------------------ 0 0 1 0.00000 0.00000 0.00000

SumaTAT d41 0236 1 ------------------.L----S--K-------.------------------ 0 0 1 0.00000 0.00000 0.00000

SumaTAT d41 0237 1 -----------Y------.L-------K-------.------------------ 0 0 1 0.00000 0.00000 0.00000

SumaTAT d41 0238 1 ------------------.L-------A-------.------------------ 0 0 1 0.00000 0.00000 0.00000

SumaTAT d41 0244 1 ------------------.L-------K-C-----.------------------ 0 0 1 0.00000 0.00000 0.00000

SumaTAT d41 0249 1 ------------------.L-------K-------.-------------N---- 0 0 1 0.00000 0.00000 0.00000

SumaTAT d41 0268 1 ------------------.L-------K------C.------------------ 0 0 1 0.00000 0.00000 0.00000

SumaTAT d41 0289 1 ------------------.L---F---K-------.------------------ 0 0 1 0.00000 0.00000 0.00000

SumaTAT d41 0295 1 ------------------.L-------K-------.---M-------------- 0 0 1 0.00000 0.00000 0.00000

SumaTAT d41 0297 1 ---------P--------.L-------K-------.------------------ 0 0 1 0.00000 0.00000 0.00000

SumaTAT d41 0299 1 -----M------------.L-------K-------.------------------ 0 0 1 0.00000 0.00000 0.00000

SumaTAT d41 0302 1 ----L-------------.L-------K-------.------------------ 0 0 1 0.00000 0.00000 0.00000

SumaTAT d41 0308 1 -------------Y----.L-------K-------.------------------ 0 0 1 0.00000 0.00000 0.00000

SumaTAT d41 0312 1 ------------------.L-----L-K-------.------------------ 0 0 1 0.00000 0.00000 0.00000

SumaTAT d41 0313 1 ------------------.L-S-----K-------.------------------ 0 0 1 0.00000 0.00000 0.00000

SumaTAT d41 0318 1 -E----------------.L-------K-------.------------------ 0 0 1 0.00000 0.00000 0.00000

SumaTAT d41 0320 1 -------T----------.L-------K-------.------------------ 0 0 1 0.00000 0.00000 0.00000

SumaTAT d41 0323 1 ----A-------------.L-------K-------.------------------ 0 0 1 0.00000 0.00000 0.00000

SumaTAT d41 0324 1 ------------------.L-R-----K-------.------------------ 0 0 1 0.00000 0.00000 0.00000

SumaTAT d41 0331 1 ------------------.L-------K-------.-----K------------ 0 0 1 0.00000 0.00000 0.00000

SumaTAT d41 0332 1 ------------------.L-------K-------.---N-------------- 0 0 1 0.00000 0.00000 0.00000

SumaTAT d41 0333 1 ----H-------------.L-------K-------.------------------ 0 0 1 0.00000 0.00000 0.00000

SumaTAT d41 0334 1 L-----------------.L-------K-------.------------------ 0 0 1 0.00000 0.00000 0.00000

SumaTAT d41 0339 1 ----------------S-.L-------K-------.------------------ 0 0 1 0.00000 0.00000 0.00000

SumaTAT d41 0340 1 ------------------.L-------K-------.--------------T--- 0 0 1 0.00000 0.00000 0.00000

SumaTAT d41 0348 1 ------------------.L-------K-------.-W---------------- 0 0 1 0.00000 0.00000 0.00000

SumaTAT d41 0350 1 ------------------.L-------K-------.V----------------- 0 0 1 0.00000 0.00000 0.00000

SumaTAT d41 0353 1 ------------------.L--H----K-------.------------------ 0 0 1 0.00000 0.00000 0.00000

SumaTAT d41 0354 1 ------------------.L----Y--K-------.------------------ 0 0 1 0.00000 0.00000 0.00000

SumaTAT d41 0359 1 ------------------.L-------K-------.---------------K-- 0 0 1 0.00000 0.00000 0.00000

SumaTAT d41 0367 1 ------A-----------.L-------K-------.------------------ 0 0 1 0.00000 0.00000 0.00000

SumaTAT d41 0371 1 ------------------.L-------K---S---.------------------ 0 0 1 0.00000 0.00000 0.00000

SumaTAT d41 0372 1 --T---------------.L-------K-------.------------------ 0 0 1 0.00000 0.00000 0.00000

SumaTAT d41 0374 1 ------------------.L-------K-------.--------L--------- 0 0 1 0.00000 0.00000 0.00000

SumaTAT d41 0376 1 ------------------.L-------K-------.--------------I--- 0 0 1 0.00000 0.00000 0.00000

SumaTAT d41 0377 1 ------------------.L-------K-------.------L----------- 0 0 1 0.00000 0.00000 0.00000

SumaTAT d41 0381 1 ------------------.L-Y-----K-------.------------------ 0 0 1 0.00000 0.00000 0.00000

SumaTAT d41 0383 1 ------------------.L-------K---D---.------------------ 0 0 1 0.00000 0.00000 0.00000

SumaTAT d41 0384 1 ------------------.L-------K-------.------H----------- 0 0 1 0.00000 0.00000 0.00000

SumaTAT d41 0387 1 --I---------------.L-------K-------.------------------ 0 0 1 0.00000 0.00000 0.00000

SumaTAT d41 0396 1 ------------------.L-------K-------.-S---------------- 0 0 1 0.00000 0.00000 0.00000

SumaTAT d41 0397 1 S-----------------.L-------K-------.------------------ 0 0 1 0.00000 0.00000 0.00000

SumaTAT d41 0398 1 ------------------.L-------K--F----.------------------ 0 0 1 0.00000 0.00000 0.00000

SumaTAT d41 0405 1 ------------------.L-----S-K-------.------------------ 0 0 1 0.00000 0.00000 0.00000

SumaTAT d41 0406 1 ----------------Y-.L-------K-------.------------------ 0 0 1 0.00000 0.00000 0.00000

SumaTAT d41 0410 1 ------------------.L-------K-------.---------S-------- 0 0 1 0.00000 0.00000 0.00000

SumaTAT d41 0412 1 -------------R----.L-------K-------.------------------ 0 0 1 0.00000 0.00000 0.00000

SumaTAT d41 0413 1 ------------------.L------IK-------.------------------ 0 0 1 0.00000 0.00000 0.00000

SumaTAT d41 0416 1 ------------------.L-------K-------.----------------S- 0 0 1 0.00000 0.00000 0.00000

Other day 05 sequences:

SumaTAT d05 0002 3 ------------------.----------------.-------Q---------- 3 0 50 0.00092 0.00000 0.00013

SumaTAT d05 0003 2 ------------------.----------------.----------------Y- 2 0 1 0.00061 0.00000 0.00000

SumaTAT d05 0004 2 ----------I-------.----------------.------------------ 2 7 13 0.00061 0.00099 0.00003

SumaTAT d05 0005 1 ------I-----------.----------------.------------------ 1 0 14 0.00031 0.00000 0.00004

SumaTAT d05 0006 1 ---------------R--.----------------.--------------R--- 1 0 0 0.00031 0.00000 0.00000

SumaTAT d05 0007 1 ------------------.-N--------------.------------------ 1 0 3 0.00031 0.00000 0.00001

SumaTAT d05 0008 1 ------N-----------.----------------.------------------ 1 0 5 0.00031 0.00000 0.00001

SumaTAT d05 0010 1 ------------------.-----Y----------.------------------ 1 0 2 0.00031 0.00000 0.00001

SumaTAT d05 0011 1 ------------------.-------------V--.------------------ 1 2 0 0.00031 0.00028 0.00000

SumaTAT d05 0012 1 ------------------.----------------.----------T------- 1 0 229 0.00031 0.00000 0.00061

SumaTAT d05 0013 1 ------------------.----------------.----------S------- 1 0 2 0.00031 0.00000 0.00001

SumaTAT d05 0014 1 ------------------.------------S---.------------------ 1 2 13 0.00031 0.00028 0.00003

SumaTAT d05 0015 1 ----L-------------.----------------.------------------ 1 0 12 0.00031 0.00000 0.00003

SumaTAT d05 0016 1 ------------------.----------------.-----K------------ 1 2 174 0.00031 0.00028 0.00046

SumaTAT d05 0017 1 ------------------.-------I--------.------------------ 1 2 16 0.00031 0.00028 0.00004

SumaTAT d05 0019 1 --N---------------.----------------.------------------ 1 1 9 0.00031 0.00014 0.00002

SumaTAT d05 0020 1 ------------------.----------------.--------------N--- 1 0 37 0.00031 0.00000 0.00010

SumaTAT d05 0021 1 ------A-----------.----------------.------------------ 1 0 6 0.00031 0.00000 0.00002

SumaTAT d05 0022 1 ------------------.--------I-------.------------------ 1 0 8 0.00031 0.00000 0.00002

SumaTAT d05 0023 1 -----------------Y.----------------.------------------ 1 1 14 0.00031 0.00014 0.00004

SumaTAT d05 0024 1 ------------------.----------------.-K---------------- 1 1 7 0.00031 0.00014 0.00002

Other day 20 sequences:

SumaTAT d20 0002 12 ------------------.----------------.----------------I- 0 12 750 0.00000 0.00170 0.00213

SumaTAT d20 0003 7 ----------I-------.----------------.------------------ 2 7 13 0.00061 0.00099 0.00003

SumaTAT d20 0004 4 ------------------.--------------F-.------------------ 0 4 11 0.00000 0.00057 0.00003

SumaTAT d20 0005 2 ------------------.----------------.--------------C--- 0 2 1 0.00000 0.00028 0.00000

SumaTAT d20 0006 2 ------------------.----------------.-----------------Q 0 2 5 0.00000 0.00028 0.00001

SumaTAT d20 0007 2 ------------------.-------------V--.------------------ 1 2 0 0.00031 0.00028 0.00000

SumaTAT d20 0008 2 ---------------R--.----------------.------------------ 0 2 123 0.00000 0.00028 0.00033

SumaTAT d20 0009 2 ------------------.----------------.----------------T- 0 2 9 0.00000 0.00028 0.00002

SumaTAT d20 0010 2 ------------------.----------------.-----K------------ 1 2 174 0.00031 0.00028 0.00046

SumaTAT d20 0011 2 ------------------.----------------.--------Q--------- 0 2 13 0.00000 0.00028 0.00003

SumaTAT d20 0012 2 ------------------.------------S---.------------------ 1 2 13 0.00031 0.00028 0.00003

SumaTAT d20 0013 2 ------------------.-------I--------.------------------ 1 2 16 0.00031 0.00028 0.00004

SumaTAT d20 0014 2 ------------------.----------------.----W------------- 0 2 30 0.00000 0.00028 0.00008

SumaTAT d20 0015 2 ------------------.----I-----------.------------------ 0 2 9 0.00000 0.00028 0.00002

SumaTAT d20 0016 1 ------------------.-Q--------------.------------------ 0 1 1 0.00000 0.00014 0.00000

SumaTAT d20 0017 1 ------------F-----.----------------.------------------ 0 1 1 0.00000 0.00014 0.00000

SumaTAT d20 0018 1 L-----------------.----------------.------------------ 0 1 16 0.00000 0.00014 0.00004

SumaTAT d20 0019 1 --------Y---------.----------------.------------------ 0 1 8 0.00000 0.00014 0.00002

SumaTAT d20 0020 1 ------------------.----------------.V----------------- 0 1 6 0.00000 0.00014 0.00002

SumaTAT d20 0021 1 ------------------.----------------.-----------T------ 0 1 5 0.00000 0.00014 0.00001

SumaTAT d20 0023 1 ------------------.-----------F----.------------------ 0 1 0 0.00000 0.00014 0.00000

SumaTAT d20 0024 1 ------------------.----------------.-M---------------- 0 1 3 0.00000 0.00014 0.00001

SumaTAT d20 0026 1 -R----------------.----------------.------------------ 0 1 15 0.00000 0.00014 0.00004

SumaTAT d20 0030 1 ------------------.----A-----------.------------------ 0 1 3 0.00000 0.00014 0.00001

SumaTAT d20 0031 1 R-----------------.----------------.------------------ 0 1 0 0.00000 0.00014 0.00000

SumaTAT d20 0032 1 -----R------------.----------------.------------------ 0 1 488 0.00000 0.00014 0.00129

SumaTAT d20 0034 1 ---------A--------.----------------.------------------ 0 1 15 0.00000 0.00014 0.00004

SumaTAT d20 0035 1 ----------------S-.----------------.------------------ 0 1 0 0.00000 0.00014 0.00000

SumaTAT d20 0036 1 ------------------.----------------.-----------H------ 0 1 12 0.00000 0.00014 0.00003

SumaTAT d20 0038 1 ------------------.----------------.-----------S------ 0 1 101 0.00000 0.00014 0.00027

SumaTAT d20 0039 1 ------------------.----------------.----------------S- 0 1 444 0.00000 0.00014 0.00117

SumaTAT d20 0041 1 ------------------.---------E------.------------------ 0 1 0 0.00000 0.00014 0.00000

SumaTAT d20 0042 1 ------------------.----------------.----------V------- 0 1 58 0.00000 0.00014 0.00015

SumaTAT d20 0043 1 ------------------.----------------.---------K-------- 0 1 8 0.00000 0.00014 0.00002

SumaTAT d20 0044 1 ----------A-------.----------------.------------------ 0 1 6 0.00000 0.00014 0.00002

SumaTAT d20 0046 1 ------------------.----------------.----Q------------- 0 1 26 0.00000 0.00014 0.00007

SumaTAT d20 0047 1 ------------------.----------------.-------------G---- 0 1 178 0.00000 0.00014 0.00047

SumaTAT d20 0048 1 ------------N-----.----------------.------------------ 0 1 0 0.00000 0.00014 0.00000

SumaTAT d20 0050 1 ------------------.--Y-------------.------------------ 0 1 17 0.00000 0.00014 0.00004

SumaTAT d20 0052 1 ------------------.----------------.------------H----- 0 1 7 0.00000 0.00014 0.00002

SumaTAT d20 0053 1 --N---------------.----------------.------------------ 1 1 9 0.00031 0.00014 0.00002

SumaTAT d20 0054 1 -----------------Y.----------------.------------------ 1 1 14 0.00031 0.00014 0.00004

SumaTAT d20 0055 1 ------------------.----------------.-K---------------- 1 1 7 0.00031 0.00014 0.00002

SumaTAT d20 0056 1 ----------------G-.----------------.------------------ 0 1 0 0.00000 0.00014 0.00000

SumaTAT d20 0057 1 --G---------------.----------------.------------------ 0 1 1 0.00000 0.00014 0.00000

SumaTAT d20 0058 1 ----S-------------.----------------.------------------ 0 1 142 0.00000 0.00014 0.00038

SumaTAT d20 0059 1 ------------------.---K------------.------------------ 0 1 2 0.00000 0.00014 0.00001

Other day 41 sequences:

SumaTAT d41 0004 750 ------------------.----------------.----------------I- 0 12 750 0.00000 0.00170 0.00213

SumaTAT d41 0006 488 -----R------------.----------------.------------------ 0 1 488 0.00000 0.00014 0.00129

SumaTAT d41 0007 444 ------------------.----------------.----------------S- 0 1 444 0.00000 0.00014 0.00117

SumaTAT d41 0008 230 ------------------.----------------.----------T------- 1 0 230 0.00031 0.00000 0.00061

SumaTAT d41 0010 178 ------------------.----------------.-------------G---- 0 1 178 0.00000 0.00014 0.00047

SumaTAT d41 0011 174 ------------------.----------------.-----K------------ 1 2 174 0.00031 0.00028 0.00046

SumaTAT d41 0012 142 ----S-------------.----------------.------------------ 0 1 142 0.00000 0.00014 0.00038

SumaTAT d41 0013 123 ---------------R--.----------------.------------------ 0 2 123 0.00000 0.00028 0.00033

SumaTAT d41 0015 101 ------------------.----------------.-----------S------ 0 1 101 0.00000 0.00014 0.00027

SumaTAT d41 0017 78 ------------------.----------------.-----------------Y 0 0 78 0.00000 0.00000 0.00021

SumaTAT d41 0018 74 ------------------.Y---------------.------------------ 0 0 74 0.00000 0.00000 0.00020

SumaTAT d41 0019 67 ------------------.C-------K-------.------------------ 0 0 67 0.00000 0.00000 0.00018

SumaTAT d41 0020 65 ------------------.-------V--------.------------------ 0 0 65 0.00000 0.00000 0.00017

SumaTAT d41 0022 59 ------------------.----------------.----------------D- 0 0 59 0.00000 0.00000 0.00016

SumaTAT d41 0023 58 ------------------.----------------.----------V------- 0 1 58 0.00000 0.00014 0.00015

SumaTAT d41 0024 58 S-----------------.----------------.------------------ 0 0 58 0.00000 0.00000 0.00015

SumaTAT d41 0025 50 ------------------.----------------.-------Q---------- 3 0 50 0.00092 0.00000 0.00013

SumaTAT d41 0026 48 ------------H-----.----------------.------------------ 0 0 48 0.00000 0.00000 0.00013

SumaTAT d41 0027 47 ------------------.----------------.-----------L------ 0 0 47 0.00000 0.00000 0.00012

SumaTAT d41 0028 41 ------------------.----------------.--R--------------- 0 0 41 0.00000 0.00000 0.00011

SumaTAT d41 0029 40 -------D----------.----------------.------------------ 0 0 40 0.00000 0.00000 0.00011

SumaTAT d41 0030 40 ------------------.-------T--------.------------------ 0 0 40 0.00000 0.00000 0.00011

SumaTAT d41 0031 38 ------------------.----------------.------------R----- 0 0 38 0.00000 0.00000 0.00010

SumaTAT d41 0032 38 ------------------.----------------.--------------N--- 1 0 38 0.00031 0.00000 0.00010

SumaTAT d41 0033 35 ------------------.----------S-----.------------------ 0 0 35 0.00000 0.00000 0.00009

SumaTAT d41 0034 34 ------------------.--------A-------.------------------ 0 0 34 0.00000 0.00000 0.00009

SumaTAT d41 0036 30 ------------------.----------------.----W------------- 0 2 30 0.00000 0.00028 0.00008

SumaTAT d41 0038 30 ---R--------------.----------------.------------------ 0 0 30 0.00000 0.00000 0.00008

SumaTAT d41 0039 29 ------------------.-----------S----.------------------ 0 0 29 0.00000 0.00000 0.00008

SumaTAT d41 0040 28 -------T----------.----------------.------------------ 0 0 28 0.00000 0.00000 0.00007

SumaTAT d41 0041 27 ------------------.----------------.---R-------------- 0 0 27 0.00000 0.00000 0.00007

SumaTAT d41 0042 27 ------------------.V---------------.------------------ 0 0 27 0.00000 0.00000 0.00007

SumaTAT d41 0044 26 ------------------.----------------.----Q------------- 0 1 26 0.00000 0.00014 0.00007

SumaTAT d41 0045 23 -----------G------.----------------.------------------ 0 0 23 0.00000 0.00000 0.00006

SumaTAT d41 0049 20 ------------------.----------------.---------------L-- 0 0 20 0.00000 0.00000 0.00005

SumaTAT d41 0050 20 ------------------.----------------.--------------G--- 0 0 20 0.00000 0.00000 0.00005

SumaTAT d41 0051 20 ------------------.----------------.------------K----- 0 0 20 0.00000 0.00000 0.00005

SumaTAT d41 0052 19 ------------------.----------------.-------------N---- 0 0 19 0.00000 0.00000 0.00005

SumaTAT d41 0055 18 ------------------.S---------------.------------------ 0 0 18 0.00000 0.00000 0.00005

SumaTAT d41 0056 17 --------S---------.----------------.------------------ 0 0 17 0.00000 0.00000 0.00004

SumaTAT d41 0058 17 ------------------.---------------H.------------------ 0 0 17 0.00000 0.00000 0.00004

SumaTAT d41 0059 17 ------------------.--Y-------------.------------------ 0 1 17 0.00000 0.00014 0.00004

SumaTAT d41 0061 16 ------------------.--------------P-.------------------ 0 0 16 0.00000 0.00000 0.00004

SumaTAT d41 0062 16 L-----------------.----------------.------------------ 0 1 16 0.00000 0.00014 0.00004

SumaTAT d41 0063 16 ------------------.-------I--------.------------------ 1 2 16 0.00031 0.00028 0.00004

SumaTAT d41 0064 15 -R----------------.----------------.------------------ 0 1 15 0.00000 0.00014 0.00004

SumaTAT d41 0065 15 ------------------.------------D---.------------------ 0 0 15 0.00000 0.00000 0.00004

SumaTAT d41 0066 15 -------------R----.----------------.------------------ 0 0 15 0.00000 0.00000 0.00004

SumaTAT d41 0067 15 ------------------.----------D-----.------------------ 0 0 15 0.00000 0.00000 0.00004

SumaTAT d41 0069 15 ---------A--------.----------------.------------------ 0 1 15 0.00000 0.00014 0.00004

SumaTAT d41 0071 14 -------------Y----.----------------.------------------ 0 0 14 0.00000 0.00000 0.00004

SumaTAT d41 0072 14 -----------------Y.----------------.------------------ 1 1 14 0.00031 0.00014 0.00004

SumaTAT d41 0073 14 -------V----------.----------------.------------------ 0 0 14 0.00000 0.00000 0.00004

SumaTAT d41 0074 14 ------------------.----------------.----------------H- 0 0 14 0.00000 0.00000 0.00004

SumaTAT d41 0075 14 ------I-----------.----------------.------------------ 1 0 14 0.00031 0.00000 0.00004

SumaTAT d41 0078 13 ---------I--------.----------------.------------------ 0 0 13 0.00000 0.00000 0.00003

SumaTAT d41 0079 13 ----------I-------.----------------.------------------ 2 7 13 0.00061 0.00099 0.00003

SumaTAT d41 0082 13 ------------------.----------------.--------Q--------- 0 2 13 0.00000 0.00028 0.00003

SumaTAT d41 0084 13 ------------------.------------S---.------------------ 1 2 13 0.00031 0.00028 0.00003

SumaTAT d41 0085 12 ------------------.----------------.---N-------------- 0 0 12 0.00000 0.00000 0.00003

SumaTAT d41 0086 12 ------------------.----------------.-----------H------ 0 1 12 0.00000 0.00014 0.00003

SumaTAT d41 0087 12 -E----------------.----------------.------------------ 0 0 12 0.00000 0.00000 0.00003

SumaTAT d41 0088 12 ----L-------------.----------------.------------------ 1 0 12 0.00031 0.00000 0.00003

SumaTAT d41 0089 11 ------------------.----------------.D----------------- 0 0 11 0.00000 0.00000 0.00003

SumaTAT d41 0090 11 T-----------------.----------------.------------------ 0 0 11 0.00000 0.00000 0.00003

SumaTAT d41 0091 11 ------------------.--------------F-.------------------ 0 4 11 0.00000 0.00057 0.00003

SumaTAT d41 0093 10 ------------------.----------------.------R----------- 0 0 10 0.00000 0.00000 0.00003

SumaTAT d41 0092 9 ----------------Y-.----------------.------------------ 0 0 9 0.00000 0.00000 0.00002

SumaTAT d41 0080 9 ------------------.----------------.----------------T- 0 2 9 0.00000 0.00028 0.00002

SumaTAT d41 0094 9 ------------------.-Y--------------.------------------ 0 0 9 0.00000 0.00000 0.00002

SumaTAT d41 0095 9 --N---------------.----------------.------------------ 1 1 9 0.00031 0.00014 0.00002

SumaTAT d41 0096 9 ------------------.----------------.------------P----- 0 0 9 0.00000 0.00000 0.00002

SumaTAT d41 0097 9 ------------------.----I-----------.------------------ 0 2 9 0.00000 0.00028 0.00002

SumaTAT d41 0098 8 ------------------.----------------.---------K-------- 0 1 8 0.00000 0.00014 0.00002

SumaTAT d41 0099 8 ------------------.----------------.------H----------- 0 0 8 0.00000 0.00000 0.00002

SumaTAT d41 0100 8 ------------------.--------I-------.------------------ 1 0 8 0.00031 0.00000 0.00002

SumaTAT d41 0101 8 ------------------.----------------.--E--------------- 0 0 8 0.00000 0.00000 0.00002

SumaTAT d41 0102 8 --------Y---------.----------------.------------------ 0 1 8 0.00000 0.00014 0.00002

SumaTAT d41 0105 7 ------------------.----------------.-K---------------- 1 1 7 0.00031 0.00014 0.00002

SumaTAT d41 0108 7 ------------------.----------------.--------------T--- 0 0 7 0.00000 0.00000 0.00002

SumaTAT d41 0110 7 ------------------.----------------.------------H----- 0 1 7 0.00000 0.00014 0.00002

SumaTAT d41 0112 7 ------------------.--S-------------.------------------ 0 0 7 0.00000 0.00000 0.00002

SumaTAT d41 0113 7 ------------------.----------------.---------G-------- 0 0 7 0.00000 0.00000 0.00002

SumaTAT d41 0109 6 ------------------.----------------.--------------R--- 0 0 6 0.00000 0.00000 0.00002

SumaTAT d41 0114 6 ------A-----------.----------------.------------------ 1 0 6 0.00031 0.00000 0.00002

SumaTAT d41 0115 6 ------------------.----------------.--N--------------- 0 0 6 0.00000 0.00000 0.00002

SumaTAT d41 0118 6 ------------------.----------------.V----------------- 0 1 6 0.00000 0.00014 0.00002

SumaTAT d41 0120 6 ------------------.----F-----------.------------------ 0 0 6 0.00000 0.00000 0.00002

SumaTAT d41 0121 6 ----------A-------.----------------.------------------ 0 1 6 0.00000 0.00014 0.00002

SumaTAT d41 0122 6 ------------------.--F-------------.------------------ 0 0 6 0.00000 0.00000 0.00002

SumaTAT d41 0123 6 ------------------.----------------.-------G---------- 0 0 6 0.00000 0.00000 0.00002

SumaTAT d41 0125 5 ------------------.----------------.-----------------Q 0 2 5 0.00000 0.00028 0.00001

SumaTAT d41 0127 5 ------------------.----D-----------.------------------ 0 0 5 0.00000 0.00000 0.00001

SumaTAT d41 0128 5 ------------------.----------------.----L------------- 0 0 5 0.00000 0.00000 0.00001

SumaTAT d41 0129 5 -----------Y------.----------------.------------------ 0 0 5 0.00000 0.00000 0.00001

SumaTAT d41 0130 5 ----A-------------.----------------.------------------ 0 0 5 0.00000 0.00000 0.00001

SumaTAT d41 0131 5 ------N-----------.----------------.------------------ 1 0 5 0.00031 0.00000 0.00001

SumaTAT d41 0132 5 ------------------.----------------.S----------------- 0 0 5 0.00000 0.00000 0.00001

SumaTAT d41 0133 5 ------------------.----------------.-----------T------ 0 1 5 0.00000 0.00014 0.00001

SumaTAT d41 0135 5 ---K--------------.----------------.------------------ 0 0 5 0.00000 0.00000 0.00001

SumaTAT d41 0134 4 ------------------.----------------.-----------------N 0 0 4 0.00000 0.00000 0.00001

SumaTAT d41 0119 4 -------------S----.----------------.------------------ 0 0 4 0.00000 0.00000 0.00001

SumaTAT d41 0136 4 ------------------.----------------.--------L--------- 0 0 4 0.00000 0.00000 0.00001

SumaTAT d41 0138 4 ------------------.------------A---.------------------ 0 0 4 0.00000 0.00000 0.00001

SumaTAT d41 0141 4 --I---------------.----------------.------------------ 0 0 4 0.00000 0.00000 0.00001

SumaTAT d41 0142 4 ------------------.--R-------------.------------------ 0 0 4 0.00000 0.00000 0.00001

SumaTAT d41 0143 4 ------------------.---------------C.------------------ 0 0 4 0.00000 0.00000 0.00001

SumaTAT d41 0144 4 ------------------.------L---------.------------------ 0 0 4 0.00000 0.00000 0.00001

SumaTAT d41 0145 4 ------------------.----------------.-----I------------ 0 0 4 0.00000 0.00000 0.00001

SumaTAT d41 0146 4 ------------------.------------V---.------------------ 0 0 4 0.00000 0.00000 0.00001

SumaTAT d41 0147 4 ------------------.----------------.-W---------------- 0 0 4 0.00000 0.00000 0.00001

SumaTAT d41 0148 4 ------------------.---------R------.------------------ 0 0 4 0.00000 0.00000 0.00001

SumaTAT d41 0150 3 ------------------.----A-----------.------------------ 0 1 3 0.00000 0.00014 0.00001

SumaTAT d41 0153 3 ------------------.----------------.-----T------------ 0 0 3 0.00000 0.00000 0.00001

SumaTAT d41 0154 3 ------------------.----------------.----G------------- 0 0 3 0.00000 0.00000 0.00001

SumaTAT d41 0155 3 ------------------.----------------.-------------Y---- 0 0 3 0.00000 0.00000 0.00001

SumaTAT d41 0156 3 ---------N--------.----------------.------------------ 0 0 3 0.00000 0.00000 0.00001

SumaTAT d41 0157 3 ---H--------------.----------------.------------------ 0 0 3 0.00000 0.00000 0.00001

SumaTAT d41 0158 3 -----M------------.----------------.------------------ 0 0 3 0.00000 0.00000 0.00001

SumaTAT d41 0159 3 Q-----------------.----------------.------------------ 0 0 3 0.00000 0.00000 0.00001

SumaTAT d41 0160 3 -------------F----.----------------.------------------ 0 0 3 0.00000 0.00000 0.00001

SumaTAT d41 0161 3 ------------------.----------V-----.------------------ 0 0 3 0.00000 0.00000 0.00001

SumaTAT d41 0162 3 ------------------.--------------T-.------------------ 0 0 3 0.00000 0.00000 0.00001

SumaTAT d41 0163 3 ------------------.---R------------.------------------ 0 0 3 0.00000 0.00000 0.00001

SumaTAT d41 0164 3 ------------------.------------C---.------------------ 0 0 3 0.00000 0.00000 0.00001

SumaTAT d41 0165 3 ------------------.-N--------------.------------------ 1 0 3 0.00031 0.00000 0.00001

SumaTAT d41 0166 3 ------------------.----------------.---------------R-- 0 0 3 0.00000 0.00000 0.00001

SumaTAT d41 0167 3 -----------R------.----------------.------------------ 0 0 3 0.00000 0.00000 0.00001

SumaTAT d41 0170 3 ------------------.----------------.-S---------------- 0 0 3 0.00000 0.00000 0.00001

SumaTAT d41 0171 3 ------------------.----------------.-M---------------- 0 1 3 0.00000 0.00014 0.00001

SumaTAT d41 0172 3 ------------------.-----F----------.------------------ 0 0 3 0.00000 0.00000 0.00001

SumaTAT d41 0173 3 ------------------.----------------.C----------------- 0 0 3 0.00000 0.00000 0.00001

SumaTAT d41 0174 3 A-----------------.----------------.------------------ 0 0 3 0.00000 0.00000 0.00001

SumaTAT d41 0175 3 ------------------.----------------.-------------S---- 0 0 3 0.00000 0.00000 0.00001

SumaTAT d41 0176 3 --------R---------.----------------.------------------ 0 0 3 0.00000 0.00000 0.00001

SumaTAT d41 0177 3 ---------------N--.----------------.------------------ 0 0 3 0.00000 0.00000 0.00001

SumaTAT d41 0179 3 ------------------.----------------.-----G------------ 0 0 3 0.00000 0.00000 0.00001

SumaTAT d41 0126 2 ------------------.----------------.A----------------- 0 0 2 0.00000 0.00000 0.00001

SumaTAT d41 0180 2 ------------------.----------------.---------I-------- 0 0 2 0.00000 0.00000 0.00001

SumaTAT d41 0181 2 R-----------------.----------------.------------------ 0 0 2 0.00000 0.00000 0.00001

SumaTAT d41 0183 2 ------------------.----------------.---E-------------- 0 0 2 0.00000 0.00000 0.00001

SumaTAT d41 0185 2 ---------S--------.----------------.------------------ 0 0 2 0.00000 0.00000 0.00001

SumaTAT d41 0187 2 ------------------.----------------.-------------H---- 0 0 2 0.00000 0.00000 0.00001

SumaTAT d41 0188 2 ------------------.----------------.-------Q--T------- 0 0 2 0.00000 0.00000 0.00001

SumaTAT d41 0189 2 ------------------.------S---------.------------------ 0 0 2 0.00000 0.00000 0.00001

SumaTAT d41 0190 2 -RR---------------.----------------.------------------ 0 0 2 0.00000 0.00000 0.00001

SumaTAT d41 0191 2 -V----------------.----------------.------------------ 0 0 2 0.00000 0.00000 0.00001

SumaTAT d41 0192 2 ------------------.---------N------.------------------ 0 0 2 0.00000 0.00000 0.00001

SumaTAT d41 0193 2 ----------S-------.----------------.------------------ 0 0 2 0.00000 0.00000 0.00001

SumaTAT d41 0194 2 ------------------.----------------.----------S------- 1 0 2 0.00031 0.00000 0.00001

SumaTAT d41 0195 2 ------------------.----------------.-------------E---- 0 0 2 0.00000 0.00000 0.00001

SumaTAT d41 0196 2 ------------------.----------------.-G---------------- 0 0 2 0.00000 0.00000 0.00001

SumaTAT d41 0198 2 -----N------------.----------------.------------------ 0 0 2 0.00000 0.00000 0.00001

SumaTAT d41 0201 2 ---HA-------------.----------------.------------------ 0 0 2 0.00000 0.00000 0.00001

SumaTAT d41 0205 2 ------------------.--------S-------.------------------ 0 0 2 0.00000 0.00000 0.00001

SumaTAT d41 0206 2 ------------------.----------------.----------P------- 0 0 2 0.00000 0.00000 0.00001

SumaTAT d41 0207 2 ------------------.---K------------.------------------ 0 1 2 0.00000 0.00014 0.00001

SumaTAT d41 0208 2 ------------------.----------------.----------D------- 0 0 2 0.00000 0.00000 0.00001

SumaTAT d41 0210 2 --------------E---.----------------.------------------ 0 0 2 0.00000 0.00000 0.00001

SumaTAT d41 0212 2 ------------------.I---------------.------------------ 0 0 2 0.00000 0.00000 0.00001

SumaTAT d41 0213 2 -------S----------.----------------.------------------ 0 0 2 0.00000 0.00000 0.00001

SumaTAT d41 0214 2 ----------N-------.----------------.------------------ 0 0 2 0.00000 0.00000 0.00001

SumaTAT d41 0215 2 -------------G----.----------------.------------------ 0 0 2 0.00000 0.00000 0.00001

SumaTAT d41 0216 2 ------------------.----L-----------.------------------ 0 0 2 0.00000 0.00000 0.00001

SumaTAT d41 0217 2 ------------------.-----Y----------.------------------ 1 0 2 0.00031 0.00000 0.00001

SumaTAT d41 0218 2 ------------C-----.----------------.------------------ 0 0 2 0.00000 0.00000 0.00001

SumaTAT d41 0221 2 --------F---------.----------------.------------------ 0 0 2 0.00000 0.00000 0.00001

SumaTAT d41 0225 2 ------------------.----------------.-----------R------ 0 0 2 0.00000 0.00000 0.00001

SumaTAT d41 0226 2 -----------------F.----------------.------------------ 0 0 2 0.00000 0.00000 0.00001

SumaTAT d41 0228 2 --------G---------.----------------.------------------ 0 0 2 0.00000 0.00000 0.00001

SumaTAT d41 0230 2 ------------------.---H------------.------------------ 0 0 2 0.00000 0.00000 0.00001

SumaTAT d41 0232 2 ------------------.----------------.------------L----- 0 0 2 0.00000 0.00000 0.00001

SumaTAT d41 0116 1 ------------------.----------------.A---------------I- 0 0 1 0.00000 0.00000 0.00000

SumaTAT d41 0234 1 ------------------.---K--L---------.A-----K----------- 0 0 1 0.00000 0.00000 0.00000

SumaTAT d41 0239 1 ------P-----------.----------------.------------------ 0 0 1 0.00000 0.00000 0.00000

SumaTAT d41 0241 1 -----R------------.----------------.---M-------------- 0 0 1 0.00000 0.00000 0.00000

SumaTAT d41 0242 1 ------I-----H-----.----------------.------------------ 0 0 1 0.00000 0.00000 0.00000

SumaTAT d41 0243 1 ------------------.----------------.--------------I--- 0 0 1 0.00000 0.00000 0.00000

SumaTAT d41 0246 1 ------------------.-------K--------.------------------ 0 0 1 0.00000 0.00000 0.00000

SumaTAT d41 0247 1 ------------------.----------------.----------------Y- 2 0 1 0.00061 0.00000 0.00000

SumaTAT d41 0251 1 ------------------.--------------Y-.------------------ 0 0 1 0.00000 0.00000 0.00000

SumaTAT d41 0253 1 ------------------.----------------.-------S---------- 0 0 1 0.00000 0.00000 0.00000

SumaTAT d41 0254 1 -------------G----.------------D---.------------------ 0 0 1 0.00000 0.00000 0.00000

SumaTAT d41 0256 1 ------------------.Y---------------.----------------I- 0 0 1 0.00000 0.00000 0.00000

SumaTAT d41 0257 1 ------------------.----------------.---------T-------- 0 0 1 0.00000 0.00000 0.00000

SumaTAT d41 0258 1 ------------------.----------------.-T---------------- 0 0 1 0.00000 0.00000 0.00000

SumaTAT d41 0259 1 ------------D-----.----------------.------------------ 0 0 1 0.00000 0.00000 0.00000

SumaTAT d41 0260 1 -----R------------.----------------.----------T------- 0 0 1 0.00000 0.00000 0.00000

SumaTAT d41 0261 1 ------------------.----------------.----P------------- 0 0 1 0.00000 0.00000 0.00000

SumaTAT d41 0263 1 ------------F-----.----------------.------------------ 0 1 1 0.00000 0.00014 0.00000

SumaTAT d41 0265 1 ------------------.----------------.-----------------L 0 0 1 0.00000 0.00000 0.00000

SumaTAT d41 0266 1 ------------------.----------------.--------------IT-- 0 0 1 0.00000 0.00000 0.00000

SumaTAT d41 0267 1 -A----------------.----------------.------------------ 0 0 1 0.00000 0.00000 0.00000

SumaTAT d41 0269 1 ------------------.---P------------.------------------ 0 0 1 0.00000 0.00000 0.00000

SumaTAT d41 0271 1 -----R------------.----------------.-------L---------- 0 0 1 0.00000 0.00000 0.00000

SumaTAT d41 0273 1 ------------------.-------------T--.------------------ 0 0 1 0.00000 0.00000 0.00000

SumaTAT d41 0276 1 ------------------.----------------.-------Q--V------- 0 0 1 0.00000 0.00000 0.00000

SumaTAT d41 0277 1 ----------------W-.----------------.------------------ 0 0 1 0.00000 0.00000 0.00000

SumaTAT d41 0278 1 -----R------------.----------------.----------------H- 0 0 1 0.00000 0.00000 0.00000

SumaTAT d41 0280 1 ------------------.----------------.------K----------- 0 0 1 0.00000 0.00000 0.00000

SumaTAT d41 0281 1 -----------------S.----------------.------------------ 0 0 1 0.00000 0.00000 0.00000

SumaTAT d41 0282 1 ------------------.----------------.--M--------------- 0 0 1 0.00000 0.00000 0.00000

SumaTAT d41 0283 1 ------------------.----------------.R----------------- 0 0 1 0.00000 0.00000 0.00000

SumaTAT d41 0284 1 ------------------.----------------.---------------KI- 0 0 1 0.00000 0.00000 0.00000

SumaTAT d41 0287 1 ------------------.-Q--------------.------------------ 0 1 1 0.00000 0.00014 0.00000

SumaTAT d41 0288 1 ------------------.----------------.-------L---------- 0 0 1 0.00000 0.00000 0.00000

SumaTAT d41 0290 1 -------------G----.------Y---------.----L------------- 0 0 1 0.00000 0.00000 0.00000

SumaTAT d41 0291 1 ------------H-----.------------D---.------------------ 0 0 1 0.00000 0.00000 0.00000

SumaTAT d41 0292 1 ------------------.----------------.--------------Y--- 0 0 1 0.00000 0.00000 0.00000

SumaTAT d41 0294 1 ------------------.----------------.-----G----------S- 0 0 1 0.00000 0.00000 0.00000

SumaTAT d41 0296 1 ------------------.----------------.----Q-----S------- 0 0 1 0.00000 0.00000 0.00000

SumaTAT d41 0298 1 ------------------.-------IA-------.------------------ 0 0 1 0.00000 0.00000 0.00000

SumaTAT d41 0301 1 ------------------.----------------.-K-----------N---- 0 0 1 0.00000 0.00000 0.00000

SumaTAT d41 0303 1 ------------------.----------------.------P----------- 0 0 1 0.00000 0.00000 0.00000

SumaTAT d41 0307 1 ------------------.----------------.---------------P-- 0 0 1 0.00000 0.00000 0.00000

SumaTAT d41 0310 1 ------------------.----------------.D-----------R----- 0 0 1 0.00000 0.00000 0.00000

SumaTAT d41 0315 1 ------------------.-------L--------.------------------ 0 0 1 0.00000 0.00000 0.00000

SumaTAT d41 0316 1 ------------------.--------E-------.S--------------L-- 0 0 1 0.00000 0.00000 0.00000

SumaTAT d41 0317 1 ------------------.----------------.--------------N-I- 0 0 1 0.00000 0.00000 0.00000

SumaTAT d41 0319 1 ------------------.----A-----------.------------L----- 0 0 1 0.00000 0.00000 0.00000

SumaTAT d41 0321 1 ------------------.----------------.------------HN--I- 0 0 1 0.00000 0.00000 0.00000

SumaTAT d41 0325 1 ------------------.--------P-------.------------------ 0 0 1 0.00000 0.00000 0.00000

SumaTAT d41 0326 1 ------------------.----------------.---------------KY- 0 0 1 0.00000 0.00000 0.00000

SumaTAT d41 0327 1 ------------------.-R--------------.------------------ 0 0 1 0.00000 0.00000 0.00000

SumaTAT d41 0330 1 ------------------.--W-------------.------------------ 0 0 1 0.00000 0.00000 0.00000

SumaTAT d41 0335 1 --G---------------.----------------.------------------ 0 1 1 0.00000 0.00014 0.00000

SumaTAT d41 0336 1 ------------------.----------------.--------------C--- 0 2 1 0.00000 0.00028 0.00000

SumaTAT d41 0338 1 ------------------.------S---------.--------------T--- 0 0 1 0.00000 0.00000 0.00000

SumaTAT d41 0341 1 ------------------.----------------.---R--------R----- 0 0 1 0.00000 0.00000 0.00000

SumaTAT d41 0343 1 ------------------.----------------.------L----------- 0 0 1 0.00000 0.00000 0.00000

SumaTAT d41 0344 1 ------------------.----------------.--------P--------- 0 0 1 0.00000 0.00000 0.00000

SumaTAT d41 0346 1 ------------------.----------------.---T-------------- 0 0 1 0.00000 0.00000 0.00000

SumaTAT d41 0355 1 ------------------.----------C-----.------------------ 0 0 1 0.00000 0.00000 0.00000

SumaTAT d41 0358 1 ------------------.-------R--------.------------------ 0 0 1 0.00000 0.00000 0.00000

SumaTAT d41 0360 1 --------LP--------.----------------.------------------ 0 0 1 0.00000 0.00000 0.00000

SumaTAT d41 0362 1 ------------------.----------------.-------------A---- 0 0 1 0.00000 0.00000 0.00000

SumaTAT d41 0364 1 ------------------.----------------.----------V------Y 0 0 1 0.00000 0.00000 0.00000

SumaTAT d41 0366 1 ------------------.---------------H.-----------S------ 0 0 1 0.00000 0.00000 0.00000

SumaTAT d41 0368 1 ------------------.----------------.------------TS---- 0 0 1 0.00000 0.00000 0.00000

SumaTAT d41 0370 1 -----------F------.----------------.------------------ 0 0 1 0.00000 0.00000 0.00000

SumaTAT d41 0373 1 -----------S------.----------------.------------------ 0 0 1 0.00000 0.00000 0.00000

SumaTAT d41 0375 1 ------------------.----------------.-----K----------I- 0 0 1 0.00000 0.00000 0.00000

SumaTAT d41 0378 1 ------------------.-----------I----.------------------ 0 0 1 0.00000 0.00000 0.00000

SumaTAT d41 0379 1 ------------------.----------------.-----SS----------- 0 0 1 0.00000 0.00000 0.00000

SumaTAT d41 0380 1 -----------------R.----------------.------------------ 0 0 1 0.00000 0.00000 0.00000

SumaTAT d41 0382 1 -R----------------.----------------.---------------R-- 0 0 1 0.00000 0.00000 0.00000

SumaTAT d41 0386 1 ------------------.I-------K-------.------------------ 0 0 1 0.00000 0.00000 0.00000

SumaTAT d41 0389 1 ------------------.------------S---.----------------S- 0 0 1 0.00000 0.00000 0.00000

SumaTAT d41 0392 1 ------------------.----------------.--------Q-T------- 0 0 1 0.00000 0.00000 0.00000

SumaTAT d41 0393 1 ------------------.-----R----------.------------------ 0 0 1 0.00000 0.00000 0.00000

SumaTAT d41 0394 1 ----T-------------.----------------.------------------ 0 0 1 0.00000 0.00000 0.00000

SumaTAT d41 0399 1 ------------------.---------------F.------------------ 0 0 1 0.00000 0.00000 0.00000

SumaTAT d41 0401 1 ------------------.----------------.-----------------R 0 0 1 0.00000 0.00000 0.00000

SumaTAT d41 0403 1 ------------------.---------------N.------------------ 0 0 1 0.00000 0.00000 0.00000

SumaTAT d41 0408 1 -------G----------.----------------.------------------ 0 0 1 0.00000 0.00000 0.00000

SumaTAT d41 0409 1 ------------------.----------------.------K-----R----- 0 0 1 0.00000 0.00000 0.00000

SumaTAT d41 0411 1 ------------------.----------------.-----------------S 0 0 1 0.00000 0.00000 0.00000

SumaTAT d41 0415 1 ------------------.----------------.-------------A--I- 0 0 1 0.00000 0.00000 0.00000

SumaTAT d41 0418 1 ------------------.----------------.---M-------------- 0 0 1 0.00000 0.00000 0.00000

SumaTAT d41 0420 1 ------------------.----------------.-M----R----------- 0 0 1 0.00000 0.00000 0.00000

SumaTAT d41 0421 1 ------------------.------------S---.------L----------- 0 0 1 0.00000 0.00000 0.00000

SumaTAT d41 0422 1 ---R-----A--------.----------------.------------------ 0 0 1 0.00000 0.00000 0.00000

**_______________________________________________________________________________________________________________________________**

**SUMA Rev**

**SubReg day rank tally Sequence Tally Frequency**

**_______ ___ ____ _____ ______________________________________________________ _____________________ ________________________**

Transmitted SEGTRQARRNRRRRWR.QRQRQIQSL.SGWILSTHLGRPAEPVPLQLPPLERLT d05 d20 d41 d05 d20 d41

Bconsensus P---------------.E-----R-I.-E-----Y-------------------

Transmitted form:

SumaREV d05 0001 30928 ----------------.---------.--------------------------- 30927 7967 539 0.99198 0.97779 0.05210

SumaREV d20 0001 7967 ----------------.---------.--------------------------- 30927 7967 539 0.99198 0.97779 0.05210

SumaREV d41 0001 539 ----------------.---------.--------------------------- 30927 7967 539 0.99198 0.97779 0.05210

Most common form at d41, B consensus form, present but very rare at d05, d20:

Transmitted SEGTRQARRNRRRRWR.QRQRQIQSL.SGWILSTHLGRPAEPVPLQLPPLERLT

SumaREV d05 0084 1 ----------------.--------I.--------------------------- 1 1 4455 0.00003 0.00012 0.43064

SumaREV d20 0077 1 ----------------.--------I.--------------------------- 1 1 4455 0.00003 0.00012 0.43064

SumaREV d41 0001 4455 ----------------.--------I.--------------------------- 1 1 4455 0.00003 0.00012 0.43064

SumaREV d41 0015 44 ----------------.--------I.---------------------S----- 0 0 44 0.00000 0.00000 0.00425

SumaREV d41 0016 40 -K--------------.--------I.--------------------------- 0 0 40 0.00000 0.00000 0.00387

SumaREV d41 0017 35 ----------------.--------I.------------T-------------- 0 0 35 0.00000 0.00000 0.00338

SumaREV d41 0024 19 -----R----------.--------I.--------------------------- 0 0 19 0.00000 0.00000 0.00184

SumaREV d41 0026 15 ----------------.--------I.-E------------------------- 0 0 15 0.00000 0.00000 0.00145

SumaREV d41 0027 13 P---------------.--------I.--------------------------- 0 0 13 0.00000 0.00000 0.00126

SumaREV d41 0033 8 ----------------.--------I.----F---------------------- 0 0 8 0.00000 0.00000 0.00077

SumaREV d41 0035 8 ------------K---.--------I.--------------------------- 0 0 8 0.00000 0.00000 0.00077

SumaREV d41 0036 7 -----------K----.--------I.--------------------------- 0 0 7 0.00000 0.00000 0.00068

SumaREV d41 0041 5 ---------------T.--------I.--------------------------- 0 0 5 0.00000 0.00000 0.00048

SumaREV d41 0042 5 ----------------.--------I.-----------------F--------- 0 0 5 0.00000 0.00000 0.00048

SumaREV d41 0043 5 ----------------.---K----I.--------------------------- 0 0 5 0.00000 0.00000 0.00048

SumaREV d41 0044 5 ----------------.--------I.---------------------S----A 0 0 5 0.00000 0.00000 0.00048

SumaREV d41 0048 3 ----------------.--------I.------------------------K-- 0 0 3 0.00000 0.00000 0.00029

SumaREV d41 0049 3 -------Q--------.--------I.--------------------------- 0 0 3 0.00000 0.00000 0.00029

SumaREV d41 0050 3 -A--------------.--------I.--------------------------- 0 0 3 0.00000 0.00000 0.00029

SumaREV d41 0051 3 ----------------.--------I.----F-------V-------------- 0 0 3 0.00000 0.00000 0.00029

SumaREV d41 0052 3 ----------------.--------I.---M----------------------- 0 0 3 0.00000 0.00000 0.00029

SumaREV d41 0055 3 ----------------.-K------I.--------------------------- 0 0 3 0.00000 0.00000 0.00029

SumaREV d41 0056 3 ----------------.---I----I.--------------------------- 0 0 3 0.00000 0.00000 0.00029

SumaREV d41 0057 3 ----------------.--------I.----------------S---------- 0 0 3 0.00000 0.00000 0.00029

SumaREV d41 0058 3 ---N------------.--------I.--------------------------- 0 0 3 0.00000 0.00000 0.00029

SumaREV d41 0059 3 A---------------.--------I.--------------------------- 0 0 3 0.00000 0.00000 0.00029

SumaREV d41 0061 3 ----------------.--------I.---------E----------------- 0 0 3 0.00000 0.00000 0.00029

SumaREV d41 0064 3 ----------------.--------I.-----------T--------------- 0 0 3 0.00000 0.00000 0.00029

SumaREV d41 0066 2 ----------------.--R-----I.--------------------------- 0 0 2 0.00000 0.00000 0.00019

SumaREV d41 0067 2 ----------------.----K---I.--------------------------- 0 0 2 0.00000 0.00000 0.00019

SumaREV d41 0068 2 ----------Q-----.--------I.--------------------------- 0 0 2 0.00000 0.00000 0.00019

SumaREV d41 0072 2 --------------R-.--------I.--------------------------- 0 0 2 0.00000 0.00000 0.00019

SumaREV d41 0073 2 ----------------.--------I.--------------------------A 0 0 2 0.00000 0.00000 0.00019

SumaREV d41 0075 2 ----------------.--------I.-------------------------P- 0 0 2 0.00000 0.00000 0.00019

SumaREV d41 0076 2 ----------------.--------I.-----I--------------------- 0 0 2 0.00000 0.00000 0.00019

SumaREV d41 0077 2 -D--------------.--------I.--------------------------- 0 0 2 0.00000 0.00000 0.00019

SumaREV d41 0078 2 ----G-----------.--------I.--------------------------- 0 0 2 0.00000 0.00000 0.00019

SumaREV d41 0081 2 ----------------.---S----I.--------------------------- 0 0 2 0.00000 0.00000 0.00019

SumaREV d41 0084 2 ----------------.--------I.------I--------------S----- 0 0 2 0.00000 0.00000 0.00019

SumaREV d41 0089 2 ----------------.--------I.-----------------R--------- 0 0 2 0.00000 0.00000 0.00019

SumaREV d41 0091 2 ----------------.---G----I.--------------------------- 0 0 2 0.00000 0.00000 0.00019

SumaREV d41 0093 2 ----------------.--------I.-----------S--------------- 0 0 2 0.00000 0.00000 0.00019

SumaREV d41 0094 2 ----------------.--------I.----P---------------------- 0 0 2 0.00000 0.00000 0.00019

SumaREV d41 0098 2 ----------------.---T----I.--------------------------- 0 0 2 0.00000 0.00000 0.00019

SumaREV d41 0115 1 ----------------.--------I.-----G--------------------- 0 0 1 0.00000 0.00000 0.00010

SumaREV d41 0116 1 ----------------.--------I.-------------K------------- 0 0 1 0.00000 0.00000 0.00010

SumaREV d41 0118 1 ---P------------.--------I.--------------------------- 0 0 1 0.00000 0.00000 0.00010

SumaREV d41 0125 1 ----------------.--------I.--------------H------------ 0 0 1 0.00000 0.00000 0.00010

SumaREV d41 0130 1 ----------------.--------I.---------------M----------- 0 0 1 0.00000 0.00000 0.00010

SumaREV d41 0136 1 ----------------.--------I.-----------------P--------- 0 0 1 0.00000 0.00000 0.00010

SumaREV d41 0148 1 -----------G----.--------I.--------------------------- 0 0 1 0.00000 0.00000 0.00010

SumaREV d41 0151 1 ---------------G.--------I.--------------------------- 0 0 1 0.00000 0.00000 0.00010

SumaREV d41 0157 1 ----------------.--------I.-----------------------D--- 0 0 1 0.00000 0.00000 0.00010

SumaREV d41 0163 1 ----Q-----------.--------I.--------------------------- 0 0 1 0.00000 0.00000 0.00010

SumaREV d41 0164 1 ----------------.--------I.---V----------------------- 0 0 1 0.00000 0.00000 0.00010

SumaREV d41 0165 1 -----------K----.--------I.---------------------S----- 0 0 1 0.00000 0.00000 0.00010

SumaREV d41 0166 1 ------------T---.--------I.--------------------------- 0 0 1 0.00000 0.00000 0.00010

SumaREV d41 0169 1 ---I------------.--------I.--------------------------- 0 0 1 0.00000 0.00000 0.00010

SumaREV d41 0171 1 ----------------.--------I.------------V-------------- 0 0 1 0.00000 0.00000 0.00010

SumaREV d41 0183 1 -G--------------.--------I.--------------------------- 0 0 1 0.00000 0.00000 0.00010

SumaREV d41 0184 1 -------L--------.--------I.--------------------------- 0 0 1 0.00000 0.00000 0.00010

SumaREV d41 0189 1 ----------------.--------I.----------------------P---- 0 0 1 0.00000 0.00000 0.00010

SumaREV d41 0196 1 ----------------.-----V--I.--------------------------- 0 0 1 0.00000 0.00000 0.00010

SumaREV d41 0197 1 ----------------.--------I.---------------A----------- 0 0 1 0.00000 0.00000 0.00010

SumaREV d41 0198 1 ---------------T.--------I.----------------S---------- 0 0 1 0.00000 0.00000 0.00010

SumaREV d41 0201 1 ----------------.--------I.--------------T------------ 0 0 1 0.00000 0.00000 0.00010

SumaREV d41 0204 1 ----------------.--------I.------------------R-------- 0 0 1 0.00000 0.00000 0.00010

2nd most common escape at d41 (Q/R), B consensus form, present d05, d20, though rare:

SumaREV d41 0029 10 ----------------.------R-I.--------------------------- 0 0 10 0.00000 0.00000 0.00097

SumaREV d41 0156 1 ----------------.------R-I.-------Q------------------- 0 0 1 0.00000 0.00000 0.00010

SumaREV d41 0200 1 ----------------.------R-I.---------------------S----- 0 0 1 0.00000 0.00000 0.00010

SumaREV d05 0044 2 ----------------.------R--.--------------------------- 2 3 2964 0.00006 0.00037 0.28652

SumaREV d20 0021 3 ----------------.------R--.--------------------------- 2 3 2964 0.00006 0.00037 0.28652

SumaREV d41 0002 2964 ----------------.------R--.--------------------------- 2 3 2964 0.00006 0.00037 0.28652

SumaREV d41 0020 26 P---------------.------R--.--------------------------- 0 0 26 0.00000 0.00000 0.00251

SumaREV d41 0028 11 ----------------.-----TR--.--------------------------- 0 0 11 0.00000 0.00000 0.00106

SumaREV d41 0030 10 -----------K----.------R--.--------------------------- 0 0 10 0.00000 0.00000 0.00097

SumaREV d41 0040 5 ----------------.------R--.---------------------S----- 0 0 5 0.00000 0.00000 0.00048

SumaREV d41 0045 4 ----------------.------R--.-------Q------------------- 0 0 4 0.00000 0.00000 0.00039

SumaREV d41 0046 4 -K--------------.------R--.--------------------------- 0 0 4 0.00000 0.00000 0.00039

SumaREV d41 0047 4 ----------------.------R--.------------------------K-- 0 0 4 0.00000 0.00000 0.00039

SumaREV d41 0063 3 ----------------.------R--.--------------T------------ 0 0 3 0.00000 0.00000 0.00029

SumaREV d41 0083 2 ----------------.------R--.---------------------L----- 0 0 2 0.00000 0.00000 0.00019

SumaREV d41 0092 2 ------V---------.------R--.--------------------------- 0 0 2 0.00000 0.00000 0.00019

SumaREV d41 0095 2 ----------------.----K-R--.--------------------------- 0 0 2 0.00000 0.00000 0.00019

SumaREV d41 0099 2 ----------------.------R--.-----R--------------------- 0 0 2 0.00000 0.00000 0.00019

SumaREV d41 0101 1 ----------------.------R--.-----G--------------------- 0 0 1 0.00000 0.00000 0.00010

SumaREV d41 0108 1 ----------------.------R--.------------V-------------- 0 0 1 0.00000 0.00000 0.00010

SumaREV d41 0117 1 -------------G--.------R--.--------------------------- 0 0 1 0.00000 0.00000 0.00010

SumaREV d41 0119 1 P----L----------.------R--.--------------------------- 0 0 1 0.00000 0.00000 0.00010

SumaREV d41 0126 1 ----------------.------R--.---------R----------------- 0 0 1 0.00000 0.00000 0.00010

SumaREV d41 0134 1 ----------------.-K----R--.--------------------------- 0 0 1 0.00000 0.00000 0.00010

SumaREV d41 0139 1 ----Q-----------.------R--.--------------------------- 0 0 1 0.00000 0.00000 0.00010

SumaREV d41 0140 1 ----------------.------R-F.--------------------------- 0 0 1 0.00000 0.00000 0.00010

SumaREV d41 0145 1 ----------------.------R--.------------T-------------- 0 0 1 0.00000 0.00000 0.00010

SumaREV d41 0149 1 ----------------.------R--.--------------------------N 0 0 1 0.00000 0.00000 0.00010

SumaREV d41 0150 1 ------------K---.------R--.--------------------------- 0 0 1 0.00000 0.00000 0.00010

SumaREV d41 0153 1 -D--------------.------R--.--------------------------- 0 0 1 0.00000 0.00000 0.00010

SumaREV d41 0162 1 --R-------------.------R--.--------------------------- 0 0 1 0.00000 0.00000 0.00010

SumaREV d41 0168 1 ----------------.------R--.---------------M----------- 0 0 1 0.00000 0.00000 0.00010

SumaREV d41 0172 1 ----------------.------R--.----------------S---------- 0 0 1 0.00000 0.00000 0.00010

SumaREV d41 0174 1 P--------------G.------R--.--------------------------- 0 0 1 0.00000 0.00000 0.00010

SumaREV d41 0177 1 ----------------.------R--.-----------S--------------- 0 0 1 0.00000 0.00000 0.00010

SumaREV d41 0178 1 --------K-------.------R--.--------------------------- 0 0 1 0.00000 0.00000 0.00010

SumaREV d41 0182 1 ----------------.------R-V.--------------------------- 0 0 1 0.00000 0.00000 0.00010

SumaREV d41 0185 1 ----------------.------R--.------A-------------------- 0 0 1 0.00000 0.00000 0.00010

SumaREV d41 0186 1 ----------------.------R--.--------------------Q------ 0 0 1 0.00000 0.00000 0.00010

SumaREV d41 0190 1 ----------------.------R--.----F---------------------- 0 0 1 0.00000 0.00000 0.00010

SumaREV d41 0191 1 ----------------.---K--R--.--------------------------- 0 0 1 0.00000 0.00000 0.00010

SumaREV d41 0192 1 ----------------.------R--.------------------R-------- 0 0 1 0.00000 0.00000 0.00010

SumaREV d41 0202 1 ----------------.---T--R--.--------------------------- 0 0 1 0.00000 0.00000 0.00010

3rd most common escape at d41 (Q/K):

SumaREV d41 0060 3 ----------------.------K-I.--------------------------- 0 0 3 0.00000 0.00000 0.00029

SumaREV d41 0144 1 ------------T---.------K-I.--------------------------- 0 0 1 0.00000 0.00000 0.00010

SumaREV d41 0004 449 ----------------.------K--.--------------------------- 0 0 449 0.00000 0.00000 0.04340

SumaREV d41 0037 7 ----------------.------K--.---------------A----------- 0 0 7 0.00000 0.00000 0.00068

SumaREV d41 0100 2 -D--------------.------K--.--------------------------- 0 0 2 0.00000 0.00000 0.00019

SumaREV d41 0107 1 ------P---------.------K--.--------------------------- 0 0 1 0.00000 0.00000 0.00010

SumaREV d41 0111 1 P---------------.------K--.--------------------------- 0 0 1 0.00000 0.00000 0.00010

SumaREV d41 0121 1 ----------------.------K--.------------T-------------- 0 0 1 0.00000 0.00000 0.00010

SumaREV d41 0159 1 ----------------.------K--.----------------S---------- 0 0 1 0.00000 0.00000 0.00010

SumaREV d41 0180 1 ----------------.------K--.----------------T---------- 0 0 1 0.00000 0.00000 0.00010

SumaREV d41 0195 1 ----------------.-K----K--.--------------------------- 0 0 1 0.00000 0.00000 0.00010

SumaREV d41 0205 1 ----------------.------K--.-------------------P------- 0 0 1 0.00000 0.00000 0.00010

SumaREV d41 0206 1 ----------------.---S--K--.--------------------------- 0 0 1 0.00000 0.00000 0.00010

4th most common escape at d41 (Q/R):

SumaREV d41 0071 2 ----------------.----R---I.--------------------------- 0 0 2 0.00000 0.00000 0.00019

SumaREV d05 0071 1 ----------------.----R----.--------------------------- 1 0 194 0.00003 0.00000 0.01875

SumaREV d41 0005 194 ----------------.----R----.--------------------------- 1 0 194 0.00003 0.00000 0.01875

SumaREV d41 0054 3 ----------------.----R----.---------------------S----- 0 0 3 0.00000 0.00000 0.00029

SumaREV d41 0069 2 ----------------.----R----.-----------S--------------- 0 0 2 0.00000 0.00000 0.00019

SumaREV d41 0154 1 ----------------.----R----.-----------L--------------- 0 0 1 0.00000 0.00000 0.00010

SumaREV d41 0181 1 ----------------.----R--L-.--------------------------- 0 0 1 0.00000 0.00000 0.00010

5th most common form at d41 (R/K) may be a processing escape, as it occurs 1 amino acid before the epitope:

SumaREV d41 0022 20 ---------------K.--------I.--------------------------- 0 0 20 0.00000 0.00000 0.00193

SumaREV d41 0129 1 ---------------K.------K-I.--------------------------- 0 0 1 0.00000 0.00000 0.00010

SumaREV d41 0031 10 ---------------K.------R--.--------------------------- 0 0 10 0.00000 0.00000 0.00097

SumaREV d05 0008 7 ---------------K.---------.--------------------------- 7 0 193 0.00022 0.00000 0.01866

SumaREV d41 0103 1 ---------------K.------K--.------------V-------------- 0 0 1 0.00000 0.00000 0.00010

SumaREV d41 0208 1 ---------------K.------K--.--------------------------- 0 0 1 0.00000 0.00000 0.00010

SumaREV d41 0006 193 ---------------K.---------.--------------------------- 7 0 193 0.00022 0.00000 0.01866

SumaREV d41 0113 1 ---------------K.-------L-.--------------------------- 0 0 1 0.00000 0.00000 0.00010

SumaREV d41 0147 1 -K-------------K.---------.--------------------------- 0 0 1 0.00000 0.00000 0.00010

SumaREV d41 0160 1 -----------K---K.---------.--------------------------- 0 0 1 0.00000 0.00000 0.00010

SumaREV d41 0170 1 -G-------------K.---------.--------------------------- 0 0 1 0.00000 0.00000 0.00010

All additional forms found repeated more than once at day 41, -------L- is potentially interesting as it is found at a low level at each time point:

SumaREV d41 0007 178 ----------------.------L--.--------------------------- 2 0 178 0.00006 0.00000 0.01721

SumaREV d41 0008 172 ----------------.---I-----.--------------------------- 0 1 172 0.00000 0.00012 0.01663

SumaREV d41 0009 170 ----------------.-K-------.--------------------------- 4 0 170 0.00013 0.00000 0.01643

SumaREV d41 0010 86 ----------------.--R------.--------------------------- 0 0 86 0.00000 0.00000 0.00831

SumaREV d41 0011 70 ----------------.--------V.--------------------------- 0 1 70 0.00000 0.00012 0.00677

SumaREV d41 0012 67 ----------------.----K----.--------------------------- 0 0 67 0.00000 0.00000 0.00648

SumaREV d41 0013 54 ----------------.--H------.--------------------------- 1 1 54 0.00003 0.00012 0.00522

SumaREV d41 0014 50 ----------------.---S-----.--------------------------- 5 0 50 0.00016 0.00000 0.00483

SumaREV d41 0018 33 ----------------.---T-----.--------------------------- 2 0 33 0.00006 0.00000 0.00319

SumaREV d41 0019 31 ----------------.---G-----.--------------------------- 0 0 31 0.00000 0.00000 0.00300

SumaREV d41 0021 22 ----------------.-------L-.--------------------------- 27 4 22 0.00087 0.00049 0.00213

SumaREV d41 0023 19 ----------------.---------.---------------------S----- 3 1 19 0.00010 0.00012 0.00184

SumaREV d41 0025 17 ----------------.--E------.--------------------------- 0 0 17 0.00000 0.00000 0.00164

SumaREV d41 0032 9 ----------------.K--------.--------------------------- 0 0 9 0.00000 0.00000 0.00087

SumaREV d41 0034 8 ----------------.---K-----.--------------------------- 3 1 8 0.00010 0.00012 0.00077

SumaREV d41 0038 7 ----------------.--H------.-R------------------------- 0 0 7 0.00000 0.00000 0.00068

SumaREV d41 0039 6 ----------------.-------T-.--------------------------- 3 0 6 0.00010 0.00000 0.00058

SumaREV d41 0053 3 ----------------.----E----.--------------------------- 0 0 3 0.00000 0.00000 0.00029

SumaREV d41 0062 3 ----------------.--H------.---------------------S----- 0 0 3 0.00000 0.00000 0.00029

SumaREV d41 0065 3 ----------------.---------.-E------------------------- 6 2 3 0.00019 0.00025 0.00029

SumaREV d41 0070 2 -K--------------.---I-----.--------------------------- 0 0 2 0.00000 0.00000 0.00019

SumaREV d41 0074 2 ----------------.----H----.--------------------------- 0 1 2 0.00000 0.00012 0.00019

SumaREV d41 0079 2 ----------------.---------.-----------------F--------- 10 19 2 0.00032 0.00233 0.00019

SumaREV d41 0080 2 ----------------.-------L-.--------------S------------ 0 0 2 0.00000 0.00000 0.00019

SumaREV d41 0082 2 ----------------.-------A-.--------------------------- 0 2 2 0.00000 0.00025 0.00019

SumaREV d41 0086 2 ----------------.-I-------.--------------------------- 0 0 2 0.00000 0.00000 0.00019

SumaREV d41 0087 2 ----------------.-----L---.--------------------------- 0 0 2 0.00000 0.00000 0.00019

SumaREV d41 0088 2 ----------------.R--------.--------------------------- 0 0 2 0.00000 0.00000 0.00019

SumaREV d41 0096 2 ----------------.--K------.--------------------------- 0 0 2 0.00000 0.00000 0.00019

Other day 05 sequences:

SumaREV d05 0002 27 ----------------.-------L-.--------------------------- 27 4 22 0.00087 0.00049 0.00213

SumaREV d05 0003 10 ----------------.---------.-----------------F--------- 10 19 2 0.00032 0.00233 0.00019

SumaREV d05 0004 9 ----------Q-----.---------.--------------------------- 9 5 0 0.00029 0.00061 0.00000

SumaREV d05 0005 9 -----------K----.---------.--------------------------- 9 2 0 0.00029 0.00025 0.00000

SumaREV d05 0006 8 ----------------.---------.----------------S---------- 8 3 1 0.00026 0.00037 0.00010

SumaREV d05 0007 8 ----------------.---------.-------------K------------- 8 5 0 0.00026 0.00061 0.00000

SumaREV d05 0009 7 ----------------.---------.------------------------K-- 7 7 0 0.00022 0.00086 0.00000

SumaREV d05 0010 7 --------K-------.---------.--------------------------- 7 8 0 0.00022 0.00098 0.00000

SumaREV d05 0011 6 ----------------.---------.-----N--------------------- 6 2 0 0.00019 0.00025 0.00000

SumaREV d05 0012 6 ----------------.---------.-E------------------------- 6 2 3 0.00019 0.00025 0.00029

SumaREV d05 0013 5 ----------------.---S-----.--------------------------- 5 0 50 0.00016 0.00000 0.00483

SumaREV d05 0014 5 -K--------------.---------.--------------------------- 5 4 0 0.00016 0.00049 0.00000

SumaREV d05 0016 4 -------Q--------.---------.--------------------------- 4 4 0 0.00013 0.00049 0.00000

SumaREV d05 0017 4 ---I------------.---------.--------------------------- 4 0 0 0.00013 0.00000 0.00000

SumaREV d05 0018 4 ----------------.--------F.--------------------------- 4 1 0 0.00013 0.00012 0.00000

SumaREV d05 0019 4 ----------------.-K-------.--------------------------- 4 0 170 0.00013 0.00000 0.01643

SumaREV d05 0020 4 ----Q-----------.---------.--------------------------- 4 4 0 0.00013 0.00049 0.00000

SumaREV d05 0021 3 ----------------.---------.---------E----------------- 3 3 0 0.00010 0.00037 0.00000

SumaREV d05 0022 3 ----------------.---------.------------------R-------- 3 1 0 0.00010 0.00012 0.00000

SumaREV d05 0023 3 ----------------.---------.---------------------S----- 3 1 19 0.00010 0.00012 0.00184

SumaREV d05 0024 3 ----------------.---------.------I-------------------- 3 1 0 0.00010 0.00012 0.00000

SumaREV d05 0025 3 ----------------.-------T-.--------------------------- 3 0 6 0.00010 0.00000 0.00058

SumaREV d05 0026 3 ------------K---.---------.--------------------------- 3 3 1 0.00010 0.00037 0.00010

SumaREV d05 0028 3 P---------------.---------.--------------------------- 3 0 0 0.00010 0.00000 0.00000

SumaREV d05 0029 3 ----------------.---K-----.--------------------------- 3 1 8 0.00010 0.00012 0.00077

SumaREV d05 0030 3 ----------------.---------.----------------------P---- 3 0 0 0.00010 0.00000 0.00000

SumaREV d05 0031 3 ----------------.---------.----------G---------------- 3 0 0 0.00010 0.00000 0.00000

SumaREV d05 0032 3 ----------------.---------.--------------S------------ 3 3 0 0.00010 0.00037 0.00000

SumaREV d05 0033 2 ---------------I.---------.-----------------F--------- 2 0 0 0.00006 0.00000 0.00000

SumaREV d05 0035 2 ----------------.---------.--------P------------------ 2 0 0 0.00006 0.00000 0.00000

SumaREV d05 0036 2 ----------------.---------.---------------------Q----- 2 0 0 0.00006 0.00000 0.00000

SumaREV d05 0037 2 ---N------------.---------.--------------------------- 2 1 0 0.00006 0.00012 0.00000

SumaREV d05 0038 2 -------------G--.---------.--------------------------- 2 4 0 0.00006 0.00049 0.00000

SumaREV d05 0040 2 ----------------.---------.------------V-------------- 2 2 1 0.00006 0.00025 0.00010

SumaREV d05 0042 2 ----------------.---T-----.--------------------------- 2 0 33 0.00006 0.00000 0.00319

SumaREV d05 0043 2 ----------------.------L--.--------------------------- 2 0 178 0.00006 0.00000 0.01721

SumaREV d05 0045 2 ----------------.---------.------------E-------------- 2 0 0 0.00006 0.00000 0.00000

SumaREV d05 0046 2 ----------------.---------.-------------------------F- 2 1 0 0.00006 0.00012 0.00000

SumaREV d05 0047 2 ----------------.---------.----------K---------------- 2 2 0 0.00006 0.00025 0.00000

SumaREV d05 0048 2 ----------------.---------.-R------------------------- 2 1 0 0.00006 0.00012 0.00000

SumaREV d05 0049 2 ---------D------.---------.--------------------------- 2 0 0 0.00006 0.00000 0.00000

SumaREV d05 0050 1 -----E----------.---------.--------------------------- 1 0 0 0.00003 0.00000 0.00000

SumaREV d05 0051 1 ----------------.--H------.--------------------------- 1 1 54 0.00003 0.00012 0.00522

SumaREV d05 0052 1 -G--------------.---------.--------------------------- 1 0 0 0.00003 0.00000 0.00000

SumaREV d05 0053 1 ----------------.E--------.--------------------------- 1 0 0 0.00003 0.00000 0.00000

SumaREV d05 0054 1 ----------------.---------.--------------T------------ 1 0 0 0.00003 0.00000 0.00000

SumaREV d05 0055 1 --------------C-.---------.--------------------------- 1 0 0 0.00003 0.00000 0.00000

SumaREV d05 0056 1 ----------------.---------.------------------------S-- 1 0 0 0.00003 0.00000 0.00000

SumaREV d05 0057 1 -------------K--.---------.--------------------------- 1 4 0 0.00003 0.00049 0.00000

SumaREV d05 0058 1 ---S------------.---------.--------------------------- 1 0 0 0.00003 0.00000 0.00000

SumaREV d05 0060 1 ----L-----------.---------.--------------------------- 1 0 0 0.00003 0.00000 0.00000

SumaREV d05 0061 1 ----------------.---------.---------------------L----- 1 0 0 0.00003 0.00000 0.00000

SumaREV d05 0062 1 ----------------.---------.----------------T---------- 1 1 0 0.00003 0.00012 0.00000

SumaREV d05 0063 1 ------T---------.---------.--------------------------- 1 0 0 0.00003 0.00000 0.00000

SumaREV d05 0064 1 ----------------.---------.----I---------------------- 1 0 0 0.00003 0.00000 0.00000

SumaREV d05 0065 1 ----------------.---------.-----------T--------------- 1 0 0 0.00003 0.00000 0.00000

SumaREV d05 0066 1 ----------------.---------.-------N------------------- 1 0 0 0.00003 0.00000 0.00000

SumaREV d05 0067 1 ----------------.---------.N-------------------------- 1 0 0 0.00003 0.00000 0.00000

SumaREV d05 0068 1 -D--------------.---------.--------------------------- 1 3 0 0.00003 0.00037 0.00000

SumaREV d05 0069 1 -------L--------.---------.--------------------------- 1 1 0 0.00003 0.00012 0.00000

SumaREV d05 0070 1 ----------------.---------.-----------S--------------- 1 1 0 0.00003 0.00012 0.00000

SumaREV d05 0072 1 Y---------------.---------.--------------------------- 1 0 0 0.00003 0.00000 0.00000

SumaREV d05 0073 1 ---------------G.---------.--------------------------- 1 1 0 0.00003 0.00012 0.00000

SumaREV d05 0074 1 ----------------.---------.-----I--------------------- 1 0 0 0.00003 0.00000 0.00000

SumaREV d05 0075 1 ----------------.---------.--------------------------A 1 2 0 0.00003 0.00025 0.00000

SumaREV d05 0076 1 ----------------.---------.----------------------F---- 1 0 0 0.00003 0.00000 0.00000

SumaREV d05 0078 1 --R-------------.---------.--------------------------- 1 1 1 0.00003 0.00012 0.00010

SumaREV d05 0079 1 ----------------.---------.T-------------------------- 1 0 0 0.00003 0.00000 0.00000

SumaREV d05 0080 1 ----------------.---------.-----------------------G--- 1 0 0 0.00003 0.00000 0.00000

SumaREV d05 0082 1 ----------------.---------.---------R----------------- 1 3 0 0.00003 0.00037 0.00000

SumaREV d05 0083 1 -------------S--.---------.--------------------------- 1 0 0 0.00003 0.00000 0.00000

SumaREV d05 0085 1 ----------------.---------.----------------H---------- 1 0 0 0.00003 0.00000 0.00000

SumaREV d05 0086 1 ----------------.---------.----------------------R---- 1 0 0 0.00003 0.00000 0.00000

SumaREV d05 0087 1 F---------------.---------.--------------------------- 1 3 1 0.00003 0.00037 0.00010

SumaREV d05 0088 1 ----------------.---------.-----G--------------------- 1 2 0 0.00003 0.00025 0.00000

SumaREV d05 0089 1 --E-------------.---------.--------------------------- 1 0 0 0.00003 0.00000 0.00000

SumaREV d05 0090 1 ----------------.---------.------------T-------------- 1 4 0 0.00003 0.00049 0.00000

SumaREV d05 0091 1 ----------------.---------.-V------------------------- 1 0 1 0.00003 0.00000 0.00010

Other day 20 sequences:

SumaREV d20 0002 19 ----------------.---------.-----------------F--------- 10 19 2 0.00032 0.00233 0.00019

SumaREV d20 0003 8 --------K-------.---------.--------------------------- 7 8 0 0.00022 0.00098 0.00000

SumaREV d20 0004 7 ----------------.---------.------------------------K-- 7 7 0 0.00022 0.00086 0.00000

SumaREV d20 0006 5 ----------Q-----.---------.--------------------------- 9 5 0 0.00029 0.00061 0.00000

SumaREV d20 0007 5 ----------------.---------.-------------K------------- 8 5 0 0.00026 0.00061 0.00000

SumaREV d20 0008 4 -------Q--------.---------.--------------------------- 4 4 0 0.00013 0.00049 0.00000

SumaREV d20 0009 4 -------------K--.---------.--------------------------- 1 4 0 0.00003 0.00049 0.00000

SumaREV d20 0010 4 -K--------------.---------.--------------------------- 5 4 0 0.00016 0.00049 0.00000

SumaREV d20 0011 4 -------------G--.---------.--------------------------- 2 4 0 0.00006 0.00049 0.00000

SumaREV d20 0012 4 ----------------.---------.---------------------T----- 0 4 0 0.00000 0.00049 0.00000

SumaREV d20 0013 4 ----------------.-------L-.--------------------------- 27 4 22 0.00087 0.00049 0.00213

SumaREV d20 0014 4 ----Q-----------.---------.--------------------------- 4 4 0 0.00013 0.00049 0.00000

SumaREV d20 0015 4 ----------------.---------.------------T-------------- 1 4 0 0.00003 0.00049 0.00000

SumaREV d20 0016 3 ----------------.---------.---------E----------------- 3 3 0 0.00010 0.00037 0.00000

SumaREV d20 0017 3 -----R----------.---------.--------------------------- 0 3 0 0.00000 0.00037 0.00000

SumaREV d20 0018 3 -D--------------.---------.--------------------------- 1 3 0 0.00003 0.00037 0.00000

SumaREV d20 0019 3 ----------------.---------.----------------S---------- 8 3 1 0.00026 0.00037 0.00010

SumaREV d20 0020 3 ------------K---.---------.--------------------------- 3 3 1 0.00010 0.00037 0.00010

SumaREV d20 0022 3 ----------------.---------.---------R----------------- 1 3 0 0.00003 0.00037 0.00000

SumaREV d20 0023 3 F---------------.---------.--------------------------- 1 3 1 0.00003 0.00037 0.00010

SumaREV d20 0024 3 ----------------.---------.--------------S------------ 3 3 0 0.00010 0.00037 0.00000

SumaREV d20 0026 2 ----------------.-------A-.--------------------------- 0 2 2 0.00000 0.00025 0.00019

SumaREV d20 0027 2 ----------------.---------.---------------A----------- 0 2 1 0.00000 0.00025 0.00010

SumaREV d20 0029 2 -V--------------.---------.--------------------------- 0 2 0 0.00000 0.00025 0.00000

SumaREV d20 0030 2 ----------------.H--------.--------------------------- 0 2 0 0.00000 0.00025 0.00000

SumaREV d20 0031 2 ----------------.---------.------------V-------------- 2 2 1 0.00006 0.00025 0.00010

SumaREV d20 0032 2 ----------------.---------.-----N--------------------- 6 2 0 0.00019 0.00025 0.00000

SumaREV d20 0033 2 ----------------.---------.--------------------------A 1 2 0 0.00003 0.00025 0.00000

SumaREV d20 0034 2 -----------K----.---------.--------------------------- 9 2 0 0.00029 0.00025 0.00000

SumaREV d20 0035 2 ----------------.---------.----F---------------------- 0 2 0 0.00000 0.00025 0.00000

SumaREV d20 0036 2 ----------------.---------.--L------------------------ 0 2 0 0.00000 0.00025 0.00000

SumaREV d20 0037 2 ----------------.---------.----------K---------------- 2 2 0 0.00006 0.00025 0.00000

SumaREV d20 0038 2 ----------------.---------.-----G--------------------- 1 2 0 0.00003 0.00025 0.00000

SumaREV d20 0039 2 ----------------.---------.-E------------------------- 6 2 3 0.00019 0.00025 0.00029

SumaREV d20 0040 1 ----------------.---------.-----R------------------K-- 0 1 0 0.00000 0.00012 0.00000

SumaREV d20 0041 1 ----------------.---I-----.--------------------------- 0 1 172 0.00000 0.00012 0.01663

SumaREV d20 0042 1 ----------------.---------.-------------------P------- 0 1 0 0.00000 0.00012 0.00000

SumaREV d20 0043 1 ----------------.--H------.--------------------------- 1 1 54 0.00003 0.00012 0.00522

SumaREV d20 0044 1 ----------------.---------.------------------R-------- 3 1 0 0.00010 0.00012 0.00000

SumaREV d20 0046 1 ----------------.---------.--------------L------------ 0 1 0 0.00000 0.00012 0.00000

SumaREV d20 0047 1 ----------------.---------.---------------------S----- 3 1 19 0.00010 0.00012 0.00184

SumaREV d20 0049 1 ----------------.---------.------I-------------------- 3 1 0 0.00010 0.00012 0.00000

SumaREV d20 0050 1 ----------------.---------.G-------------------------- 0 1 0 0.00000 0.00012 0.00000

SumaREV d20 0051 1 ----------------.---------.--------M------------------ 0 1 0 0.00000 0.00012 0.00000

SumaREV d20 0052 1 ---N------------.---------.--------------------------- 2 1 0 0.00006 0.00012 0.00000

SumaREV d20 0053 1 -----H----------.---------.--------------------------- 0 1 0 0.00000 0.00012 0.00000

SumaREV d20 0054 1 -K--------------.---------.-----------------F--------- 0 1 0 0.00000 0.00012 0.00000

SumaREV d20 0055 1 ----------------.---------.-----------------------V--- 0 1 0 0.00000 0.00012 0.00000

SumaREV d20 0056 1 ----------------.---------.----------------T---------- 1 1 0 0.00003 0.00012 0.00000

SumaREV d20 0057 1 ----------------.---------.-------------V------------- 0 1 0 0.00000 0.00012 0.00000

SumaREV d20 0058 1 ----------------.---------.--------------------------I 0 1 0 0.00000 0.00012 0.00000

SumaREV d20 0059 1 ----------------.---------.--------------------------N 0 1 0 0.00000 0.00012 0.00000

SumaREV d20 0060 1 ----------------.---------.----------------L---------- 0 1 0 0.00000 0.00012 0.00000

SumaREV d20 0061 1 ----------------.---------.---T----------------------- 0 1 0 0.00000 0.00012 0.00000

SumaREV d20 0063 1 -------L--------.---------.--------------------------- 1 1 0 0.00003 0.00012 0.00000

SumaREV d20 0064 1 ----------------.---------.-----------S--------------- 1 1 0 0.00003 0.00012 0.00000

SumaREV d20 0065 1 ----------------.--------F.--------------------------- 4 1 0 0.00013 0.00012 0.00000

SumaREV d20 0066 1 ----------------.---------.-----------------P--------- 0 1 0 0.00000 0.00012 0.00000

SumaREV d20 0067 1 ----------------.---------.-----------L--------------- 0 1 0 0.00000 0.00012 0.00000

SumaREV d20 0068 1 ----------------.---------.-------Y------------------- 0 1 0 0.00000 0.00012 0.00000

SumaREV d20 0069 1 ----------------.----H----.--------------------------- 0 1 2 0.00000 0.00012 0.00019

SumaREV d20 0070 1 ---------------G.---------.--------------------------- 1 1 0 0.00003 0.00012 0.00000

SumaREV d20 0072 1 --R-------------.---------.--------------------------- 1 1 1 0.00003 0.00012 0.00010

SumaREV d20 0073 1 -------P--------.---------.--------------------------- 0 1 0 0.00000 0.00012 0.00000

SumaREV d20 0074 1 ----------------.---------.-------------------------F- 2 1 0 0.00006 0.00012 0.00000

SumaREV d20 0076 1 ----------------.---------.I-------------------------- 0 1 0 0.00000 0.00012 0.00000

SumaREV d20 0078 1 ----------------.-----F---.--------------------------- 0 1 1 0.00000 0.00012 0.00010

SumaREV d20 0079 1 ----------------.---------.-----------------------K--- 0 1 0 0.00000 0.00012 0.00000

SumaREV d20 0080 1 ----------------.--------V.--------------------------- 0 1 70 0.00000 0.00012 0.00677

SumaREV d20 0081 1 ----------------.---K-----.--------------------------- 3 1 8 0.00010 0.00012 0.00077

SumaREV d20 0082 1 ----------------.---------.-R------------------------- 2 1 0 0.00006 0.00012 0.00000

Other day 41 sequences:

SumaREV d41 0102 1 ----------------.---T-----.--------------------L------ 0 0 1 0.00000 0.00000 0.00010

SumaREV d41 0105 1 ----------------.---------.---------------A----------- 0 2 1 0.00000 0.00025 0.00010

SumaREV d41 0109 1 -K--------------.---T-----.--------------------------- 0 0 1 0.00000 0.00000 0.00010

SumaREV d41 0110 1 ----------------.------L--.---------------------S----- 0 0 1 0.00000 0.00000 0.00010

SumaREV d41 0114 1 ----------------.-K-------.------------T-------------- 0 0 1 0.00000 0.00000 0.00010

SumaREV d41 0120 1 ----G-----------.---------.--------------------------- 0 0 1 0.00000 0.00000 0.00010

SumaREV d41 0122 1 ----------------.----K----.------------T-------------- 0 0 1 0.00000 0.00000 0.00010

SumaREV d41 0124 1 ----------------.---------.------------V-------------- 2 2 1 0.00006 0.00025 0.00010

SumaREV d41 0128 1 -----R----------.--R------.--------------------------- 0 0 1 0.00000 0.00000 0.00010

SumaREV d41 0131 1 ----------------.---K-----.---------------------S----- 0 0 1 0.00000 0.00000 0.00010

SumaREV d41 0132 1 ----------------.--R------.---------------A----------- 0 0 1 0.00000 0.00000 0.00010

SumaREV d41 0133 1 ----------------.---------.----------------S---------- 8 3 1 0.00026 0.00037 0.00010

SumaREV d41 0135 1 ----------------.R------L-.-----------S--------------- 0 0 1 0.00000 0.00000 0.00010

SumaREV d41 0137 1 ----------------.--R------.----------------------F---- 0 0 1 0.00000 0.00000 0.00010

SumaREV d41 0138 1 ----------------.---S-----.----F---------------------- 0 0 1 0.00000 0.00000 0.00010

SumaREV d41 0141 1 ------------K---.---------.--------------------------- 3 3 1 0.00010 0.00037 0.00010

SumaREV d41 0143 1 ----------------.-K-------.-----------------F--------- 0 0 1 0.00000 0.00000 0.00010

SumaREV d41 0155 1 F---------------.---------.--------------------------- 1 3 1 0.00003 0.00037 0.00010

SumaREV d41 0158 1 ----------------.---------.---------------M----------- 0 0 1 0.00000 0.00000 0.00010

SumaREV d41 0161 1 ----------------.---------.-V------------------------- 1 0 1 0.00003 0.00000 0.00010

SumaREV d41 0176 1 ----------------.---G-----.---------------------L----- 0 0 1 0.00000 0.00000 0.00010

SumaREV d41 0179 1 ----------------.---------.--------------S------S----- 0 0 1 0.00000 0.00000 0.00010

SumaREV d41 0187 1 ----------------.-K-------.------------V-------------- 0 0 1 0.00000 0.00000 0.00010

SumaREV d41 0188 1 ------T---------.-------L-.--------------------------- 0 0 1 0.00000 0.00000 0.00010

SumaREV d41 0193 1 ----------------.---T-----.-------------A------------- 0 0 1 0.00000 0.00000 0.00010

SumaREV d41 0194 1 --R-------------.---------.--------------------------- 1 1 1 0.00003 0.00012 0.00010

SumaREV d41 0199 1 ----------------.---G---L-.--------------------------- 0 0 1 0.00000 0.00000 0.00010

SumaREV d41 0203 1 ----------------.-----F---.--------------------------- 0 1 1 0.00000 0.00012 0.00010

SumaREV d41 0207 1 ----------------.---I-----.-----------S--------------- 0 0 1 0.00000 0.00000 0.00010
