# Supplementary material for: Transmission of Single HIV-1 Genomes and Dynamics of Early Immune Escape Revealed by Ultra-Deep Sequencing
Source: PLoS One. 2010 Aug 20;5(8):e12303. doi: 10.1371/journal.pone.0012303 (PMC2924888; doi:10.1371/journal.pone.0012303)
Supplement: Table S4 — Modeling results for first time point samples for WEAU, CH40 and SUMA. (0.08 MB DOC) [file pone.0012303.s005.doc]

Table S4. Modeling results for first time point samples for WEAU, CH40 and SUMA

| **SAMPLE** | **Max HD** | **N seq** | **N bases** | **λ** | **SD** | **GOF**  **P-val** | **Days (CI)** |
| --- | --- | --- | --- | --- | --- | --- | --- |
|  |  |  |  |  |  |  |  |
| **WEAU, day 10** |  |  |  |  |  |  |  |
| 454 ENV (no APOBEC) | 4 | 23346 | 129 | 0.120 | 3.06E-03 | 0.001 | 39 (38, 42) |
| 454 V3 | 4 | 3996 | 124 | 0.054 | 5.27E-03 | 0.841 | 18 (16, 22) |
| 454 ENV Lineage 1 (no APOBEC) | 4 | 22560 | 129 | 0.054 | 2.17E-03 | 0.751 | 18 (16, 19) |
| 454 ENV Lineage 2 (no APOBEC) | 2 | 813 | 129 | 0.034 | 9.10E-03 | 0.926 | 11 (7, 17) |
| **SGA ENV day 15, Keele** | 6 | 44 | 2581 | 1.34 | 0.186 | 0.462 | 23 (17, 29)  (Beast, 44) |
|  |  |  |  |  |  |  |  |
| **CH40, day 00** |  |  |  |  |  |  |  |
| 454 NEF | 4 | 4046 | 169 | 0.136 | 8.23E-03 | 0.981 | 34 (31, 38) |
| 454 V3 | 5 | 3346 | 163 | 0.195 | 1.10E-02 | 0.895 | 51 (46, 56) |
| **SGA Env day 16, Keele**  **(non-star)** | 5 | 29 | 2541 | 1.338 | 0.191 | 0.867 | 23 (17, 30)  (Beast, 26) |
|  |  |  |  |  |  |  |  |
| **SUMA day 05** |  |  |  |  |  |  |  |
| 454 REV (no APOBEC) | 4 | 31166 | 128 | 0.018 | 1.08E-03 | 0.228 | 6 (5, 7) |
| 454 TAT (no APOBEC) | 4 | 3273 | 146 | 0.024 | 3.99E-03 | 0.013 | 7 (5, 9) |
| 454 V3 (no APOBEC) | 4 | 40665 | 166 | 0.040 | 1.40E-03 | 0.891 | 11 (11, 12) |
| **SGA Env day 05, Keele**  **(no APOBEC)** | 2 | 35 | 2568 | 0.568 | 0.107 | 0.243 | 10 (6, 13)  (Beast, 18) |
|  |  |  |  |  |  |  |  |

Table S4. Modeling results for first time point samples for WEAU, CH40 and SUMA. In some subjects, there is an overall enrichment of G-to-A substitutions in the context of an Apobec3F/G motif, although no single sequence in such samples may be hypermutated (supplement refs. [4,11]). If a sample was statistically enriched for mutations in the context of Apobec motifs, all positions in the APOBEC3F/G context were removed from the alignment prior to fitting the Poisson model (such cases are marked “no APOBEC”). Fitting the Poisson model in these cases depends on this exclusion. Max HD is the maximum pairwise HD, N seq is the total number of sequences in the alignment, N bases is the length of the alignment, l is the mean of the best fitting Poisson (found through maximum likelihood; supplement ref. [12]), SD is the standard deviation on the Lambda, calculated through U-statistics methods, GOF P-val is the P value on the goodness of the Poisson fit, from a C2 statistic for dependent cells [12], and, finally, Days is the estimated days since the most recent common ancestor using our previous modeling strategy (supplement refs. [4,12]), assuming an error rate of 2.2 x 10^-5^ per base per replication, R_0_ = 6 (the reproductive ratio), and generation time of 2 days (95%confidence interval, obtained via bootstrapping, in parentheses). Results of our previous analysis of conventional full length env sequences [4] are also included for each subject, including our earlier estimates of days to the MRCA using the Bayesian evolutionary analysis program BEAST [13]; BEAST does not accommodate large enough numbers of sequences to enable its use for deep-sequencing data-sets.
