# Supplementary material for: Transmission of Single HIV-1 Genomes and Dynamics of Early Immune Escape Revealed by Ultra-Deep Sequencing
Source: PLoS One. 2010 Aug 20;5(8):e12303. doi: 10.1371/journal.pone.0012303 (PMC2924888; doi:10.1371/journal.pone.0012303)
Supplement: Table S5 — Poisson compatibility within major escape lineages. (0.09 MB DOC) [file pone.0012303.s006.doc]

**Table S5.** Poisson compatibility within major escape lineages

| **Sample, epitope variant** | **FREQ** | **Max HD** | **N seq** | **N bases** | **λ** | **SD** | **GOF pval** | **DAYS(CI)** |
| --- | --- | --- | --- | --- | --- | --- | --- | --- |
| **SUMA REV d41** |  |  |  |  |  |  |  |  |
| QRQRQIQS[L/**I**] | 0.4306 | 4 | 4794 | 157 | 0.193 | 8.976e-03 | 0.951 | 52 (48, 57) |
| QRQRQI[Q/**R**]SL | 0.2865 | 4 | 3064 | 157 | 0.130 | 9.299e-03 | 0.911 | 35 (31, 39) |
| QRQRQI[Q/**K**]SL | 0.0434 | 4 | 470 | 157 | 0.378 | 3.354e-02 | 0.579 | 101 (93, 130) |
| **SUMA TAT d41** |  |  |  |  |  |  |  |  |
| ALL Lineages |  | 7 | 20681 | 157 | 0.554 | 5.390e-03 | 0.000 | 148 (183, 190) |
| [F/**L**]HCQVCFM[T/**K**]KGLGISY | 0.0389 | 4 | 15015 | 157 | 0.050 | 2.568e-03 | 0.952 | 14 (12, 15) |
| FHCQVCFM[T/**K**]KGLGISY | 0.0122 | 6 | 4895 | 157 | 0.224 | 8.945e-03 | 0.000 | 60 (56, 65) |
| FHCQ[V/**D**]CFM[T/**K**]KGLGISY | 0.0015 | 2 | 543 | 157 | 0.072 | 5.204e-03 | 0.505 | 20 (13,27) |
| [F/**L**]HCQVCFMTKGLGISY | 0.0005 | 2 | 208 | 157 | 0.067 | 2.500e-02 | 0.931 | 18 (8, 31) |
| **WEAU ENV** |  |  |  |  |  |  |  |  |
| AE[N/**K**]LWVTVY (day 20) | 0.2294 | 2 | 439 | 148 | 0.091 | 1.986e-02 | 0.801 | 26 (17, 37) |
| AE[N/**K**]LWVTVY (day 30) | 0.0033 | 2 | 1381 | 148 | 0.065 | 9.519e-03 | 0.686 | 19 (13, 23) |
| [A/**T**]ENLWVTVY (day 20) | 0.1068 | 2 | 346 | 148 | 0.087 | 2.186e-02 | 0.849 | 25 (14, 36) |
| [A/**T**]ENLWVTVY (day 30) | 0.0007 | 2 | 653 | 148 | 0.079 | 1.518e-02 | 0.749 | 23 (15, 31) |
| **CH40 NEF** |  |  |  |  |  |  |  |  |
| SSLAFRHVA[R/**Q**] (day16) | 0.0394 | 4 | 356 | 169 | 0.404 | 4.232e-02 | 0.573 | 101 (82, 127) |
| SSLAFRHVA[R/**Q**] (day45) | 0.8329 | 5 | 1642 | 169 | 0.245 | 1.719e-02 | 0.997 | 61 (53, 70) |
| SSLAF[R/**H**]HVAR (day45) | 0.2327 | 5 | 1895 | 169 | 0.225 | 1.594e-02 | 0.720 | 56 (49, 64) |
| SSLAF[R/**H**]HVAR (day16) | 0.0854 | 3 | 173 | 169 | 0.220 | 5.037e-02 | 0.999 | 55 (29, 84) |

**Table S5. Poisson compatibility within major escape lineages.** To locate possible serial selection, for example for local compensatory mutations arising to balancing immune escape with replicative fitness, or for progressively increasing immune escape phenotypes, we examined subsets of sequences grouped according to the most common primary within-epitope mutations (see table S3 for complete translations of 454 sequences grouped by major escape forms). The headings are the same as in Table S3, and we have noted the escape amino acids in bold. The inter-sequence Hamming distance within each set was compared to our model of random and uniform mutations with no differential selection of extant forms. We found that the distributions of Hamming distances in there sets were generally consistent with the model, indicating no differential selection within each set, with exception being the SUMA Tat 3 epitope region. The SUMA Tat region contained 3 overlapping epitopes, and so was likely to have been under distinctive or more profound serial selective pressure; in this case, a double mutant in the epitope region dominated the escape forms at the third time point. Even when the Poisson fit the distribution well, the overall diversity controlling the estimate of the days to the MRCA tended to be unexpectedly large. Furthermore, the frequencies of forms dominating the sample at one time point were often strikingly different than the forms at a later time point. Since in these periods of post peak viral load and intense immune selection the viral population is not growing exponentially, but rather is shrinking rapidly, random loss of variants is expected, and the replacements observed may indicate small effective population sizes. Alternatively, the long coalescence time might be a consequence of model violations resulting from large recombination frequencies. Thus, once selection begins and multiple escape forms co-circulate at high frequency, the simple model designed for early exponential growth cannot adequately describe the complex forms that continually arise.

**SUMA REV Escape Lineages:** Pairwise Hamming Distance (HD) frequency distributions of the REV sample from patient SUMA at day 41. Represented are three common escape lineages: QRQRQIQS[L/I], QRQRQI[Q/R]SL (excluding [L/I]), and QRQRQI[Q/K]SL (excluding [L/I]). Out of all day 41 sequences, the ones that did not present escapes were analyzed for random accumulation of mutations (results not shown) and appeared to fit the Poisson model (GOF P=0.972), yielding an estimated time since MRCA of 42 days (29, 55). The third escape lineage originally gave an overestimation of the time since MRCA. This was due to the presence of a sequence repeated 61 times which had a single change GAA -> GGA, which is either an early stochastic mutation after selection began, serial selection, or a possible 454 error; given the context, a recurrent 454 error seems likely. Upon removing these sequences, the time estimate changed from 101 days to 47 days.

**SUMA TAT Escape Lineages.** Pairwise Hamming Distance (HD) frequency distributions of the TAT sample from patient SUMA at day 41. Represented are four escape lineages: [F/L]HCQVCFM[T/K]KGLGISY, FHCQVCFM[T/K]KGLGISY, FHCQ[V/D]CFM[T/K]KGLGISY, and [F/L]HCQVCFMTKGLGISY. The second lineage was the only one in this sample not to fit a Poisson model, and Tat was the only subject to have multiple mutations highly selected, possibly related to the fact that there were multiple known overlapping epitopes in this region.

**WEAU ENV Escape Lineages.** Pairwise Hamming Distance (HD) frequency distributions of the ENV sample from patient WEAU at days 20 and 30. Represented are two escape lineages: AE[N/K]LWVTVY and [A/T]ENLWVTVY. Both lineages fit the Poisson model at both time points, although diversity did not appear to increase over-time.

**CH40 NEF Escape Lineages.** Pairwise Hamming Distance (HD) frequency distributions of the NEF sample from patient CH40 at days 16 and 45. Represented are four escape lineages: SSLAFRHVA[R/Q] and SSLAF[R/H]HVAR. Both lineages fit the Poisson model at both time points although diversity did not appear to increase over time.
