# Supplementary material for: Transmission of Single HIV-1 Genomes and Dynamics of Early Immune Escape Revealed by Ultra-Deep Sequencing
Source: PLoS One. 2010 Aug 20;5(8):e12303. doi: 10.1371/journal.pone.0012303 (PMC2924888; doi:10.1371/journal.pone.0012303)
Supplement: Table S6 — Estimates of accumulation rates of dominant viral variants. (0.05 MB DOC) [file pone.0012303.s007.doc]

**Table S6.** Estimates of accumulation rates of dominant viral variants

| **Patient/Epitope** | **Sequence** | **Accumulation/loss rate (95% Cis)** | **t50** |
| --- | --- | --- | --- |
| WEAU Env AY9 | AENLWVTVY | -0.24 (-0.242, -0.229) | 28 |
| WEAU Env AY9 | AE**K**LWVTVY | 0.21 (0.199, 0.22) | 36 |
| WEAU Env AY9 | **T**ENLWVTVY | 0.14 (0.136, 0.158) | 45 |
| WEAU Env AY9 | **V**ENLWVTVY | 0.17 (0.163, 0.19) | 43 |
| WEAU Env AY9 | A**K**NLWVTVY | 0.14 (0.132, 0.157) | 48 |
| CH40 Nef SR9 | SLAFRHVAR | -0.3 (-0.324, -0.279) | 16 |
| CH40 Nef SR9 | SLAFRHVA**Q** | 0.17 (0.165, 0.177) | 34 |
| CH40 Nef SR9 | SLAF**H**HVAR | -0.04 (-0.046, -0.034) | -11 |
| Suma Rev QL9 | RQRQRQIQSL | -0.42 (-0.437, -0.403) | 34 |
| Suma Rev QL9 | RQRQRQIQS**I** | 0.4 (0.396, 0.4) | 42 |
| Suma Rev QL9 | RQRQRQI**R**SL | 0.4 (0.394, 0.396) | 43 |
| Suma Tat FY16 | FHCQVCFMTKGLGISY | -0.14 (-0.161, -0.123) | 65 |

**Table S6. Estimates of accumulation rates of dominant viral variants.**

The rate of accumulation or loss, ε, is presented with the 95% conﬁdence intervals. ε is the coeﬃcient of the time term in the exponential growth equation N = N_0_ × e^εt^. The doubling time of a variant is ln(2)/ε. The last column is the number of days expected for the variant to reach a frequency of 50%.
