# Supplementary material for: Transmission of Single HIV-1 Genomes and Dynamics of Early Immune Escape Revealed by Ultra-Deep Sequencing
Source: PLoS One. 2010 Aug 20;5(8):e12303. doi: 10.1371/journal.pone.0012303 (PMC2924888; doi:10.1371/journal.pone.0012303)
Supplement: Table S7 — Frequencies of not-transmitted B consensus amino acids. (0.06 MB DOC) [file pone.0012303.s008.doc]

**Table S7.** Alignments and frequencies of not-transmitted B consensus amino acids among all sequences, V3 and epitope regions

1. **WEAU epitope region, transmitted vs B consensus**

**B*4403**

WEAU MLLGILMICSA**AENLWVTVY**YGVPVWKEATTTLFCASDAKAYDTEVHNV

B_con ----M--------K-----------------------------------

_ count frequency

Mutation Epitope d10 d20 d30 RIER d10 d20 d30 RIER Selection

I->M No 0 0 0 814 0 0 0 0.043 No

N->K Yes 11 425 1358 2821 0.0005 0.0359 0.2298 0.1492 Yes*

* The B consensus form N->K was very common at d30, but declined in later time points, using conventional sequencing in earlier studies. The K was not detected after day 72 in earlier studies (ref: Borrow)

**B) WEAU V3 region, transmitted vs B consensus**

WEAU IRSENFTDNAKNIIVQLNVSIEINCTRPNNNTRKKITLGPG

B_con -----------T------E-V-------------S-HI---

count frequency

Mutation Epitope d10 d20 d30 RIER d10 d20 d30 RIER Selection

N->T No 0 0 4 1321 0 0 0.0010 0.3320 No

V->E No 0 0 0 93 0 0 0 0.0234 No

I->V No 0 0 0 3 0 0 0 0.0008 No

K->S No 0 0 0 1509 0 0 0 0.3792 No

T->H No 0 0 0 0 0 0 0 0 No

L->I No 0 0 0 1511 0 0 0 0.3797 No

1. **CH40 epitope region, transmitted vs B consensus**

**A*3101**

CH40 SQHGMDDPEREVLVWRFD**SSLAFRHVAR**ELHPEYYKNC

B con -L-------------K---R---H-M----------D-

count frequency

Mutation Epitope d00 d16 d45 d00 d16 d45 Selection

Q->L No 1 0 1 0.0003 0 0.0005 No

R->K No 0 17 5 0 0.0022 0.0027 No

S->R Yes 0 306 2 0 0.0400 0.0011 Yes, then declined

R->H Yes 0 1836 173 0 0.2378 0.0923 Yes, then declined

V->M Yes 0 422 1 0 0.0547 0.0005 Yes, then declined

N->D No 1 2 2 0.0003 0.0003 0.0011 No

**D)** **CH40 V3 region, transmitted vs B consensus**

CH40 KSVEITCTRPNNNTRKSIPMGPGKAFYARGDITGDIRKAYCEINGTEWHSTLKL

B con E----N------------HI---R---TT-E-I----Q-H-N-SRAK-NN---Q

count frequency

Mutation Epitope d00 d16 d45 d00 d16 d45 Selection

K->E No 0 0 1 0 0 0.0004 No

T->N No 0 0 0 0 0 0 No

P->H No 0 0 0 0 0 0 No

M->I No 3 0 0 0.0009 0 0 No

K->R No 0 0 0 0 0 0 No

A->T No 2 0 0 0.0006 0 0 No

R->T No 0 0 0 0 0 0 No

D->E No 0 0 0 0 0 0 No

T->I No 0 0 0 0 0 0 No

K->Q No 0 0 0 0 0 0 No

Y->H No 0 0 0 0 0 0 No

E->N No 0 0 0 0 0 0 No

N->S No 2 0 0 0.0006 0 0 No

G->R No 1 0 0 0.0003 0 0 No

T->A No 4 0 7 0.0012 0 0.0028 No

**E) SUMA REV epitope region, transmitted vs B consensus**

B*1402

SUMArev SEGTRQARRNRRRRWR**QRQRQIQSL**SGWILSTHLGRPAEPVPLQLPPLERLT

B_con P---------------E-----R-I-E-----Y-------------------

count frequency

Mutation Epitope d05 d20 d41 d05 d20 d41 Selection

S->P No 3 0 42 0.0001 0 0.0041 No

Q->E Yes 1 0 0 0 0 0 No

Q->R Yes 2 3 3076 0 0.0004 0.2974 Yes

L->I Yes 1 1 4786 0 0.0001 0.4627 Yes

G->E No 6 2 18 0.0002 0.0003 0.0017 No

H->Y No 0 1 0 0 0.0001 0 No

**F) SUMA TAT epitope region, transmitted vs B consensus**

**B*1501**

___________

__________

SUMAtat PGSQPKTACTTCYCKKCC.FHCQVCF**MTKGLGISY**.GRKKRRQRRRAPQDSQNH

B_con ----------N-------.-------I--------.----------------T-

count frequency

Mutation Epitope d05 d20 d41 d05 d20 d41 Selection

T->N No 0 0 2 0 0 0 No

M->I Yes 1 2 18 0.0003 0.0003 0 No

N->T No 0 3 14 0 0.0004 0 No

**G) SUMA V3 region, transmitted vs B consensus**

SumaV3 KNFTNNANIIIVQLNDSVEINCTRPNNNTRKSIPIGPGRAFYTTGEIIGDIRQAH

B_con E---D--KT------E-----------------H---------------------

count frequency

Mutation Epitope d05 d20 d41 d05 d20 d41 Selection

K->E No 2 1 5 0.0001 0 0.0003 No

N->D No 6 2 73 0.0002 0 0.0045 No

N->K No 0 0 0 0 0 0 No

I->T No 0 3 0 0 0.0001 0 No

D->E No 13 0 1 0.0003 0 0.0001 No

P->H No 0 0 0 0 0 0 No

**Table S7. Alignments and frequencies of not-transmitted B consensus amino acids among all sequences, V3 and epitope regions. A full accounting of the frequencies.** Alignments and frequencies of not-transmitted B consensus amino acids among all sequences, V3 and epitope regions. A full accounting of the frequencies. A full accounting of the mutations towards the consensus in these regions is given for the sites where a non-consensus form is transmitted or dominates the sample. As in Fig. 8, the epitope region substitutions are shown in red, the non-epitope regions in blue, and chronic infection in green. Parts A-G correspond to the 7 regions that were sequenced in this study.
