# Supplementary material for: Transmission of Single HIV-1 Genomes and Dynamics of Early Immune Escape Revealed by Ultra-Deep Sequencing
Source: PLoS One. 2010 Aug 20;5(8):e12303. doi: 10.1371/journal.pone.0012303 (PMC2924888; doi:10.1371/journal.pone.0012303)
Supplement: Table S8 — Summary of subtype consensus frequencies in 4 chronically infected subjects from an earlier study [supplement ref. 10, Tsibris et al.]. (0.05 MB DOC) [file pone.0012303.s009.doc]

**Table S8.** Summary of subtype consensus frequencies in 4 chronically infected subjects from an earlier study [10].

**Subject 07, Total number of pre-treatment sequences: 112,818**

07con MCTRPGNNTRKSTRIGPGQTFFATGDIIGDIRQAHCNIS

C_con V----N------I--------Y-----------------

Mutation Count Frequency

M->V 99 0.001

G->N 0 0

T->I 35,676 0.32

F->Y 6 0.0001

**Subject 18, Total number of pre-treatment sequences: 110,471**

18con TCIRPNNNTRKSISIGPGRAFYTTGEIIGDIRQAHCNIS

Bcon N-T----------H-------------------------

Mutation Count Frequency

T->N 7 0.0001

I->T 138 0.001

S->H 0 0

**Subject 19, Total number of pre-treatment sequences: 59,226**

19con NCIRPNNNTRKGIHLGPGAFYATDNIGDIRQAHCNIS

Bcon --T--------S--I------T-GE------------

Mutation Count Frequency

I->T 691 0.01

G->S 22 0.0004

L->I 20 0.0003

A->T 675 0.01

D->G 137 0.002

N->E 0 0

**Subject 47, Total number of pre-treatment sequences: 32,203**

47con NCTRPNNNTRKSINIGPGSAWYTTGDIIGDIRQAHCNIS

Bcon -------------H----R-F----E-------------

Mutation Count Frequency

N->H 11,840 0.37

S->R 8 0.0002

W->F 3 0.0001

D->E 4 0.0001

**Table S8. Summary of subtype consensus frequencies in 4 chronically infected subjects from an earlier study (supplement ref. [10]).** In 4 chronic-infection subjects, 17 non-subtype consensus amino acids were found: 2 were common (found in ~1/3 of the sequences); 5 were clearly replicating at moderate levels (>0.1%); 7 were recurrent (0.1-0.01%), but rare; and only 3 were not found at all. Subject 07 had a chronic C subtype infection, the other 3 were B subtype infections.
