# Supplementary material for: Transmission of Single HIV-1 Genomes and Dynamics of Early Immune Escape Revealed by Ultra-Deep Sequencing
Source: PLoS One. 2010 Aug 20;5(8):e12303. doi: 10.1371/journal.pone.0012303 (PMC2924888; doi:10.1371/journal.pone.0012303)
Supplement: Table S9 — Inner PCR/sequencing primers (forward, F; reverse, R). (0.09 MB DOC) [file pone.0012303.s010.doc]

Table S9. Inner PCR primers (with 454 adaptors and DNA barcodes).

| **CH40** |  |
| --- | --- |
| ***env V3*** |  |
| CH4V3-F: | 5’-GCCTCCCTCGCGCCATCAG–NNNN–GACAATGCTAAAACAATAATAGTAC |
| CH4V3-R: | 5’-GCCTTGCCAGCCCGCTCAG–NNNN–TTWTTATATTGTTCTCTTAATTTTTYAAC |
| d00: | ATAC |
| d16: | ACGA |
| d45: | CTAT |
| Control: | CGAT |
|  |  |
| ***nef* epitope region** |  |
| CH4SL10-F: | 5’-GCCTCCCTCGCGCCATCAG–NNNN–GGAGAGAACAACTGCTTGTTACACCC |
| CH4SL10-R: | 5’-GCCTTGCCAGCCCGCTCAG–NNNN–GGGCTCGCCACTCCCCAATTCCGCCC |
| d0: | TACA |
| d16: | TCGA |
| d45: | ATAT |
| Control: | TGAT |
|  |  |
| **WEAU & RIER** |  |
| ***env V3 region*** |  |
| WRV3-F: | 5’-GCCTCCCTCGCGCCATCAG–NNNN–TAAATGGCAGTCTAGCAGAARAAG |
| WRV3-R: | 5’-GCCTTGCCAGCCCGCTCAG–NNNN–CTTATRTYTCCTATTATTTBTCCTGTTG |
| RIER: | ATAG |
| WEAU d10: | ACGA |
| WEAU d20: | CTAG |
| WEAU d30: | TGCA |
| Control: | CGAG |
|  |  |
| ***env* epitope region** |  |
| WRAY9-F: | 5’-GCCTCCCTCGCGCCATCAG–NNNN–ATGARAGKGAAGGGGATCAGGAAG |
| WRAY9-R: | 5’-GCCTTGCCAGCCCGCTCAG–NNNN–GTGGGTTGRGGTCTGTGGGTACAC |
| RIER: | AGTC |
| WEAU d10: | ATGT |
| WEAU d20: | CGAT |
| WEAU d30: | TACT |
| Control: | CTAC |
|  |  |
| **SUMA** |  |
| ***env* V3 region** |  |
| SV3.2x-F: | 5’-GCCTCCCTCGCGCCATCAG–NNNN–GGCAGTCTAGCAGAAGAAGAAGTAG |
| SV3.2x-R: | 5’-GCCTTGCCAGCCCGCTCAG–NNNN–GTGTCATTCCATTTGCTTTTACTAATG |
| d5: | ATGC |
| d20: | TGCA |
| d41: | CATG |
| Control: | ACAC |
|  |  |
| ***rev* epitope region** |  |
| SREV.1x-F: | 5’-GCCTCCCTCGCGCCATCAG–NNNN–TCACCATTATCGTTCCAGACCCGCC |
| SREV-1x-R: | 5’-GCCTTGCCAGCCCGCTCAG–NNNN–GAAGTTCCACAATCCTCGTTACAATC |
| d5: | CGTA |
| d20: | GTAC |
| d41: | TACG |
| Control: | GCGC |
|  |  |
| ***tat* epitope region** |  |
| STAT.11-F: | 5’-GCCTCCCTCGCGCCATCAG–NNNN–AATGGAGCCAGTAGATCCTAACC |
| STAT.11-R: | 5’-GCCTTGCCAGCCCGCTCAG–NNNN–GCTTGCATTACATATACTACTTAC |
| d5: | CATG |
| d20: | GCAT |
| d41: | TGCA |
| Control: | TCTC |

Table S9. Inner PCR primers. Forward (F) and reverse (R) are indicated. For each time point, the 4 bases specified are the tags that allowed multiplexing.
