# Supplementary figures and images for: Transmission of Single HIV-1 Genomes and Dynamics of Early Immune Escape Revealed by Ultra-Deep Sequencing
Source: PLoS One. 2010 Aug 20;5(8):e12303. doi: 10.1371/journal.pone.0012303 (PMC2924888; doi:10.1371/journal.pone.0012303)

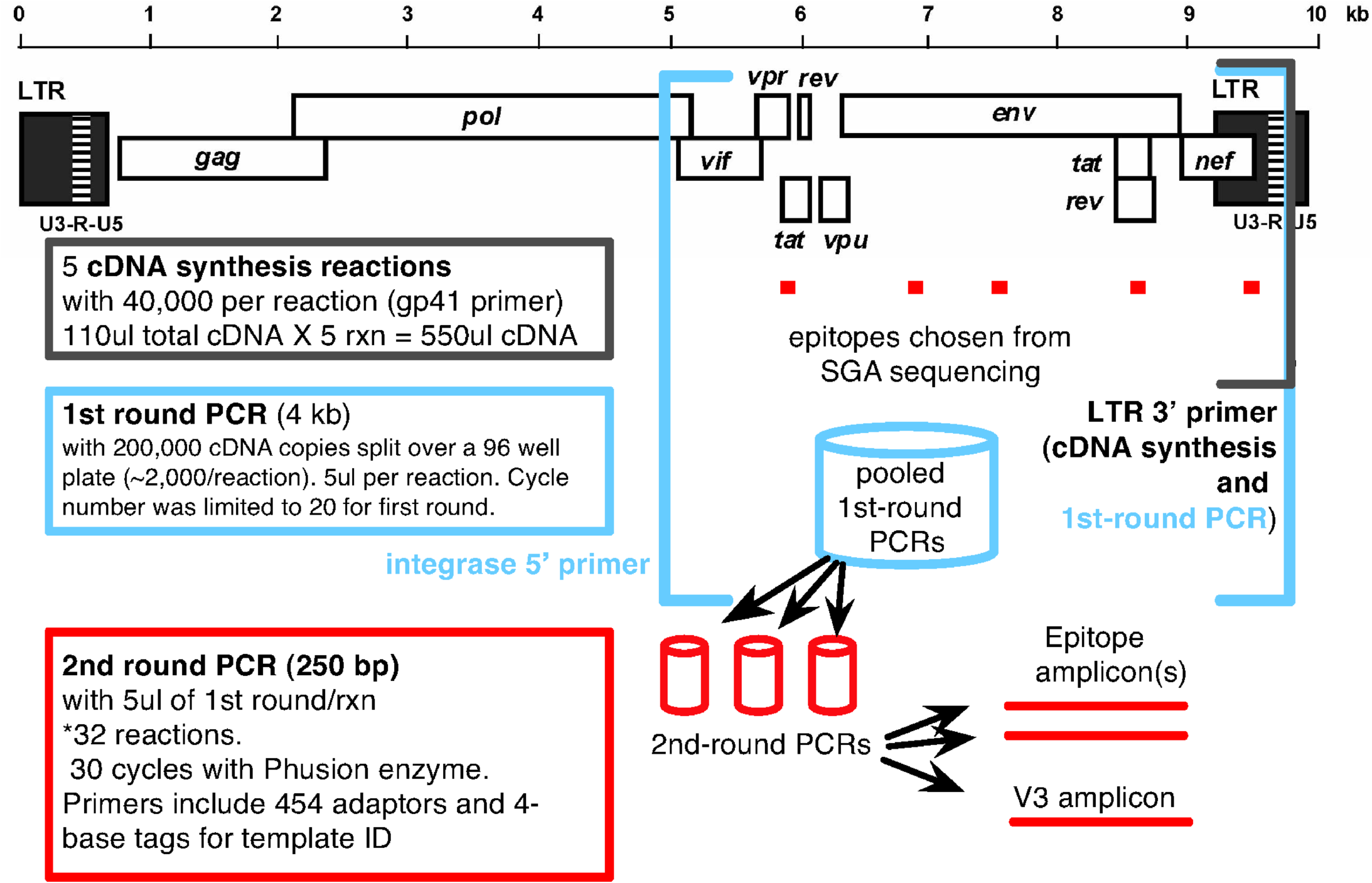

Supplement: Figure S5 — Amplification protocol. The protocol was designed with the intent of reducing loss of diversity during PCR amplification by (1) limiting the number of cycles (2) using large amounts of template, and (3) using multiple small amplification reactions which were pooled for sequencing. (0.77 MB TIF) [file pone.0012303.s015.tif]
